# Green synthesis, anti-proliferative evaluation, docking, and MD simulations studies of novel 2-piperazinyl quinoxaline derivatives using hercynite sulfaguanidine-SA as a highly efficient and reusable nanocatalyst†

**DOI:** 10.1039/d3ra03305h

**Published:** 2023-08-23

**Authors:** Zohreh Esam, Malihe Akhavan, Atefeh Mirshafa, Ahmadreza Bekhradnia

**Affiliations:** a Pharmaceutical Sciences Research Center, Student Research Committee, Department of Medicinal Chemistry, Faculty of Pharmacy, Mazandaran University of Medical Sciences Sari Iran; b Pharmaceutical Sciences Research Center, Department of Medicinal Chemistry, Mazandaran University of Medical Sciences Sari Iran a.bekhradnia@gmail.com; c Ramsar Campus, Mazandaran University of Medical Sciences Ramsar Iran

## Abstract

In this study, the immobilization of sulfaguanidine-SA on the surface of FeAl_2_O_4_ (hercynite) MNPs (magnetic nanoparticles) as a novel acid nanocatalyst has been successfully reported for the synthesis of 2-(piperazin-1-yl) quinoxaline derivatives *via* a one-pot multiple-component reaction under green conditions. The products were characterized by SEM, TEM, TGA, EDS, BET technique, VSM, and FTIR. This series of novel 2-piperazinyl quinoxaline derivatives containing isatin-based thio/semicarbazones and/or Schiff bases of Metformin were evaluated for anticancer activity against both human ovarian and colon-derived tumor cell lines by MTT colorimetric assay. Although most of the investigated hybrid compounds exhibited excellent anti-proliferative activities and high selectivity index (SI) values, the promising compounds *N*′-[4-(quinoxaline-2-yl)-piperazine-1-yl]methyl-5-chloro-1-*H*-indole,2,3-dion-3-metformin 4c and *N*′-[4-(quinoxaline-2-yl)-piperazine-1-yl]methyl-5-bromo-1-*H*-indole,2,3-dion-3-metformin 4b proved to be the most potent anti-proliferative agents (IC50 values < 1 μM). Molecular docking and dynamics simulation suggest that these hybrid compounds can be wrapped in the catalytic cavity of c-Kit tyrosine kinase receptor and the binding pocket of P-glycoprotein with high scores. Thus, 2-piperazinyl quinoxaline linked isatin-based *N*-Mannich bases of metformin and/or thio/semicarbazones might be served as suitable candidates for further investigations to develop a new generation of multi-target cancer chemotherapy agents.

## Abstract 2

The excellent catalytic activity of newly magnetic nanocatalyst FeAl_2_O_4_@PTMS-sulfaguanidine-SA in a one pot multicomponent Mannich-type reaction was used to obtain 2-piperazinyl quinoxaline derivatives with highly selective anti-proliferation efficacy.

## Graphical abstract

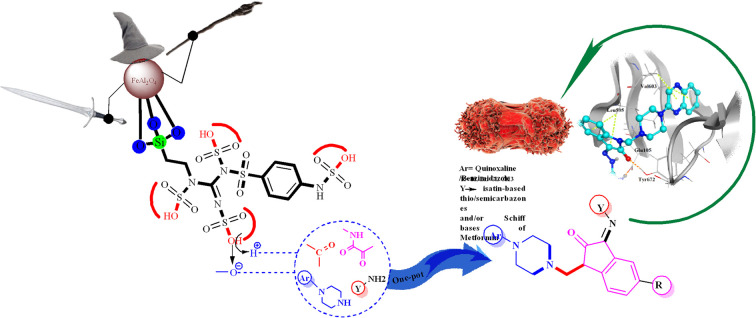

## Introduction

The nitrogen-based heteroaromatic ring quinoxaline, as a bioisoster of privileged functional scaffolds quinoline, benzimidazole, benzothiophene, has been found to have a wide variety of biological and therapeutic applications.^1,2^ Numerous synthetic quinoxaline derivatives, particularly 2-amino quinoxaline containing compounds, have revealed various remarkable medicinal roles such as anti-proliferative agents, specially *via* kinase-inhibitor activities.^3,4^ For instance, working on 2-piperazynyl quinoxalines as c-Met receptor tyrosine kinases (RTKs) efficient inhibitors has revealed that it is important to have a basic, preferably tertiary nitrogen substituent at the quinoxaline 2-position.^3^

**Fig. 1:**
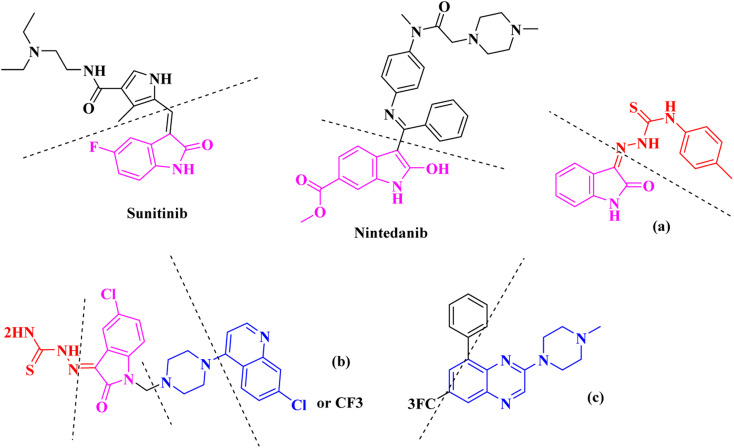
Some of the selected previously reported compounds/approved drugs possessing common structural features and biological activity with the tested ligands in this work. Sunitinib and nintedanib as tyrosine kinase inhibitors, isatin-based thio/semicarbasones (a) as efficient agents against P-gp-expressing cancer cells, 4-piperazinylquinoline linked isatin compounds (b) with confirmed anti-breast cancer agents.

On the other hand, Isatin (1*H*-indole-2,3-dione, indoline-2,3-dione) with a wide range of pharmaceutical uses, is present as a functional portion in the chemical structures (Fig. 1) of the approved antitumor and anti-angiogenetic agents sunitinib and nintedanib which are considered as multi-target tyrosine kinase inhibitors.^5^ Besides, isatin-based thio/semicarbasones can also selectively kill cells over-expressing the efflux transporter P-glycoprotein, which were found to be cytotoxic in multidrug-resistant (MDR) cancers.^6^

Apart from the clinical strategy, polychemotherapy development of hybrid molecules with more than one functional portion and biological target^7^ can be considerrd one of the most promising strategies to overcome cancer. For instance, a class of 4-piperazinylquinoline derivatives bearing isatin-based β-thiosemicarbazones demonstrated selective anti-cancer activity^8,9^ and this can be considered as inspiring research in this area (Fig. 1).

**Fig. 2:**
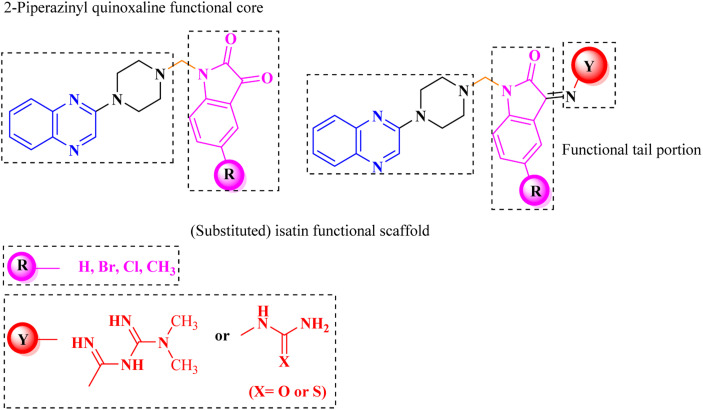
General structure of the evaluated hybrid molecules in this study. Here, quinoxaline-containing hybrids of isatin-based thio/semicarbazones have been introduced as multiple pharmacophore biologically active molecules. Metformin with recently confirmed anti-cancer properties is also inserted in this drug design process as a functional scaffold.

Aside from the 2-piperazynyl quinoxaline core and isatin-bases thio/semicarbazone scaffold, Metformin (dimethylbiguanide) not only as a proven anti-hyperglycemic drug has demonstrated an unexpected anticancer activity,^10^ but also as a functional fragment of bioactive agents showed remarkable medicinal properties. Metformin with a terminal primary amine seems to be active towards any electrophilic center *i.e.* the carbonyl carbon of aldehydes of the resulted Schiff base products have received an increasing attention due to their pharmacological effects over recent decades.^11^ Thus, in the current work, we have reported a new green synthesis procedure as well as biologically evaluation of hybrid molecules composed of various isatin-based Schiff bases and 2-piperazinyl quinoxaline core (Fig. 2) as potential anti-cancer agents.^12^

What adds to the importance of our research is describing a green synthesis route for preparing of a leak-free and highly efficient multifunctional heterogeneous magnetic nanocatalyst *via* grafting sulfonic groups onto the surface of FeAl_2_O_4_ MNPs coated with a novel Schiff-base layer consisting of CPTMS (3-chloropropyl trimethoxysilane) and sulfaguanidine as an efficacious bridge. The latter portion has two functional heads: its COOH moiety, which has a strong affinity to FeAl_2_O_4_ surface hydroxyl groups, and NH groups for the grafting of multi-SO_3_H functionalities. Then, we investigated the utility of the newly synthesized organic–inorganic hybrid (FeAl_2_O_4_@PTMS-sulfaguanidine-SA) as a core–shell-structured nanocatalyst for the synthesis of novel quinoxaline-containing hybrides *via* a multicomponent one-pot cascade reaction. Based on the logic governing the structure design of these hybrid ligands, molecular docking (MD) and molecular dynamics simulation studies on P-glycoprotein transporters and c-Kit receptor tyrosine kinase respectively, were used to probe the mechanisms of their observed significant anti-proliferative activities.

## Experimental

### Chemistry

#### Nanocatalyst preparation

The FeAl_2_O_4_ was prepared *via* a chemical co-precipitation method.^13^ Initially, FeCl_2_·4H_2_O (0.795 g) and Al (NO_3_)_3_·9H_2_O (0.375 g) (2 : 1 mol) were dissolved in water (100 ml) under N_2_ atmosphere at 80 °C. Then, 10 ml of NaOH solution (0.2 M) was added dropwise for 10 min into the stirring mixture (the pH of solution was found to be 12). After 30 min stirring of mixture, the FeAl_2_O_4_ MNPs were collected using an external magnet and precipitate was washed a few times with deionized water. The obtained precipitate was dried at 75 °C overnight.

**Scheme 1:**
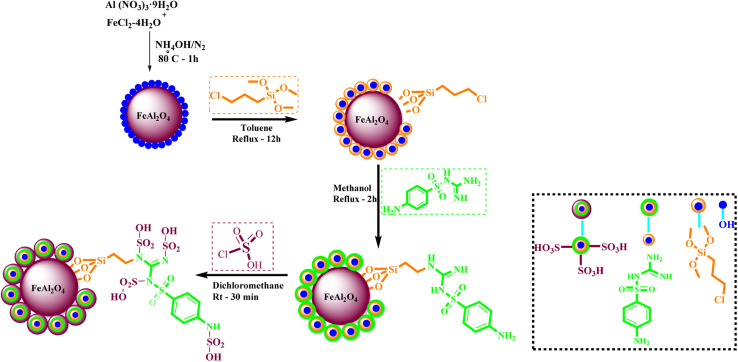
Preparation of the FeAl_2_O_4_@PTMS-sulfaguanidine-SA nanocatalyst.

In the following step, the FeAl_2_O_4_ MNPs were supported with CPTMS (3-chloropropyltrimethoxysilane) and sulfaguanidine, respectively. In subsequent, the FeAl_2_O_4_@PTMS-sulfaguanidine MNPs were acidic with chlorosulfonic acid. For the identification, FeAl_2_O_4_@PTMS-sulfaguanidine-SA nanoparticles used FT-IR, SEM, TEM, BET, EDX, XRD, TGA and VSM analyses (Scheme 1). The characterization data of the final product was confirmed by FT IR spectra, SEM, TEM, EDX, XRD, TGA, VSM, and BET. The corresponding results as well as recycling studies and hot filtration test are available within the related article or the ESI† (Section 1.3).^14^

## General procedure for the synthesis of Mannich bases 2a–2d, 3a–3h and 4a–4d, secondary amine-containing compounds using FeAl_2_O_4_@PTMS-sulfaguanidine

**Scheme 2:**
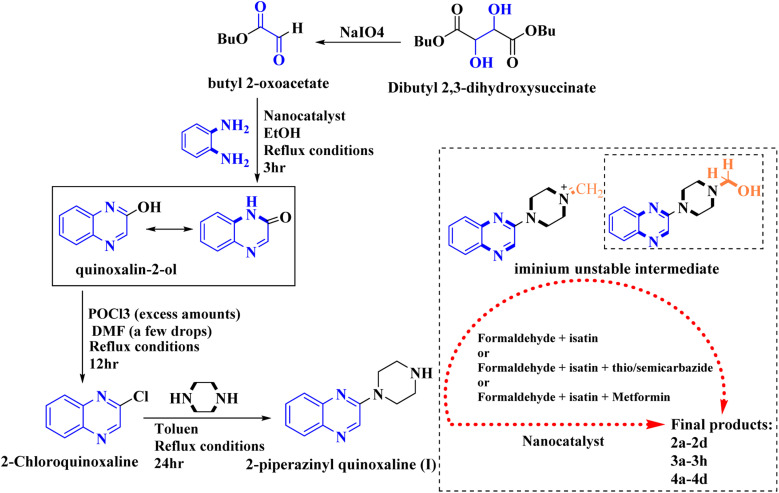
Typical experimental procedure for the synthesis of quinoxaline-based intermediate compound I. The final products (series A: (2a–2d), B: (3a–3h), and C: (4a–4d)) were synthesized through two different methods (*via* a one-pot multicomponent reaction in the presence of our newly synthesized nanocatalyst or *via* formation of iminium unstable intermediate in the absence of the nanocatalyst (ESI†).

**Scheme 3:**
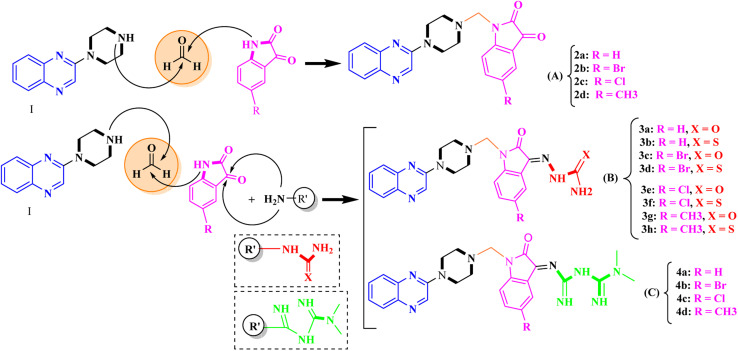
General synthesis pathway of the evaluated hybrid *N*-Mannich bases (2a–2d, 3a–3h, and 4a–4d). The final products are derived from 2-piperazinyl quinoxaline as the core scaffold, attached to the isatin-based Schiff bases of metformin and/or thio/semicarbazones. As it can be seen, metformin with a terminal primary amine seems to be active towards any electrophilic center *i.e.* the carbonyl carbon of aldehydes.

**Scheme 4:**
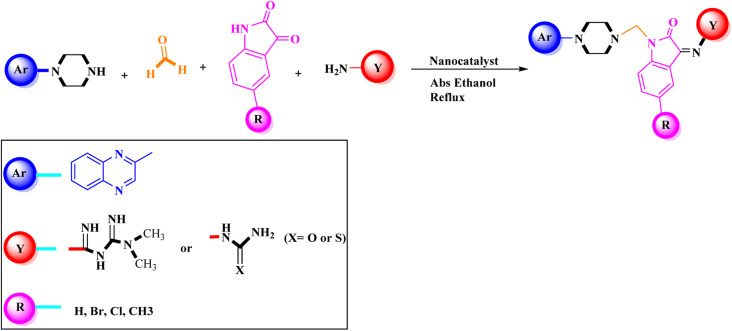
Synthesis of 2-piperazinyl quinoxaline derivatives by FeAl_2_O_4_@PTMS-sulfaguanidine-SA MNPs.

In the current study, the evaluated *N*-Mannich bases (derived from NH-containing heterocycle isatin) were synthesized in our recently published research *via* a one-pot green approach using a newly designed acidic nanocatalyst.^14,15^ As the results of our previous study, in the first step, a mixture of sodium metaperiodate (NaIO_4_) was used for the oxidative cleavage of butyl-2,3-hydroxysuccinate into butyl-2-oxoacetate. Then, a one-step method was employed to directly convert the butyl-2-oxoacetate into quinoxaline-2-ol in ethanol and reflux condition. Subsequently, 2-chloroquinoxaline was obtained through the addition of phosphoryl chloride to quinoxaline-2-ole in the presence of a catalytic amount of DMF. Finally, followed preparation of 2-piperazinyl quinoxaline (I) with piperazine in toluene, the desired final products (2-piperazinyl quinoxaline derivatives) were produced in a novel and one-step method in the presence of nanocatalyst FeAl_2_O_4_@PTMS-sulfaguanidine-SA MNPs (40 mg).^16^Schemes 2–4 illustrated the general synthesis pathway of compounds 2a–2d, 3a–3h, and 4a–4d (series A, B, and C respectively) from the main initial substrate 2-piperazinyl quinoxaline (compound I). The structures of the final products were confirmed by the elemental and spectral analyses which the corresponding results are available within the related article^15–17^ or the ESI (Fig. S1).†

## Cytotoxicity MTT assay

The anti-proliferative activity of *N*-Mannich bases 2a–2d, 3a–3h, and 4a–4d were investigated at five different concentrations of 1.5, 3, 6, 12, 24 μg ml^−1^ against tow human cancer cell lines; HCT-116 and SCOV3 (colon and ovarian cancer respectively) by the standard MTT assay *in vitro*, with imatinib as positive control and vehicle dimethyl sulfoxide (DMSO) as negative control. The tested cells were cultured in medium and dialyzed fetal bovine serum (FBS) and then as suspended germs in Minimum Essential Medium (MEM) were seeded into 96-well plates (at the density of 10 × 10^4^ viable cells per well). After 24 h incubation in 5% CO_2_ at 37 °C and cell attachment. Then the evaluated compounds (at 0.1–250 μg ml^−1^ concentrations) were added to the cultured cells and incubated for another 24/48 h.^18,19^ After washing of the cell containing plates with FBS, 20 μl of fresh 4,5-(dimethylthiazol-2-yl)-2,5-diphenyl tetrazolium bromide (MTT) solution (5 mg ml^−1^) was added to each well, and incubated with cells for additional 4 h in the same conditions. Then, the medium was discarded and approximately 70 μl of DMSO was used and the resulted solution was vigorously mixed to dissolve and remove the residual tetrazolium and purple formazan crystals in each well. Next, the absorbance of each well was measured at 492 nm and 630 nm (for absorbance of MTT formazan and the reference wavelength respectively) with an ELISA plate reader. Stock solutions of all the tested compounds were prepared in DMSO, while the final dilutions were made with distilled water in order to maintain a maximum concentration of 0.5% DMSO per well. All the evaluated compounds were also tested for cytotoxicity on HFF normal cells at the above-mentioned concentrations and in the same conditions and incubation periods of time. Curves were fit using Graph Pad Prism as described in the Methods section.^19^

## Molecular docking studies

Protein-ligand docking was initiated using LeDock software (http://lephar.com). The structure of all the tested compounds were sketched using HyperChem.^20^ The geometry and energy of the structures were being optimized using ORCA software^21^ at DFT, B3LYP/cc-pvdz level of theory. The chain A of structure of c-Kit PDB ID 1T46 was selected for docking,^22^ cognate ligand and all crystallographic water molecules removed from the macromolecular structure using the LePro module (http://lephar.com). The docking parameters are set in a way that the center box of docking centered on of the CB atom of Val603 as this is the one of the key residues in the active site of c-Kit enzyme. The grid box was set to 16 × 16 × 16 with a spacing value of 1.0 Å and the number of binding poses was set to 100. The best conformation with least binding energy and better interacting residues was selected.

## Molecular dynamics simulations

In order to explore which part of the ligands is responsible for their layout in the active site of the enzyme and consequently their inhibitory activities, the three compound were chosen from the best docking pose from each different subsets. Each of the three chosen ligands as well as the crystallographic one as a control bound to the protein has been simulated for a period of 100 ns molecular dynamic simulation in explicit water. For each ligand, full Amber topology/coordinate files were created using the AmberTools package. Using the antechamber program of AmberTools The VDW and bonded parameters for the ligands were taken from the general amber force field (GAFF). Partial atomic charges were then assigned based on the RESP charge Derive Server.^23^ The AMBER format files of ligands were converted to the GROMACS format using the ACPYPE python tool. Each of the complexes was solvated in TIP3P water model and ions were introduced into the system to make the whole system neutral. All MD simulations were carried out by the GROMACS 2020.1 package. Amber99sb-ildn force field from the investigation of Beauchamp *et al.*^24^ was chosen to describe the protein behavior. Periodic boundary conditions were applied in all three directions of space. Each system became energy minimized with steeped descent algorithm. After minimization, the NVT followed by NPT ensemble were applied while the Ca atoms of protein were position restrained. The NVT ensemble was adopted at constant temperature of 300 K and with a coupling constant of 0.1 ps in modified Berendsen thermostat with time duration of 500 ps. After stabilization of temperature, 1 ns NPT simulation was performed in which Berendsen barostat was employed with a coupling constant of 2.0 ps at 1 bar. The production run in NPT and the time step of 2 fs was the followed step where the restraint was removed. The particle mesh Ewald (PME) method interaction was used for long range electrostatic. A 12 Å cutoff for long-range and the LINKS algorithm for H-bond constraints were applied in both the equilibration and production run. One of the main approaches to estimate binding affinity/free energy of inhibitors in the binding site of protein is using MM-PBSA method.^25^ This calculation method can be seen in ESI.†

## Results and discussion

### Evaluation of the catalytic activity of FeAl_2_O_4_@PTMS-sulfaguanidine-SA MNPs through the synthesis of novel 2-piperazinyl quinoxaline derivatives

After characterizing the synthesized FeAl_2_O_4_@PTMS-sulfaguanidine-SA MNPs, its catalytic activity was evaluated in Mannich reaction. The catalytic activity of FeAl_2_O_4_@PTMS-sulfaguanidine nanocatalyst has also been investigated in the reaction of a three-component coupling of 2-(piperazin-1-yl) quinoxaline (IV) (1 mmol), formaldehyde (37%), and isatin (1 mmol) as our first model reaction (Table 1). To optimize the reaction parameters, the effects of catalyst quantity, reaction temperature, and solvent were investigated.

**Table 1:** Model reaction 1: optimizing the model reaction conditions for the synthesis of the compound 6a^a^

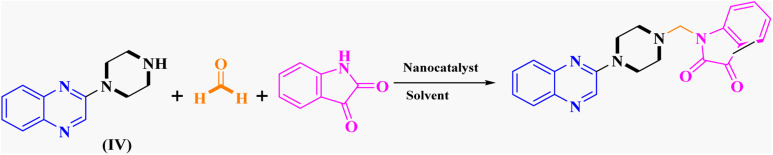					
Entry	Catalyst (g)	Solvent	Temperature (°C)	Time (min)	Yield^b^ (%)
1	—	Ethanol	50, reflux	24 h	—
2	FeAl_2_O_4_ (50 mg)	Ethanol	50, reflux	24 h	—
3	Nanocatalyst (10 mg)	Ethanol	Reflux	1 h	37%
4	Nanocatalyst (20 mg)	Absolute ethanol	Reflux	1 h	60%
5	Nanocatalyst (30 mg)	Absolute ethanol	Reflux	1 h	75%
6	**Nanocatalyst (40 mg)**	**Absolute ethanol**	**Reflux**	**1 h**	**96%**
7	Nanocatalyst (80 mg)	Absolute ethanol	Reflux	1 h	95%
8^c^^,^^d^	GAA (a few drops)	Ethanol	Reflux	Over night	Trace
9	Recovered nanocatalyst	Absolute ethanol	Reflux	1 h	95

a Reaction conditions: 2-(piperazin-1-yl) quinoxaline (IV) (1 mmol), formaldehyde (1 mmol), and isatin (1 mmol), and catalyst in solvent.

b Isolated yield after recrystallization.

c Hard workup.

d Unwanted byproduct.

The reaction was performed in the absence of the nanocatalyst, and poor results were obtained. Different amounts of FeAl_2_O_4_@PTMS-sulfaguanidine-SA were studied at 50 °C and 100 °C in (absolute) ethanol as a benign polar and protic solvent, and the product yields increased from 37% to 96% by increasing the catalyst loading from 10 to 40 mg (Table 1, entries 3–6). However, no changes in yields were observed when the amounts of the nanocatalyst increased to 80 mg (Table 1, entry 7). As expected, as the temperature dropped, reaction yields generally decreased. In absolute ethanol, the serious problem of unwanted by products was less annoying compared to ethanol. These were considered as optimum conditions for this kind of three-component reaction (Table 1, entry 6).

In order to improve the reaction parameters, the condensation of 2-(piperazin-1-yl) quinoxaline (IV) (1 mmol), formaldehyde (37%), isatin, and semicarbazide (1 mmol) was performed to afford compound 7a. The results are shown in Table 2. Absolute ethanol was recommended as the best solvent by optimization studies because of its high yield, eco-friendliness, affordability, and green attributes. Also, higher catalyst amounts did not affect the yield significantly. Accordingly, the optimum conditions for this one-pot protocol are summarized in Table 2, entry 10 (95% yield).

**Table 2:** Model reaction 2: optimizing the model reaction conditions for the synthesis of the compound 7a^a^

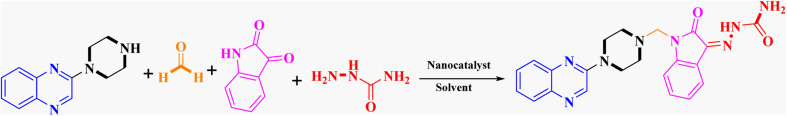					
Entry	Catalyst (g)	Solvent	Temperature (°C)	Time (min)	Yield^a^^,^^b^ (%)
1^d^	—	Ethanol	Rt & reflux	48 h	—
2	Nano-Fe_3_O_4_ (50 mg)	Ethanol	Rt & reflux	48 h	—
3	Nanocatalyst^e^ (10 mg)	Ethanol	Reflux	1 h	40
4	Nanocatalyst (10 mg)	Absolute ethanol	Reflux	1 h	48
5	Nanocatalyst (30 mg)	Absolute ethanol	Reflux	1 h	65–70
6	**Nanocatalyst (40 mg)**	**Absolute ethanol**	**Reflux**	**1 h**	**96**
7	Nanocatalyst (40 mg)	Ethanol	Reflux	1 h	60
8	Nanocatalyst (80 mg)	Absolute ethanol	Reflux	1 h	95
9^c^^,^^d^	GAA (a few drops)	Absolute ethanol	Reflux	Over night	Trace
10	Nanocatalyst recover	Absolute ethanol	Reflux	1 h	95

a Reaction conditions: 2-(piperazin-1-yl) quinoxaline (IV) (1 mmol), formalin (37%), isatin and semicarbazone (1 mmol).

b Isolated yield after recrystallization.

c Hard workup.

d Unwanted byproduct.

e FeAl_2_O_4_@PTMS-sulfaguanidine-SA MNPs.

Metformin and a variety of (substituted) isatins were used as the reaction substrates to create the corresponding Schiff Mannich bases in the presence of FeAl_2_O_4_@PTMS-sulfaguanidine-SA at reflux temperature for approximately 1 hour (90–99% yields). The results are depicted in Scheme 4 and Table 2.

Schema S1† depicts one of the proposed reaction pathways for the Mannich reaction in the presence of FeAl_2_O_4_@PTMS-sulfaguanidine-SA as a heterogeneous Lewis acid catalyst, even though the precise mechanism of this process is not entirely clear. FeAl_2_O_4_@PTMS-sulfaguanidine-SA heterogeneous nanocatalyst's surface contains electron-deficient sites that may coordinate with the O-donor sites of formaldehyde and (substituted) isatin. Due to coordination with the empty orbital of the –SO_3_H group on the surface of the functionalized FeAl_2_O_4_ (ref. 26) the electrophilicity of the carbonyl carbon atom of both formaldehyde and isatin increases.^27^ Subsequently, the Mannich reaction leads to the corresponding Mannich base product.

## Comparison

To evaluate the efficiency of this acidic heterogeneous organic–inorganic nanocatalyst and methodology, the obtained results show that FeAl_2_O_4_@PTMS-sulfaguanidine-SA as an efficient catalyst increases the reaction yields and rate with simple conditions, simple workup, and reduced unwanted by-product (Table 3). As shown in the table, with the cyclocondensation of OPD with butyl 2-oxoacetate (model reaction 1) in the presence of FeAl_2_O_4_@PTMS-sulfaguanidine-SA as the catalyst, higher yields of products were obtained in shorter reaction time and under milder conditions.

**Table 3:** Comparative study the present method with previous works in preparation of quinoxaline-2-ol (compound II)

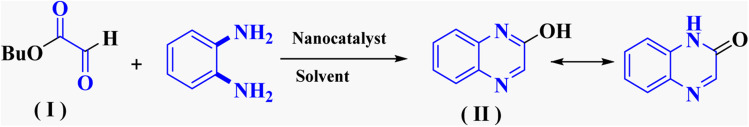						
Entry	Catalyst	Solvent	Reaction condition	Time (h)	Yield and product	Ref.
1	—	Methanol, toluene	Reflux	2 h	85% compound (II)	28
2	Alkaline H_2_O_2_	Water	Reflux	8 h	86% compound (II)	29
3	Selenium(iv) oxide	Water	Reflux	5 h	13% compound (II)	30
4	Sodium hydride	Dioxane	Rt	16 h	45% compound (II)	31
5	Sodium hydroxide	DMF	Heating	8 h	63% compound (II)	32 and 33
6	Nanocatalyst (40 mg)	Ethanol	Reflux	1 h	90% compound (II)	This work

The optimal reaction conditions were used to produce the corresponding Schiff Mannich bases in the presence of FeAl_2_O_4_@PTMS-sulfaguanidine-SA at reflux temperature for about 1 h (90–99% yields) using a variety of (substituted) isatins and amine-containing compounds (thio/semicarbazide and metformin) as the reaction substrates. The results are depicted in Scheme 4 and Table 4.

**Table 4:** Fe_3_O_4_@PFBA-metformin@SO_3_H-catalyzed reaction of 2-piperazinyl quinoxaline or 2-piperazin-1-ylmethyl-1*H*-benzoimidazole with formaldehyde and various substituted isatin derivatives under optimized reaction conditions

Ligands	Chemical structure		Binding energy (kcal mol^−1^)
(2a)	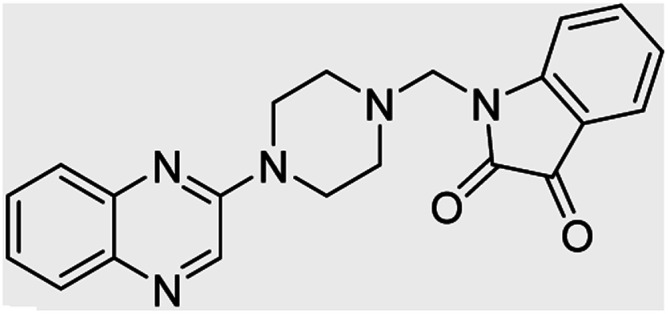	Yield (90%) mp 160–162 °C	−6.52
(2b)	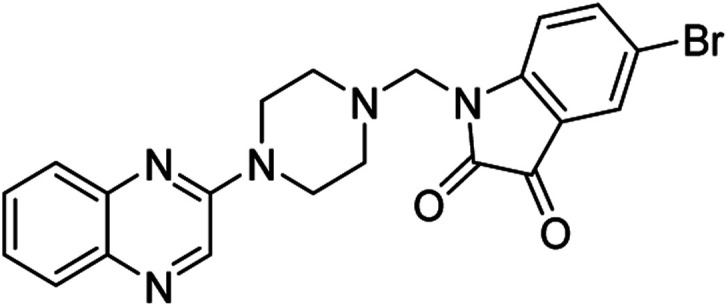	Yield (98%) mp 140–142 °C	−7.31
(2c)	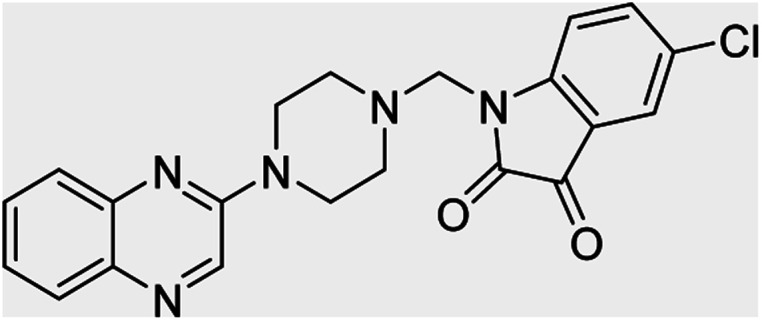	Yield (98%) mp 180–183 °C	−7.07
(2d)	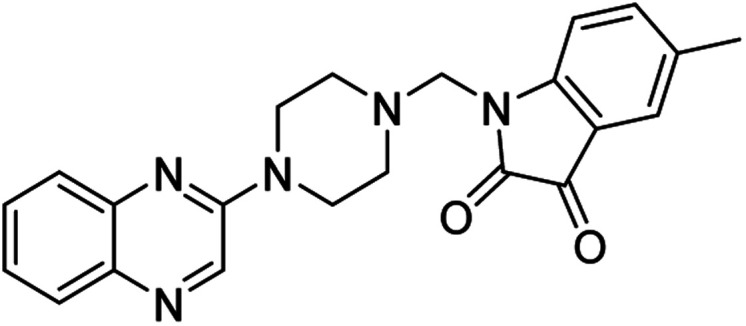	Yield (95%) mp 170–172 °C	−6.57
(3a)	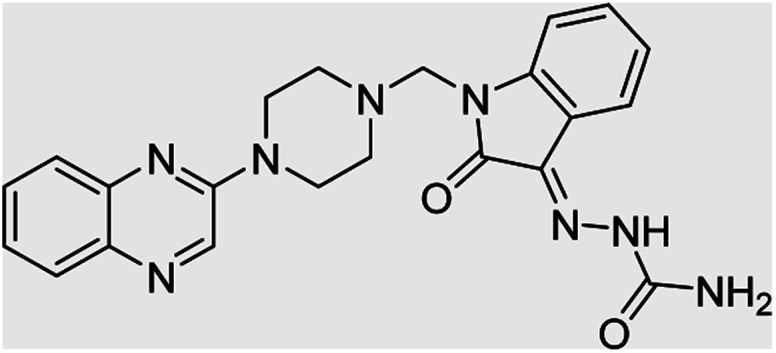	Yield (95%) mp 186–170 °C	−7.47
(3b)	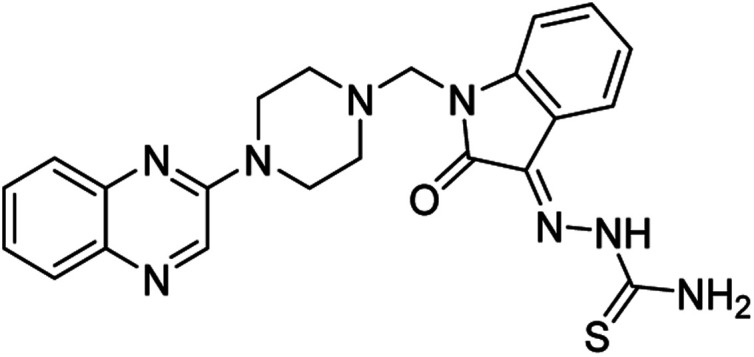	Yield (95%) mp 185–187 °C	−7.79
(3c)	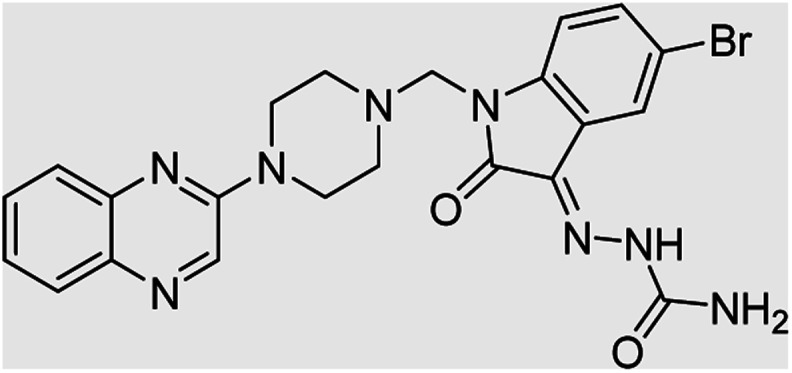	Yield (98%) mp 125–129 °C	−8.01
(3d)	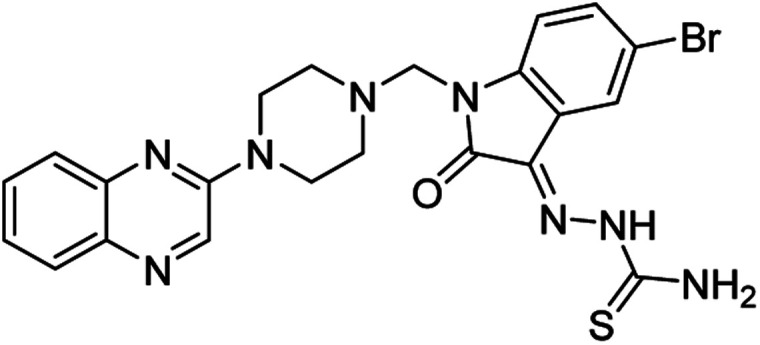	Yield (99%) mp 132–137 °C	−8.31
(3e)	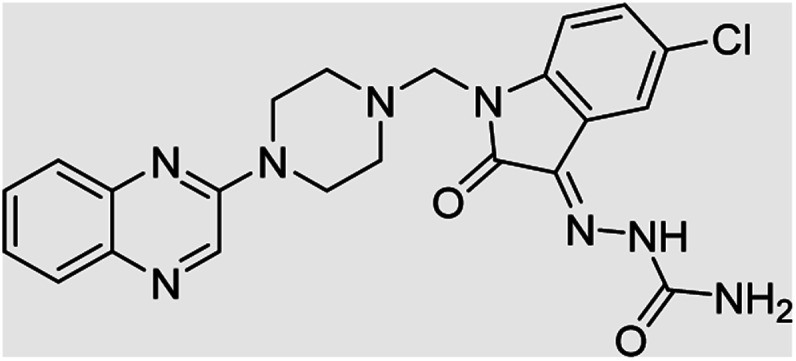	Yield (98%) mp 177–179 °C	−7.60
(3f)	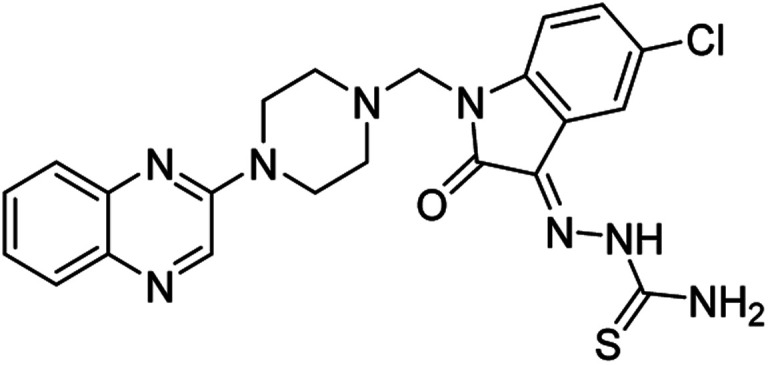	Yield (99%) mp 179–183 °C	−8.14
(3g)	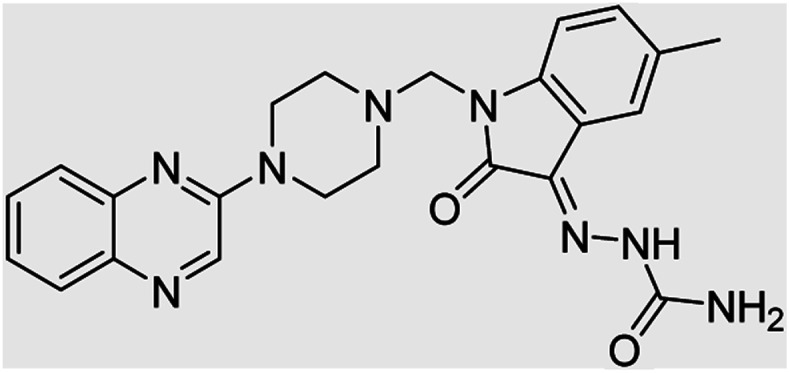	Yield (95%) mp 192–195 °C	−7.11
(3h)	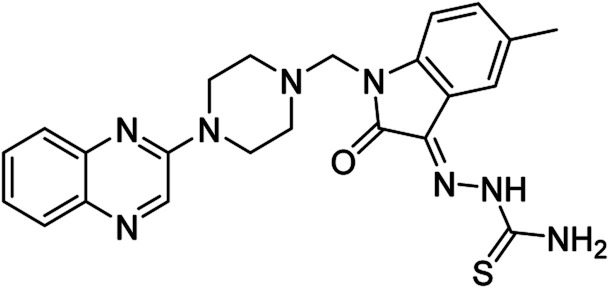	Yield (97%) mp 163–166 °C	−7.55
(4a)	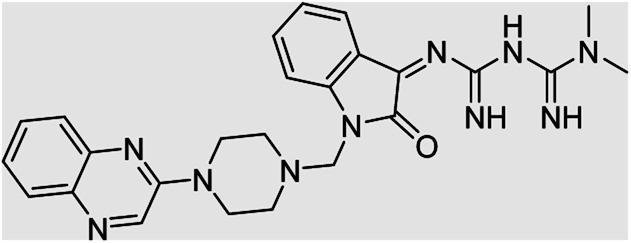	Yield (90%) mp 90–95 °C	−8.53
(4b)	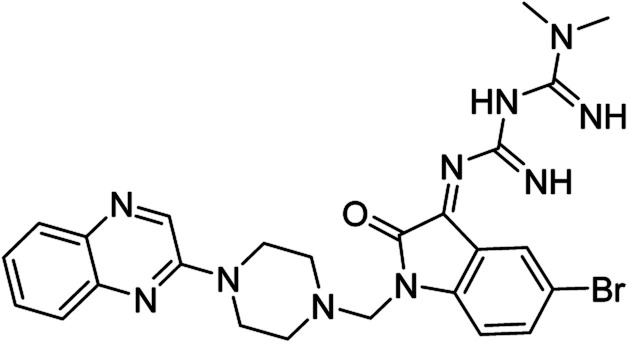	Yield (95%) mp 108–110 °C	−8.61
(4c)	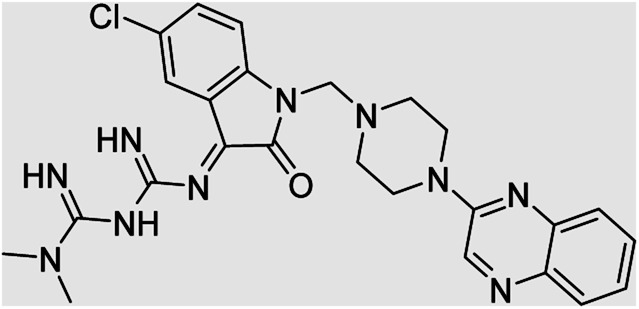	Yield (95%) mp 110–112 °C	−9.22
(4d)	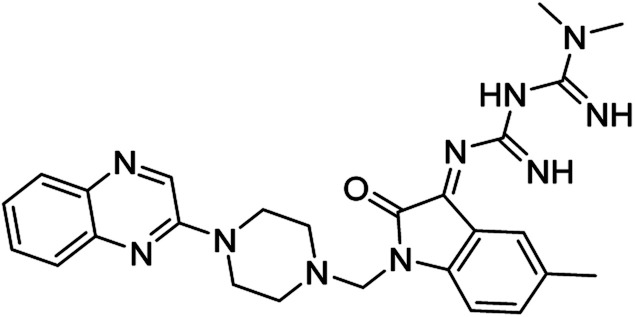	Yield (90%) mp 118–120 °C	−8.61
Imatinib (STI-571)	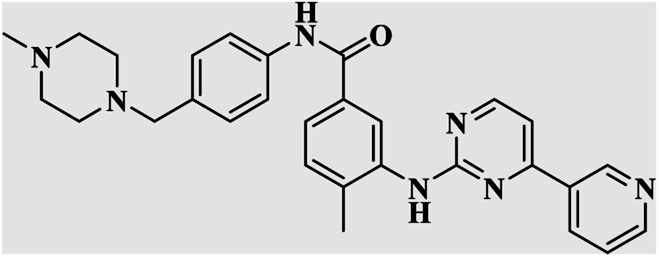		−10.56

## Colorimetric MTT cell viability assay: anti-proliferation effects and selective toxicity of the tested 2-piperazinyl quinoxaline derivatives *vs.* imatinib

**Fig. 3:**
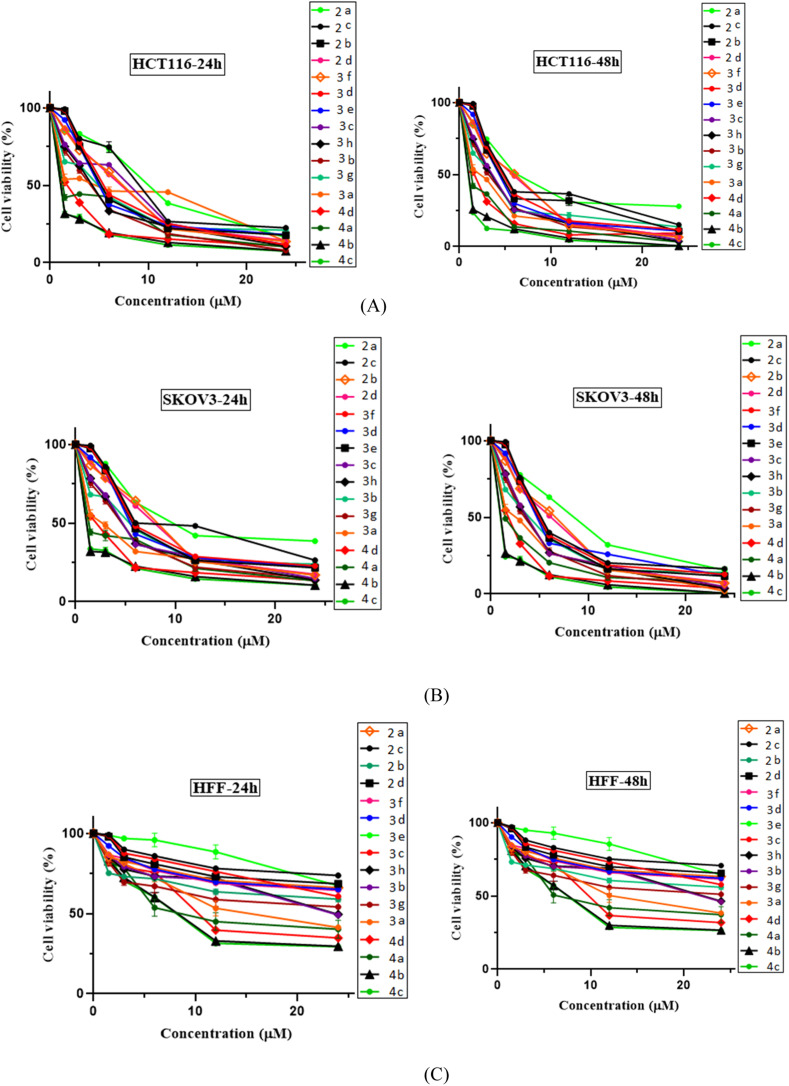
Cell viability (%) studies, as evaluated by MTT assay, of sixteen synthetic final compounds (2a–2d, 3a–3h, and 4a–4d) in cancer and normal cells, HCT116 (A), SKOV3 (B), and HFF (C) respectively, after 24 and 48 h of exposure at various concentrations 0.15, 0.3, 0.6, 1.2 and 2.4 μM. As it can be seen, the concentration values are inserted as multiples of 10 in the related charts.

All the evaluated *N*-Mannich bases (2a–2d, 3a–3h and 4a–4d) were investigated for anti-proliferative activity against two cancer cell lines, HCT116 (human colon cancer) and KOV3 (human ovarian carcinoma). HFF (human foreskin fibroblasts) normal cells were also tested to determine the selectivity of their cytotoxic effects in killing cancer over normal cells. Cells were treated with each compound at five increasing concentrations (1.5, 3, 6, 12, 24 μg ml^−1^), and imatinib was used as our standard positive control in the biological evaluation process. After 24 and 48 h of incubation, cell viability was measured by the [3-(4,5-dimethylthiazol-2-yl)-2,5-diphenyltetrazolium bromide] (MTT) colorimetric assay.^34^ At the end of each incubation period, the IC50 value and cell viability for each *N*-Mannich base were calculated in different concentrations (0.15–2.4 μM) and determined graphically as established in Fig. 3. This informative data provides a measure of efficacy and potency of these compounds. All the expressed data are representative as mean ± SD of at least three independent experiments with similar results. A DMSO equivalent control (*n* = 2 ± SEM) was also included for each cell line tested. Standard graph was plotted by taking concentration of the drug in *X* axis and relative cell viability in *Y* axis (cell viability (%) = mean OD/control OD × 100%) in Graph Pad Prism 8.

The obtained biological results which are depicted in (Fig. 3 and Table 1), revealed that all the tested *N*-Mannich bases had significant concentration and time dependent cytotoxic effect on the both evaluated cancer cell lines compared to imatinib as positive standard, but did not exert any remarkable effect on HFF cell line. According to the observed experimental results, it was demonstrated that almost all of the screened compounds were more active than imatinib (IC50 > 10). The average IC50 of 3.37 μM for HTC116 and 3.64 μM for SCOV3 cells were determined considering the viability of cell lines in 48 h of exposure (Fig. 3, parts (A) and (B)). High selectivity index (SI) for each compound (Table 5, columns 3 and 5) suggests that these *N*-Mannich bases are more toxic to cancer cells than normal cells. Therefore, these newly proposed hybrid compounds can be considered as selective cytotoxic agents.

**Table 5:** Cytotoxic activity of final products (2a–2d, 3a–3h and 4a–4d), IC50 after 48 h incubation

Test compounds	IC_50_ values in (μM)				
	HCT116	SI^a^	SKOV3	SI	HFF
2a	7.031	11.8	7.670	10.8	83.60
2b	5.121	11.3	5.428	10.7	58.13
2c	6.062	13.5	6.491	12.6	82.05
2d	5.001	11.6	5.296	10.9	58.05
3a	1.987	6.8	2.207	6.1	13.51
3b	3.034	8.1	3.384	7.2	24.61
3c	3.348	10.7	3.515	10.2	35.92
3d	4.852	10.2	5.030	9.8	49.57
3e	4.305	8.7	4.962	7.5	37.62
3f	4.922	11.6	5.148	11.1	57.11
3g	2.918	7.8	3.250	7.0	22.82
3h	3.229	8.2	3.464	7.3	26.59
4a	1.231	7.4	1.509	6.0	9.111
4b	0.4581	15.4	0.4877	14.4	6.945
4c	0.3921	17.46	0.4401	15.47	6.813
4d	1.545	6.5	1.716	5.8	10.01
Imatinib	>10	—	> 10	—	>10

a Selectivity index (SI) = IC50 of pure compound in a normal cell line/IC50 of the same pure compound in cancer cell line, where IC50 is the concentration required to inhibit 50% of the cell population.

In this research, all the tested 2-piperazynyl quinoxaline hybrid derivatives induced significant cell cytotoxicity against the selected cancer cells in a time and concentration dependent manner. Although all of the evaluated compounds revealed higher anti-proliferative activity (IC50 < 10 after 48 h incubation) comparing to imatinib with IC50 > 10, in molecular dynamic simulations, imatinib showed higher affinity and free energy scores. Apart from the ATP binding pocket of the catalytic domain and kinase activity,^35^ the less noticed aspect of c-Kit receptor is its apoptosis induction in various tumor cell lines if not engaged to its endogen ligand stem cell factor (SCF). Indeed, in cancers showing SCF expression, inhibiting the kinase activity may not be sufficient to cell death induction in tumor cells.^36–39^ In the biological evaluation section of this research, HCT116 cells were chosen as c-Kit positive and SCF-expressing malignant cells. Therefore, the evaluated *N*-Mannich bases which were designed to be efficient tyrosine kinase inhibitors can be considered as efficient multi-target antitumor compounds to develop novel anti-cancer chemotherapeutics.

## Structure activity relationship

All the newly synthesized sixteen *N*-Mannich bases (2a–2d, 3a–3h, and 4a–4d) showed IC50 < 10 μM on the tested cancer cell lines. In general, the *N*-Mannich bases that were generated by the hybridization of the 2-piperazinyl quinoxaline core with isatin-based Schiff bases of metformin and/or thio/semicarbazones as terminal portion (3a–3h and 4a–4d) were more potent than the molecules generated from combining the 2-piperazinylquinoxaline and (substituted) isatins (2a–2d) as the reported data in Table 1. Among all the tested final products, the metformin tail carrying compounds (4a–4d) revealed the higher cytotoxicity against both tested cancer cell lines. The compounds 4b and 4c which contain halogen-substituted isatin portions, showed the lowest IC50 values (IC50s 0.45 and 0.39 μM in HCT116, and IC50s 0.48 and 0.44 μM in SCOV3 cells respectively, after 48 h incubation). Further, these promising compounds were screened against normal cells HFF and the selectivity index (SI) values was also determined. High SI value (>2) of these compounds exhibited their selective toxicity towards cancer cells. After Metformin containing derivatives (4a–4d), the other tail caring compounds (3a–3h) showed the higher cytotoxic activity than tail-free compounds (2a–2d) as it has shown in Table 1 (columns 2 and 4, entries 5–12). Among compounds 3a–3h containing (substituted) isatin-3-thio/semicarbazones, at the first level, the semicarbazone derivatives of isatin and methyl isatin (compounds 3a and 3g) showed higher cytotoxicity than the corresponding thiosemicarbazone derivatives (3b and 3h). At the next level, *N*-Mannich bases containing halogenated isatins which are carrying semicarbazone tail (compounds 3c and 3e) showed higher cytotoxicity than the corresponding thiosemicarbazone derivatives (3d and 3f). Final products 2a–2d showed higher IC50 range than their tail containing analogs (5.00–7.67 μM); but these compounds still can have considered efficient anticancer agents with high SI values, which make them suitable candidates for further investigations and development procedures. These results confirmed the essential role of the hetero aliphatic tail portion (isatin-based Schiff bases of metformin and/or thio/semicarbazones) in the final products for cytotoxicity, especially metformin portion which afforded a significant increase in potency. It also revealed that in tail containing compounds, there is no significant difference between cytotoxicity of the final products with Cl and Br at the 5-position of isatin segment.

## Molecular dynamics (MD) simulations on the human c-Kit tyrosine kinase (stem cell factor (SCF)) receptor

### Molecular docking

**Fig. 4:**
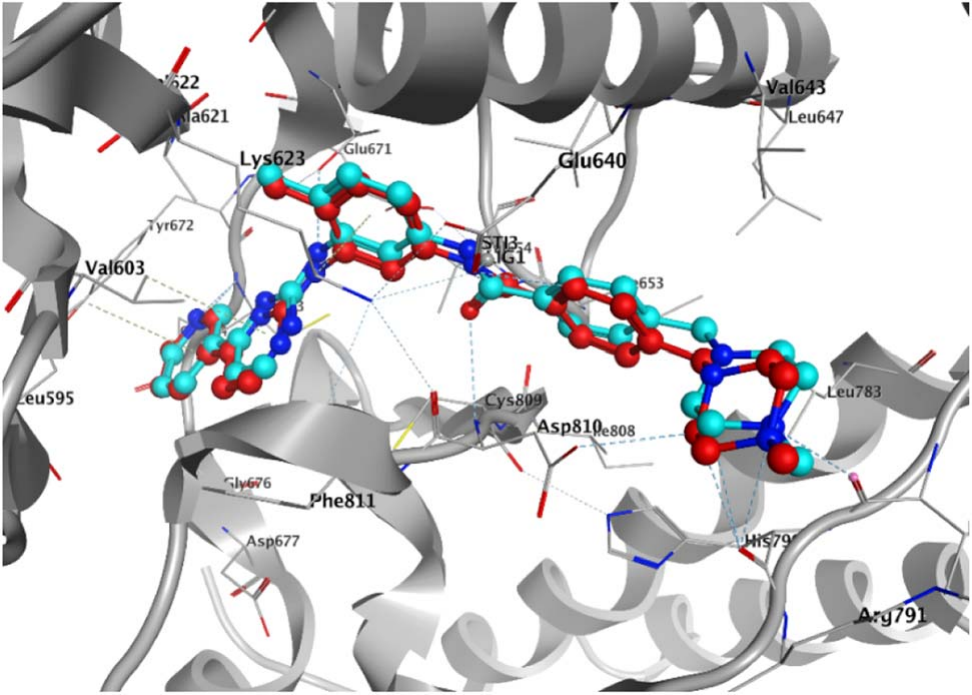
Superposition of the co-crystal structure of the 1T46 ligand–protein complex (red) with the lowest energy conformation (cyan) obtained from docking simulations, which defines its predicted structure.

To elucidate the binding modes of the tested compounds and probe the molecular mechanism of the anti-proliferative activity of these hybrid *N*-Mannich bases, it is inevitable to find out the binding mode of the evaluated ligands at the active site of specific targets which have been selected based on the literature review and the structure–activity-relationship. In this context, molecular docking was used as the initial step of MD simulations.^40^ The docking has been performed on PDB code: 1T46 (c-Kit tyrosine kinase in complex with imatinib (STI-571)). As shown in the Fig. 4, there is a good match between the LeDock result and the crystallographic structure. Based on such model, the pharmacophore included two main hydrophobic subunits and two main hydrogen bonds that hold the compounds firmly in the enzymatic pocket.^41^ According to the Fig. 4, STI-571 which is the control compound in our study, exhibits the most binding energy of −10.56 kcal mol^−1^ forming H-bond interactions with Cys673 and Glu640. After ensuring the performance of the program, all the investigated compounds were docked on the active site of the receptor. According to the resulted data, the binding energies for 3a, 3d and 4c were −7.47, −8.31 and −9.22 kcal mol^−1^, respectively. Unlike the 3a, in the 4c and 3d compounds, the quinoxaline ring is responsible for the stabilizing the ligands by making H-bond with residues Asp677 and Asp810 in the second hydrophobic sub-site of the enzyme. Moreover, the hydrazine carboxamide moiety of 4c participates in H-bond interactions with Asp677, Arg796 and Arg815 while in 3d compound the hydrazine carbothioamide moiety making H-bond with Ala636. The detailed docking results (2D structures of binding mode prediction and binding energies) are introduced in ESI (Fig. S2 and Table S1).†

## Molecular dynamics analysis

**Fig. 5:**
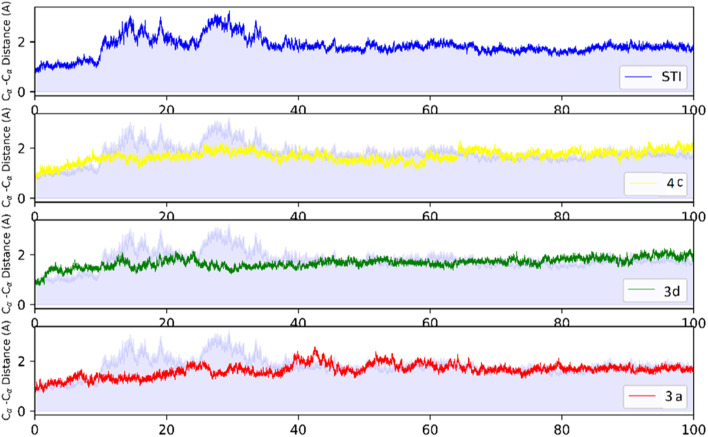
RMSD profile of c-Kit with STI-571, 4c, 3d, and 3a by doing least-square fit to the backbone of starting structure.

MD analysis was carried out to examine the molecular interaction effectiveness of STI-571, 4c, 3d and 3a with active site of c-Kit. In the MD simulation, the dynamic of c-Kit bound to the ligands is a reflection of its functional motion. Root mean square deviation (RMSD) of Cα atoms of protein backbone was monitored throughout 100 ns simulation and shown in Fig. 5.

**Fig. 6:**
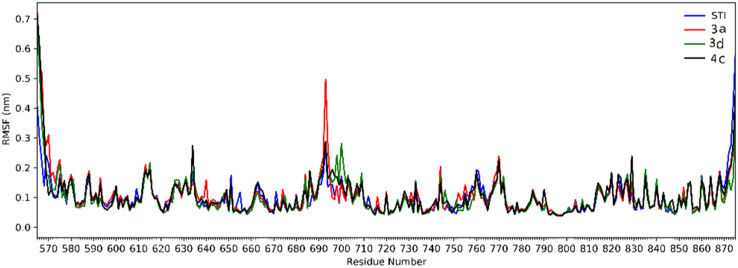
The RMSF of all molecular dynamics systems throughout 35 ns of simulation as a function of protein residue number.

Analysis of RMSD showed that all of the complexes begin to relax from 65 ns. The root mean square fluctuation (RMSF) is defined as the fluctuation of every single atom about its average position. Atomic fluctuations of the Cα atoms of protein were calculated from the last 35 ns and depicted in Fig. 6.

**Fig. 7:**
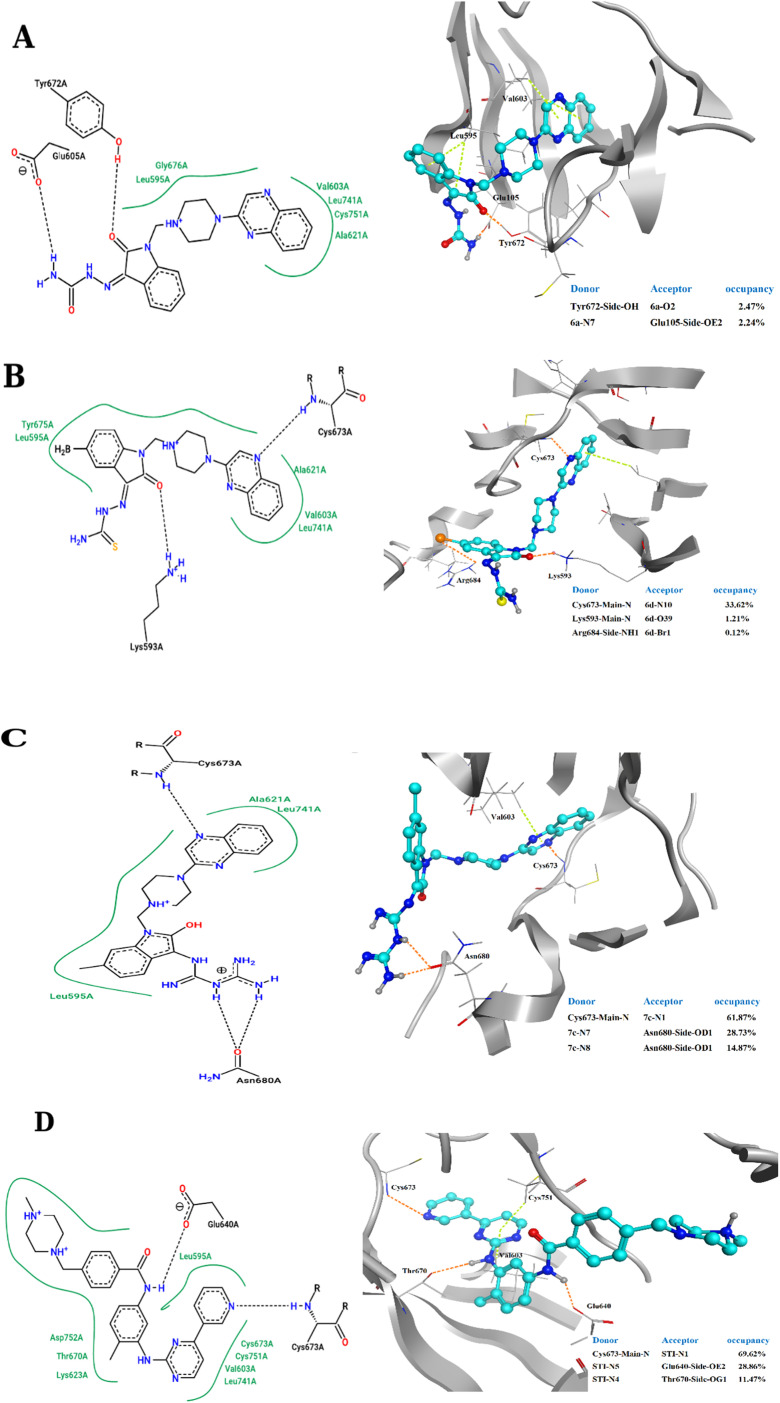
2D representation of representative structures of (A) 3a, (B) 3d, (C) 4c and (D) STI-571 bound to the c-Kit enzyme, obtained from cluster analysis in GROMACS. Hydrophobic interaction depicted as a green solid line whereas the H-bond illusterd as black dash lines. H-bond interactions as represented in 3D structures for (A) 3a, (B) 3d, (C) 4c and (D) STI-571 bound to the c-Kit enzyme. The hydrogen bonds and the π–π stacking interactions shown as orange dash lines and light green dash lines, respectively. Donor and acceptor atom involved in the H-bonds with high occupancies summarized at the corner of each ligand representations.

The 2D representations of representative structures are depicted in Fig. 7. Compared to docking studies, these representative structures contain much more useful information on how compounds are placed on the active site of the protein. As shown in Fig. 7, it seems that two important factors pertaining to the quinoxaline scaffold will play a central role in the placement of ligands in the active site of the enzyme: (i) the presence of hydrogen bonding between the nitrogen atoms in this group with Cys673 and (ii) creation of a hydrophobic cavity is made up of residues Ala621, Leu741 and Val603, which results in the better stability of the quinoxaline scaffold in the active site. However, compound 3a, despite having the latter factor, showing less stability due to the lack of H-bond with Cys673. The energy decomposition analysis is described in ESI (Fig. S3).†

The binding affinity of chosen ligands was studied on the basis of binding free energy estimated using the MM-PBSA method. Table 6 provides the details of contribution terms in the binding free energies for the inhibitors defined in eqn (1)–(4). As can be inferred from Table 6, all energy components order is as follows: STI-571 > 4c > 3d > 3a. It seems that van der Waals (VDW), electrostatics and the solvent-accessible surface area (SASA) energy strongly favours the stability of STI-571, 4c and 3d in the active site of the enzyme. The favourable interactions of VDW can be attributed to the presence of π-stacking interactions between Val603 and quinoxaline rings as shown in Fig. 7. Detailed explanation about the hydrogen bond analysis, is described in ESI (Fig. S4).†

**Table 6:** Details of contribution terms in the binding free energies for the evaluated *N*-Mannich bases

Types of interactions	STI-571	4c	3d	3a
Van der Waals	−320.89^a^ ± 12.87	−177.36 ± 47.85	−156.54 ± 94.93	−21.00 ± 60.02
Electrostatic	−156.68 ± 24.73	−34.64 ± 16.88	−18.20 ± 16.76	−6.19 ± 18.81
Polar salvation	298.30 ± 23.95	134.71 ± 33.17	112.17 ± 61.41	22.83 ± 93.32
SASA energy	−26.89 ± 0.87	−18.28 ± 5.01	−14.94 ± 8.80	−1.88 ± 6.41
Binding energy	−206.16 ± 22.71	−95.56 ± 40.80	−77.52 ± 71.93	−6.24 ± 77.75

a All energies in the table are in kJ mol^−1^.

## Molecular docking (MD) analysis on human P-glycoprotein transporter

To understand the other possible molecular basis of the anti-proliferative properties of the tested *N*-Mannich bases, among the most active compounds (series B and C), the best compounds 4c and 3d were subjected to molecular docking with five different PDB IDs of P-glycoproteins.^42,43^ Very interestingly, as it can be seen in the Table 3, the values of binding energies of ligand 4c were better than of ligand 3d and which these docking results are consistent with the experimental part of this research. Therefore, the data reported by docking studies show that ligand 4c has a greater tendency to bind to any of the selected proteins than ligand 3d. As shown in the Fig. 7, it was found that in PDB ID: 3G5U, the residues, Thr37, Ala42, Arg40, and Arg47 play an important role in the active site; that in this study, Ala42 and Arg47 are shown as the more favorable sites to dock selected ligands to this structure. The best binding energy related to the docking of each ligand with the selected protein and the most important residues in the active site of each selected PDB are reported in Table 7.

**Table 7:** Docking results: free binding energies and the most important involved residues for 3d and 4c ligands interactions with the selected PDBs

PDB IDs	Ligands and free binding energies		The most important involved residues
	3d – free binding energies (kcal mol^−1^)	4c – free binding energies (kcal mol^−1^)	
3G5U	−7.46	−8.48^a^	Thr37, Ala42, Arg40, and Arg47
3G60	−7.28	−7.61	Phe728, Val978, Tyr949 and Tyr303
3G61	−6.76	−7.47	Val978, ASN717 and Ala981
4M1M	−7.37	−8.16	Phe724, Phe332 and Met982
6Q81	−6.70	−7.95	Ser403, Glu402, and Cys427

a The lowest free binding energy in the molecular docking simulation study of the selected ligands 3d and 4c against five introduced PDB IDs for transporters P-glycoprotein: 3G5U, 3G60, 3G61, 4M1M and 6Q81.

**Fig. 8:**
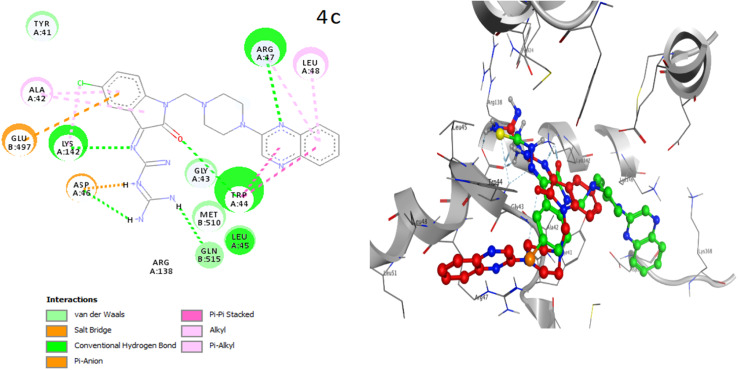
As the best example, important interactions of ligand 4c ligand with the receptor 3G5U as docking results are presented.

The placement of the ligand 4c in the active site of proteins and the creation of effective interactions with important residues related to each structure, well show the results obtained from molecular docking and its superiority over the ligand 3d in Fig. 8. The complete docking results of 3d and 4c ligands with PDB IDs of 3G5U, 3G60, 3G61, 4M1M and 6Q81 including the location of the selected ligands in the active site and their important interactions are presented in ESI (Fig. S5: parts A–E†). All the tested compounds can be categorized in three groups of different structural patterns. Compound 4c with haloisatin-based Schiff base of Metformin and 3d incorporating halosatin-based thiosemicarbazone exhibited high docking scores with p-glycoprotein which reflect the effective interactions.

## Conclusion

In the current study, Novel an efficient acidic nanocatalyst (FeAl_2_O_4_@PTMS-sulfaguanidine-SA MNPs) was developed for one-pot multicomponent cyclocondensation and Mannich reactions with magnetic properties, which can reduce the problems of using mineral or traditional procedure and homogeneous organic catalysts. FeAl_2_O_4_@PTMS-sulfaguanidine-SA MNPs is a green, environmentally friendly, nontoxic, economical, and easy workup nanocatalyst with magnetic properties, which the latter guarantees its easy recovery by a simple magnet. This newly synthesized magnetic nanocatalyst can be reused several times with no remarkable drop in the catalytic activity. In this work, the 2-piperazinyl quinoxaline derivatives containing isatin-based thio/semicarbazones or Schiff bases of Metformin have been synthesized in the presence of the prepared nanocatalyst and then evaluated for anticancer activity. According to the obtained results from *in vitro* investigations and calculated SI values, all the tested final products specially the most active promising compounds series C (compounds 4a–4d) with average growth inhibition values ≤1 μM, could be regarded as novel anticancer candidates for further studies. The rest of the compounds (series A: compounds 2a–2d and series B: compounds 3a–3h) showed an average growth inhibition value <10 μM at the tested concentration levels (0.15–2.4 μM). Based on the structural design of these hybrid molecules, the 2-piperazynyl quinoxaline core and isatin-based thio/semicarbazone functional scaffold can target RTKs and Pgp transporters in cancer cells. Molecular docking and dynamics simulation studies displayed the efficient interactions between these hybrid molecules with c-Kit RTKs and Pgp transporters. Briefly, the current study demonstrated that the newly synthesized 2-piperazinylquinoxaline derivatives incorporating isatin-based Schiff bases (compounds 4c and 4b) can be employed in future anti-cancer drug design and development studies.

## Abbreviations

BET: Brunauer–Emmett–Teller (surface area determination)
DMSO: Dimethyl Sulfoxide
EDS: Energy Dispersive Spectroscopy
FTIR: Fourier Transform Infrared (characterization of the related group)
IC50: Half Maximal Inhibitory Concentration
MD: Molecular Docking
MNPs: Magnetic Nanoparticles
MTT: 3-(4,5-Dimethylthiazol-2-yl)-2,5-diphenyl-2*H*-tetrazolium bromide
Pgp: P-Glycoprotein
RTKs: Receptor Tyrosine Kinases
SEM: Scanning Electron Microscope (morphology and size distribution)
SI: Selectivity Index
TEM: Transmission Electron Microscopy
TGA: Thermogravimetric Analysis (thermal stability)
VSM: Vibrating Sample Magnetometer

## Conflicts of interest

The authors declare that they have no competing interests.

## Supplementary Material

RA-013-D3RA03305H-s001
