# Supplementary material for: Green synthesis, anti-proliferative evaluation, docking, and MD simulations studies of novel 2-piperazinyl quinoxaline derivatives using hercynite sulfaguanidine-SA as a highly efficient and reusable nanocatalyst
Source: RSC Adv. 2023 Aug 23;13(36):25229–45. doi: 10.1039/d3ra03305h (PMC10445084; doi:10.1039/d3ra03305h)
Supplement: RA-013-D3RA03305H-s001 [file RA-013-D3RA03305H-s001.pdf]

Electronic Supplementary Material (ESI) for RSC Advances.
This journal is © The Royal Society of Chemistry 2023

Supplementary material

Green Synthesis, anti-proliferative evaluation, docking, and MD simulations studies of novel 2-piperazinyl quinoxaline derivatives using Hercynite sulfaguanidine-SA as a highly efficient and reusable nanoparticle

Zohreh Esam[1], Malihe Akhavan[2], Atefeh Mirshafa[3], Ahmadreza Bekhradnia*[2]

[1]Pharmaceutical Sciences Research Center, Student Research Committee, Department of Medicinal Chemistry, Faculty of Pharmacy, Mazandaran University of Medical Sciences, Sari, Iran.

[2] Pharmaceutical Sciences Research Center, Department of Medicinal Chemistry, Mazandaran University of Medical Sciences, Sari, Iran.

[3]Ramsar Campus, Mazandaran University of Medical Sciences, Ramsar, Iran.

Correspondence: Ahmadreza Bekhradnia, Email: a.bekhradnia@gmail.com

1. Supporting results

1.1. chemistry

The mass spectra of these compounds showed a molecular ion peak, which is corresponding to their molecular formula. The IR spectra of all compounds was characterized by the presence of absorption bands at the expected positions as strong stretching (*str*) absorption bands near 1735 cm^{-1} (N-C=O), 1691 cm^{-1} (N-C=S), 1687 cm^{-1} (C=O), 2777 cm^{-1} (Ar-CH), 1491 cm^{-1} (C=N), 1027 cm^{-1} (C–N), 3424 and 3064 cm^{-1} (N-H); along with those associated to the substituents near 693 cm^{-1} (C-Cl) and 757 cm^{-1} (C-Br). NMR spectra were run either in $CDCl_3$ or DMSO. The ^{1}H-NMR spectra of all N-Mannich bases revealed tow multiplets at around 2.69 and 3.25 ppm, corresponding to the piperazine ring hydrogens; and one singlet at around 4.23 ppm, that correspond to the CH_2 bridge in aliphatic region (Figure S1). Apart from the aliphatic region and

different substitutions, ^{1}H-NMR and also ^{13}CNMR spectra of compounds 2a-2d (series A), 3a-3f (series B), and 4a-4c (series C) showed similar patterns due to the same structural patterns; one singlet at 8.48 ppm, corresponding to the 3'-hydrogen; two doublets (J = 8.8 Hz) near 7.63 and 8.06 ppm, that correspond to 5’ and 8' hydrogen respectively; one multiplets at around 7.46 ppm corresponding to 6’ and 7' hydrogens; and three singlets at around 8.59, 8.76, and 9.98 corresponding to C=NH groups and NH moiety respectively. With respect to the ^{13}C-NMR spectrum, tow of azomethine carbons at around 156.50 and 166.88, and at near 167.61 corresponding to non-protonated C=O carbon. More corresponding information can be found in our previously published article [1].

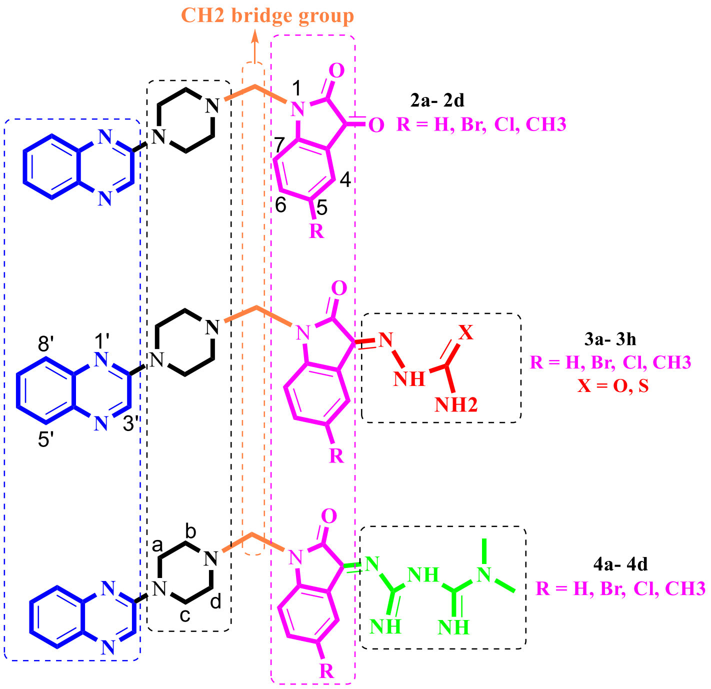

Figure 1. The designed N-Mannich bases from 2-piperazinyl quinoxaline as the core scaffold, attached to the (substituted) isatin, and the tail potion thio/semicarbazone and/or metformine, via a CH_2 bridge derived from formaldehyde.

1.2. Molecular dynamics (MD) simulation (on c-Kit receptor as target)

1.2.1. Molecular Docking

Molecular docking was the first step of this virtual evaluation. It is required to investigate the ability of docking program by reproducing the native binding of a crystallographic ligand [2]. Recently, a comparative study has been done to evaluate this ability for varieties of docking programs [3]. In our work, in order to make sure the ability of the chosen program in reproducing the native binding mode of crystallographic compound, the X-ray crystal structure of c-Kit tyrosine kinase (PDB code: 1T46) in complex with Imatinib (STI-571) was obtained from Brookhaven protein data bank (http://www.rcsb.org/) [4]. The detailed docking results of binding mode prediction for 3a, 3d, and 4c ligands and calculated binding energies are shown in Figure S2 and Table S1.

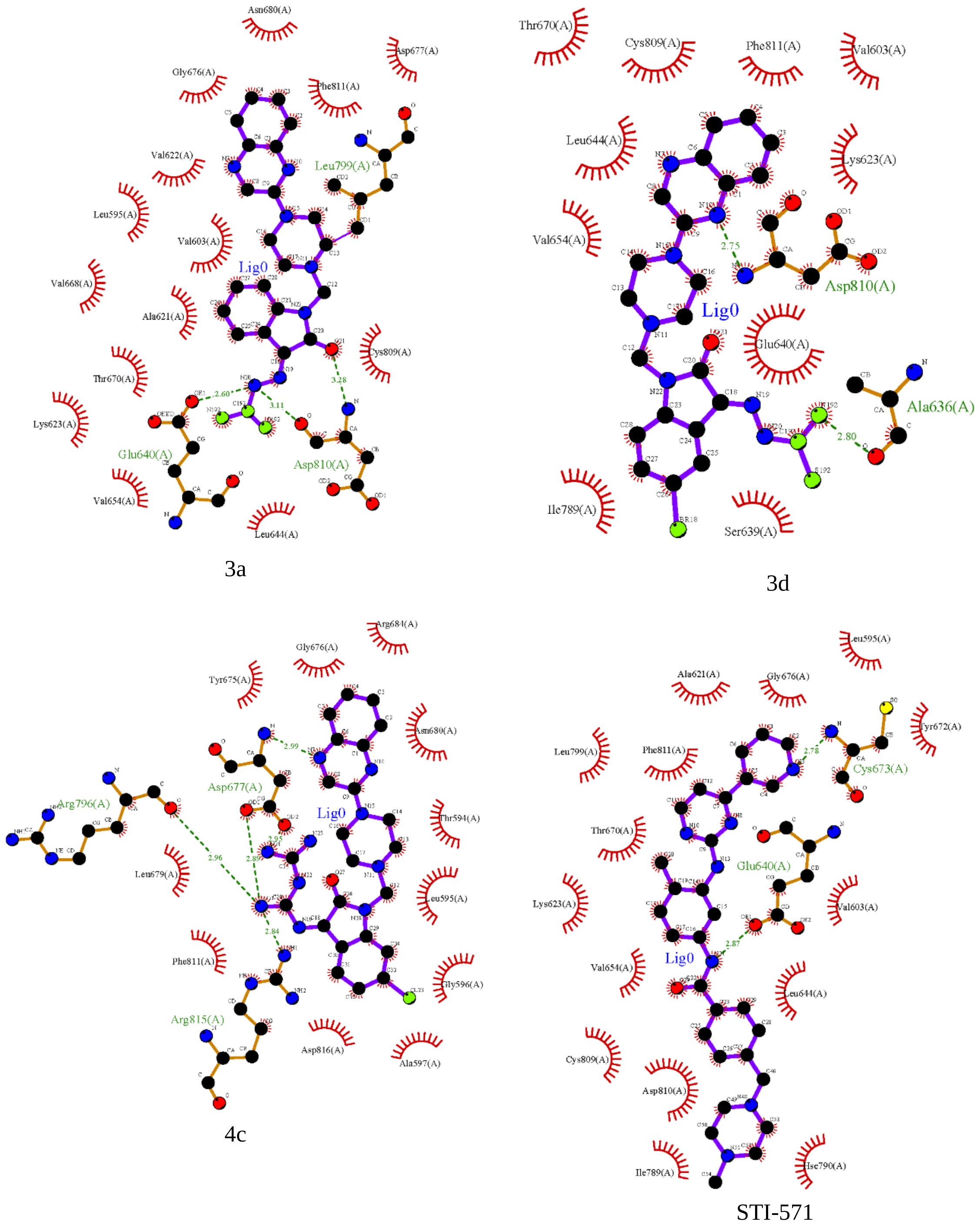

Figure S2. Proposed binding mode for co-crystal ligand STI-571 as well as compounds 4c, 3d, and 3a with c-Kit tyrosine kinase in the docking study. Important residues of the active site of the kinase were Cys673, Glu640 and Asp810 in two hydrophobic pockets. Color code for heteroatoms is as follows: nitrogen: blue, oxygen: red, sulfur: yellow, and chlorine: green (PDB deposition code 1T46).

Table S1. Representative ligands and their docking binding energy.

Ligands	Chemical Structure	Binding energy (Kcal/mol)
1-((4-(quinoxalin-2-yl) piperazin-1-yl) methyl) indoline-2,3-dione (2a)	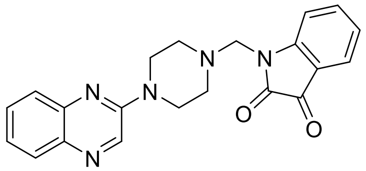	**-6.52**
5-bromo-1-((4-(quinoxalin-2-yl) piperazin-1-yl) methyl) indoline-2,3-dione (2b)	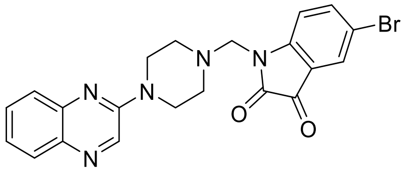	**-7.31**
5-chloro-1-((4-(quinoxalin-2-yl) piperazin-1-yl) methyl) indoline-2,3-dione (2c)	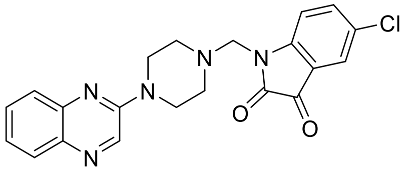	**-7.07**
5-methyl-1-((4-(quinoxalin-2-yl) piperazin-1-yl) methyl) indoline-2,3-dione (2d)	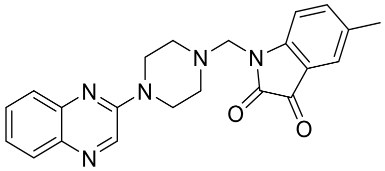	**-6.57**
2-(2-oxo-1-((4-(quinoxalin-2-yl) piperazin-1-yl) methyl) indolin-3-ylidene) hydrazine-1-carboxamide (3a)	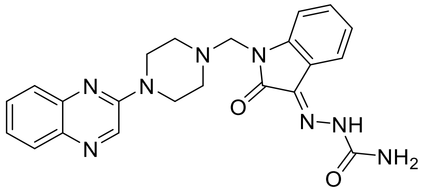	**-7.47**
2-(2-oxo-1-((4-(quinoxalin-2-yl) piperazin-1-yl) methyl) indolin-3-ylidene) hydrazine-1-carbothioamide (3b)	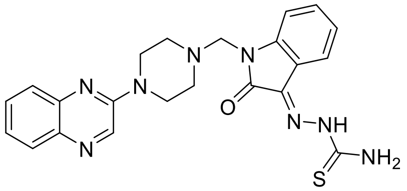	**-7.79**
2-(5-bromo-2-oxo-1-((4-(quinoxalin-2-yl) piperazin-1-yl) methyl) indolin-3-ylidene) hydrazine-1-carboxamide (3c)	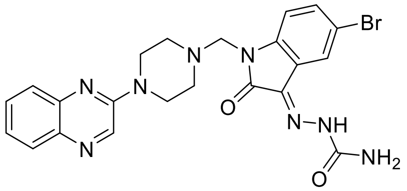	**-8.01**
2-(5-bromo-2-oxo-1-((4-(quinoxalin-2-yl) piperazin-1-yl) methyl) indolin-3-ylidene) hydrazine-1-carbothioamide (3d)	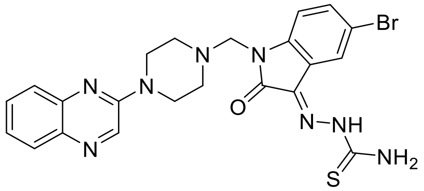	**-8.31**

2-(5-chloro-2-oxo-1-((4-(quinoxalin-2-yl) piperazin-1-yl) methyl) indolin-3-ylidene) hydrazine-1-carboxamide (3e)	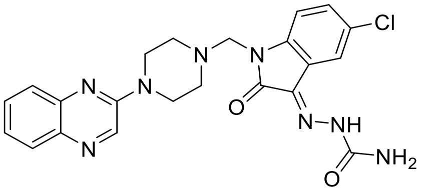	**-7.60**
2-(5-chloro-2-oxo-1-((4-(quinoxalin-2-yl) piperazin-1-yl) methyl) indolin-3-ylidene) hydrazine-1-carbothioamide (3f)	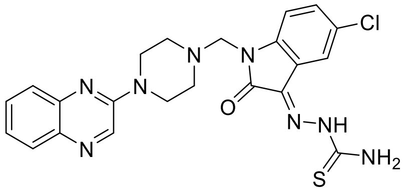	**-8.14**
2-(5-methyl-2-oxo-1-((4-(quinoxalin-2-yl) piperazin-1-yl) methyl) indolin-3-ylidene)hydrazine-1-carboxamide (3g)	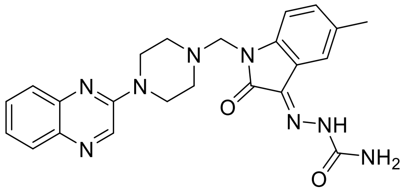	**-7.11**
2-(5-methyl-2-oxo-1-((4-(quinoxalin-2-yl) piperazin-1-yl) methyl) indolin-3-ylidene) hydrazine-1-carbothioamide (3h)	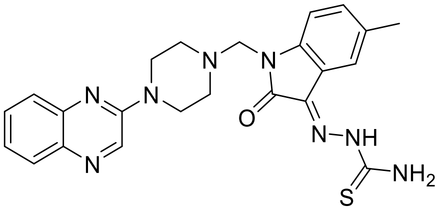	**-7.55**
N'-[4-(quinoxaline-2-yl)-piperazine-1-yl]methyl-1-H-indole,2,3-dion-3- metformin (4a)	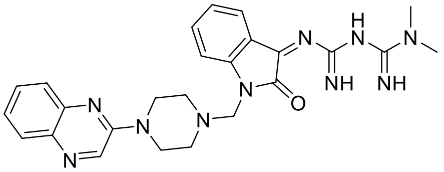	**-8.53**
N'-[4-(quinoxaline-2-yl)-piperazine-1-yl]methyl-5-Bromo-1-H-indole,2,3-dion-3- metformin (4b)	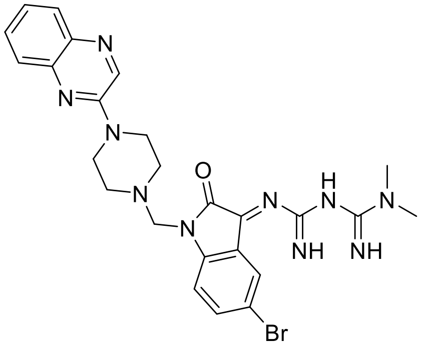	**-8.61**
N'-[4-(quinoxaline-2-yl)-piperazine-1-yl]methyl-5-chloro-1-H-indole,2,3-dion-3- metformin (4c)	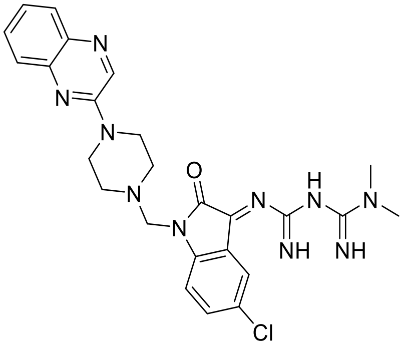	**-9.22**

N’-[4-(quinoxaline-2-yl)-piperazine-1-yl]methyl-5-methyl-1-H-indole,2,3-dion-3- metformin (4d)	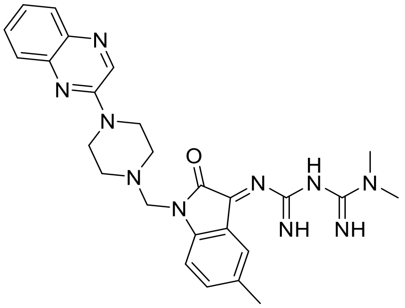	**-8.61**
Imatinib **(STI-571)**	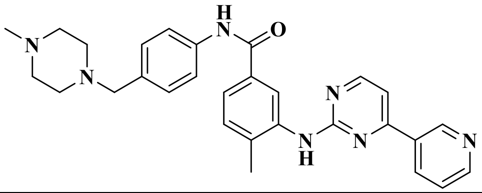	**-10.56**

1.2.2. Molecular Dynamics

Clustering analysis is commonly used to determine protein conformations by reducing the size of the problem of conformational analysis by dividing the conformations sampled into separate groups [5]. The midpoint structure is commonly used as the best estimation of the conformations contained within a specific cluster to ensure that the cluster representative is a physically reasonable structure. Conformational clustering was performed on the molecular dynamics trajectory of the aforementioned protein-ligand complexes in order to intelligently select representative conformations for use in further analysis. Clustering was performed using Cα backbone atoms, least-squares alignment, and the Gromos algorithm [6] by g_cluster module as implemented in Gromacs 2020.1. However, we considered the centre structures of most populated and hence more stable clusters of each complex as representatives of binding mode conformations.

1.2.2.1. Energy decomposition analysis

Another useful information that the g_mmpbsa tool can provide is to determine the relative contribution of each residue to the binding free energy of c-Kit/legend complexes. The results have

been shown in Figure S4. According to Figure S4-D, In the case of STI-571, decomposition analysis reveals that the Asp752, Asp579, Glu633, Glu635, Glu640, Arg733 Asp752, Asp793 have the considerable contribution to the binding energy of this ligand. Amino acids which favour the most in the binding energy of 4c are Leu595, Val603, Tyr672, and Leu741as shown in the Figure S4-C. The Leu595, Val603, Glu605, Tyr672, Tyr675, Gly676 and leu741 favour the binding energy of 2d while Leu595, Val603, Glu635, Leu741, Asp758, Glu840 are responsible in favouring the binding energy of 3a inhibitor as exhibited in Figure S4, parts B and A, respectively. It is obvious that the amino acid contributions, for instance, the Leu595 are not equal on the favouring the binding energy of 4c, 3d and 3a.

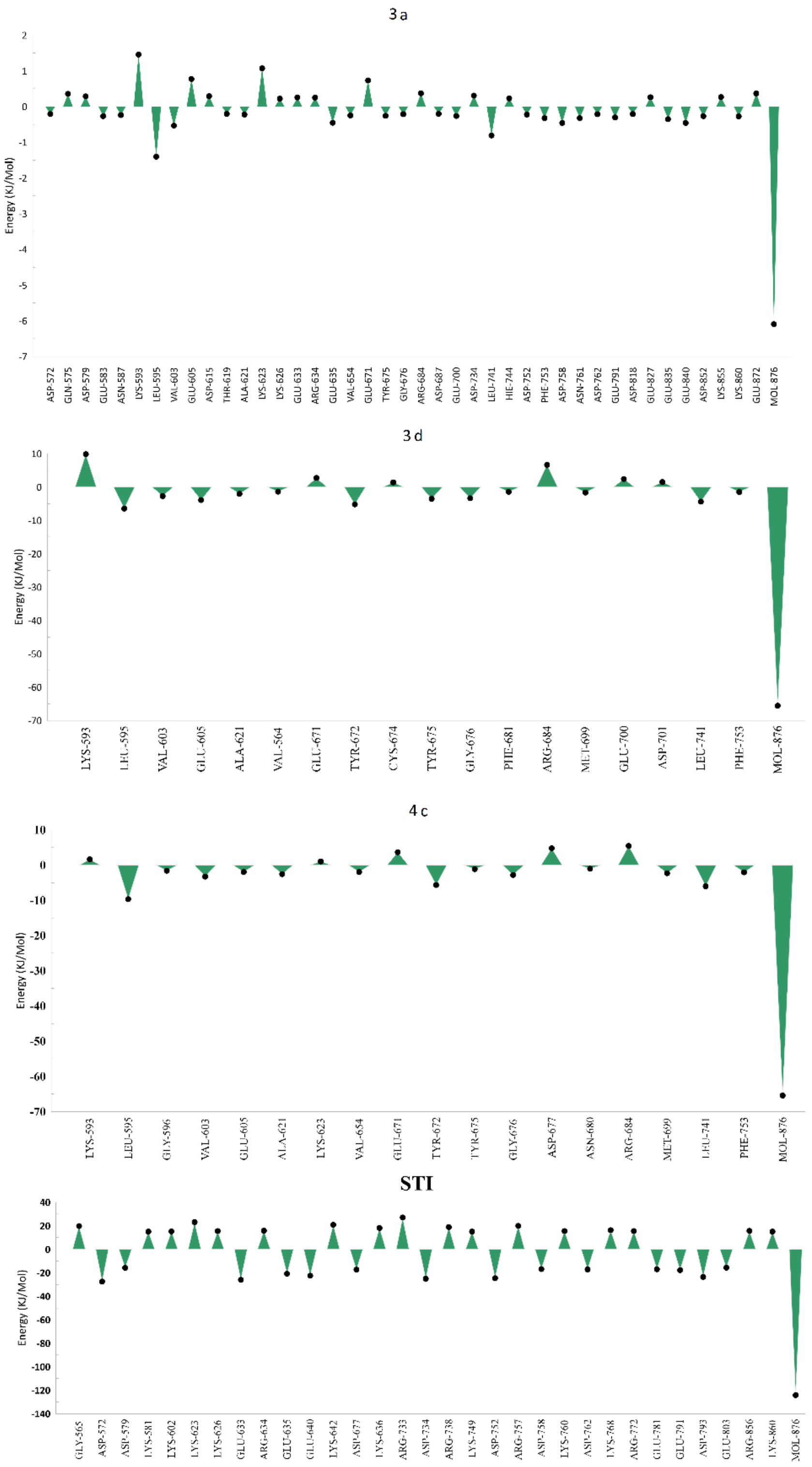

Figure S3. Per-residue binding energy decomposition of predicted ligand/c-Kit complexes. The energy contribution larger than ±5 kcal/mol except for the 3a compound is displayed. The green con shows the residues with absolute binding free energy.

1.2.2.3. Hydrogen bond analysis

To determine the stability of hydrogen bonds between STI-571, 4c, 3d and 3a with the c-Kit, MD analysis of ligand-enzyme complex stability was monitored during the last 35 ns of trajectory period. Hydrogen bond profiles between the selected ligands and the c-kit enzyme were calculated using the g_hbond utility of GROMACS as described in details in our previous study [7]. The threshold for H-bond forming was 3.5 angstrom and with an angle of 30. The results of this analysis has been shown in Figure S3. The analysis disclosed that the stable hydrogen bond between Cys673 and one of the nitrogen atoms in quinoxaline moiety has an astonishing impact on the order of inhibition activities of the aforementioned ligands. The percent of occupancy shows the number of frames in which the particular H-bond has been monitored out of all processed frames. As results show, the STI-571 is formed the more stable hydrogen bond between its N1 atom and the main chain nitrogen atom of Cys673 with an occupancy of 69.62% (Figure 9-D). The next in this order is 4c compound which forms the same hydrogen bond with occupancy a percentage of 61.87% (Figure 9-C) and 2d with percentage of 33.62% ranks afterward (Figure S3-B). The hydrogen bond analysis can also promise the emergence of another pharmacophore in the desired compounds. One can define this pharmacophore as a hydrogen bond, as it is formed by OE2 atom of Glu640 or OD1 atom of Asn680 with corresponding atoms in STI-571 and 4c, respectively. It seems that the difference between the activity of 4c and 2d is related to this pharmacophore. Therefore, the 4c with Hydrazine carboxamide group can make this interaction with two N7 and N8 nitrogen atom as reflected in the occupancy percentage of 28 and 14% in hydrogen bond analysis.

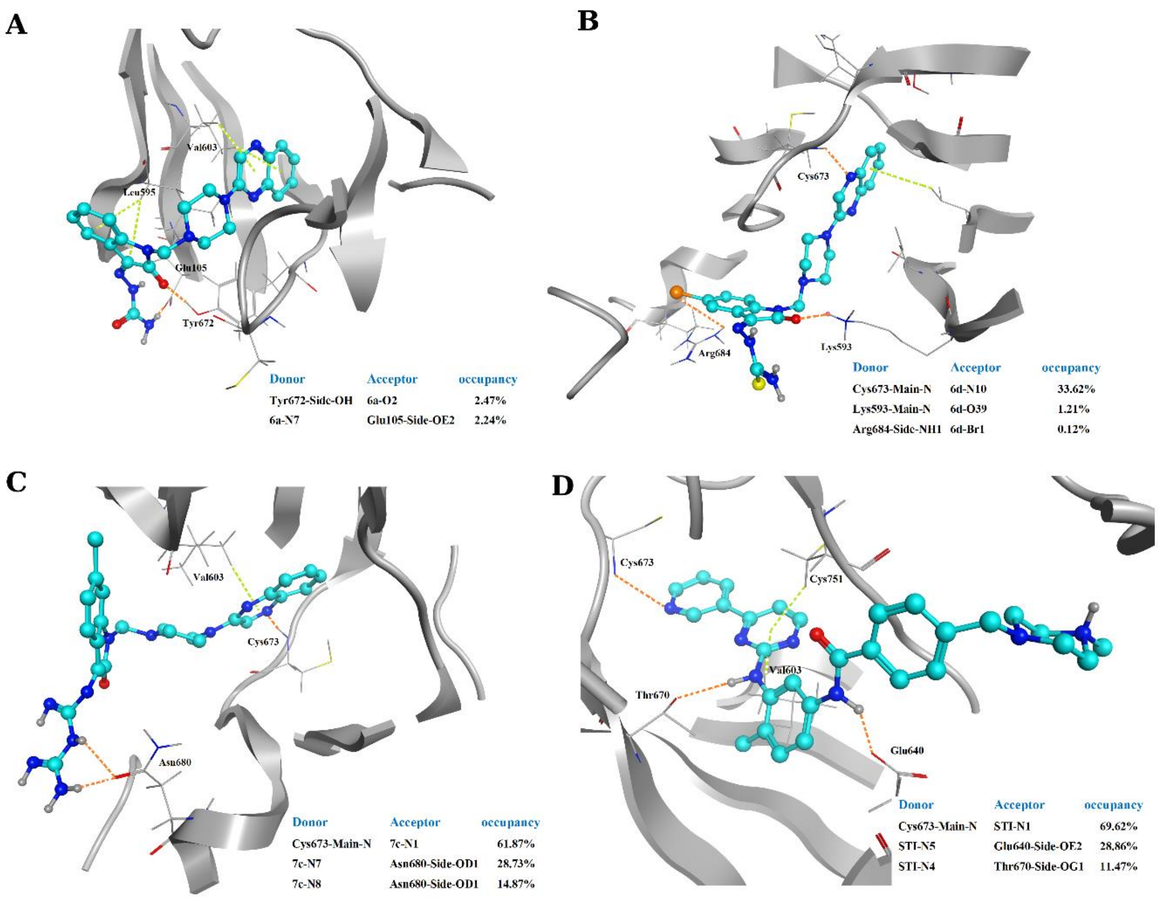

Figure S4. The hydrogen bonds and the π-π stacking interactions shown as orange dash lines and light green dash lines, respectively. Donor and acceptor atom involved in the H-bonds with high occupancies summarized at the corner of each ligand representations.

1.3. Molecular docking analysis (on P-glycoprotein transporter as target)

The complete docking results of 3d and 4c ligands with PDB IDs of 3G5U, 3G60, 3G61, 4M1M and 6Q81 including the location of the selected ligands in the active site and their important interactions are presented in Figure S4.

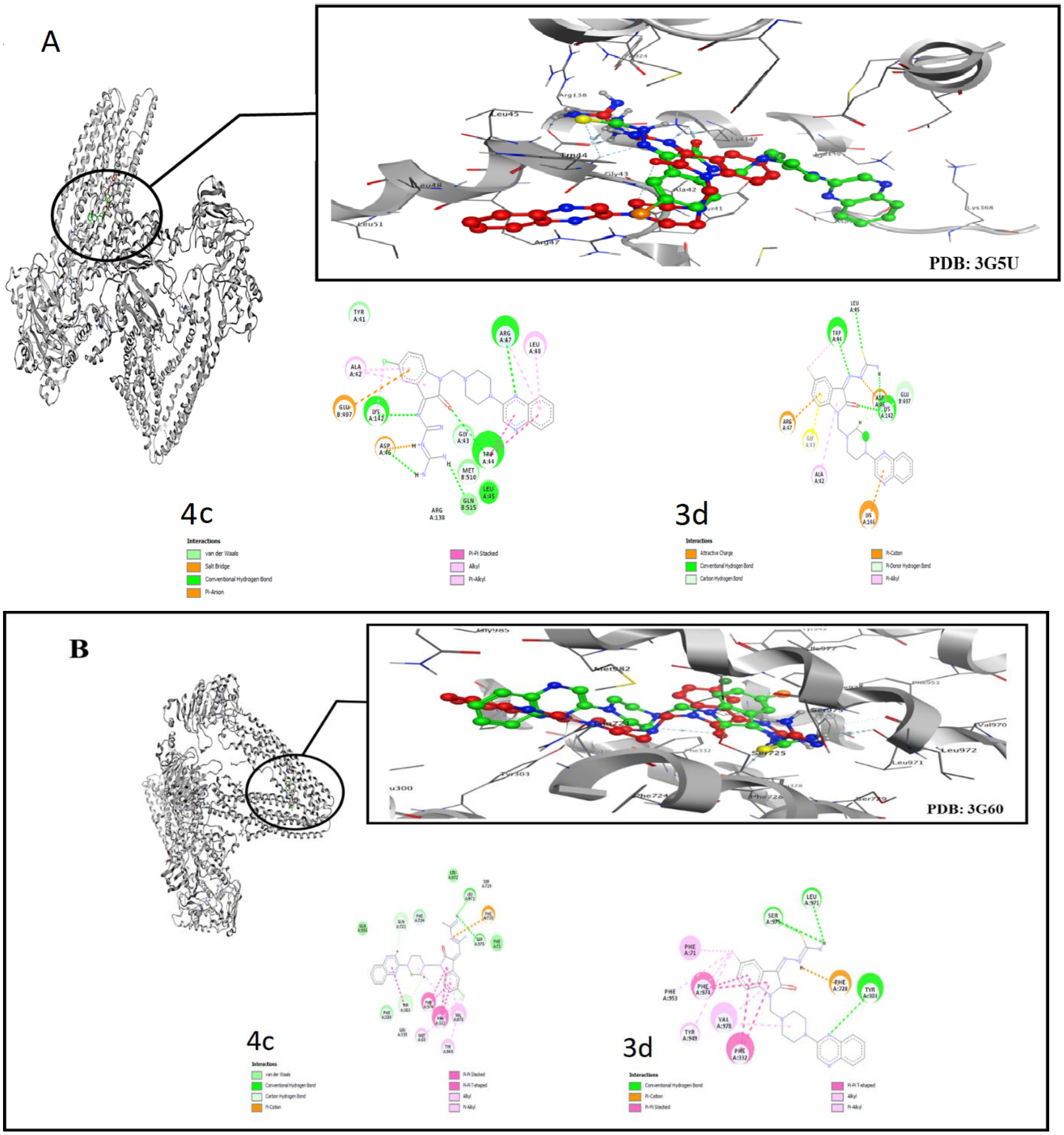
A
PDB: 3G5U
4c
3d
B
PDB: 3G60
4c
3d

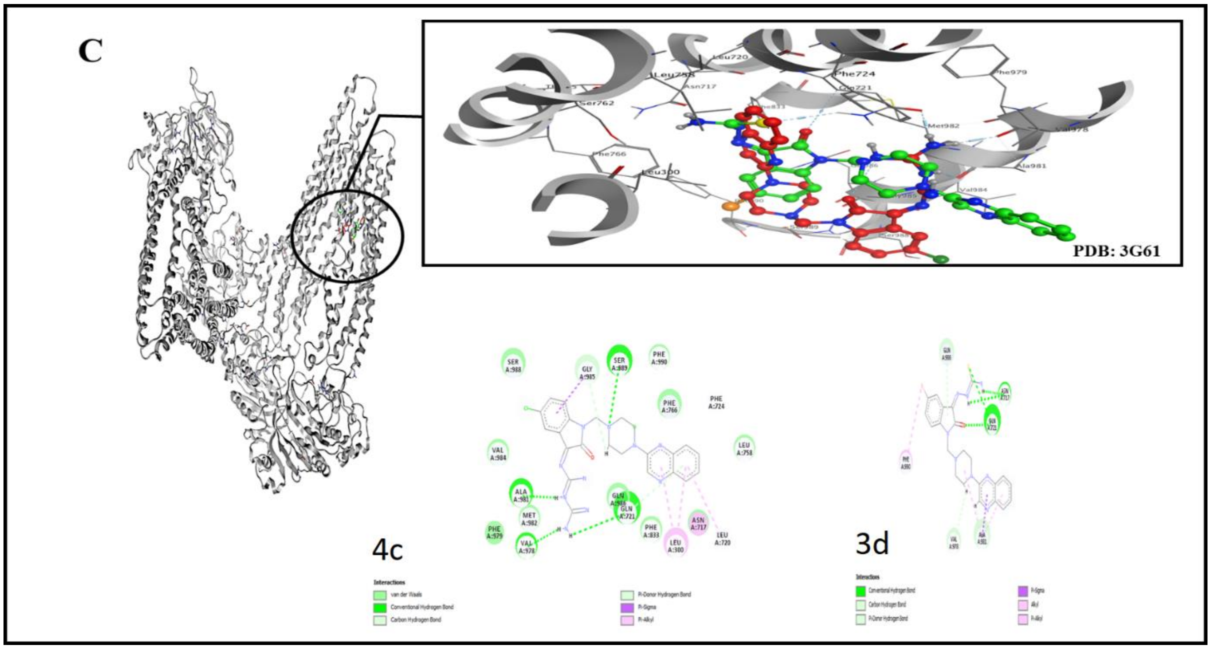
C
PDB: 3G61
4c
3d

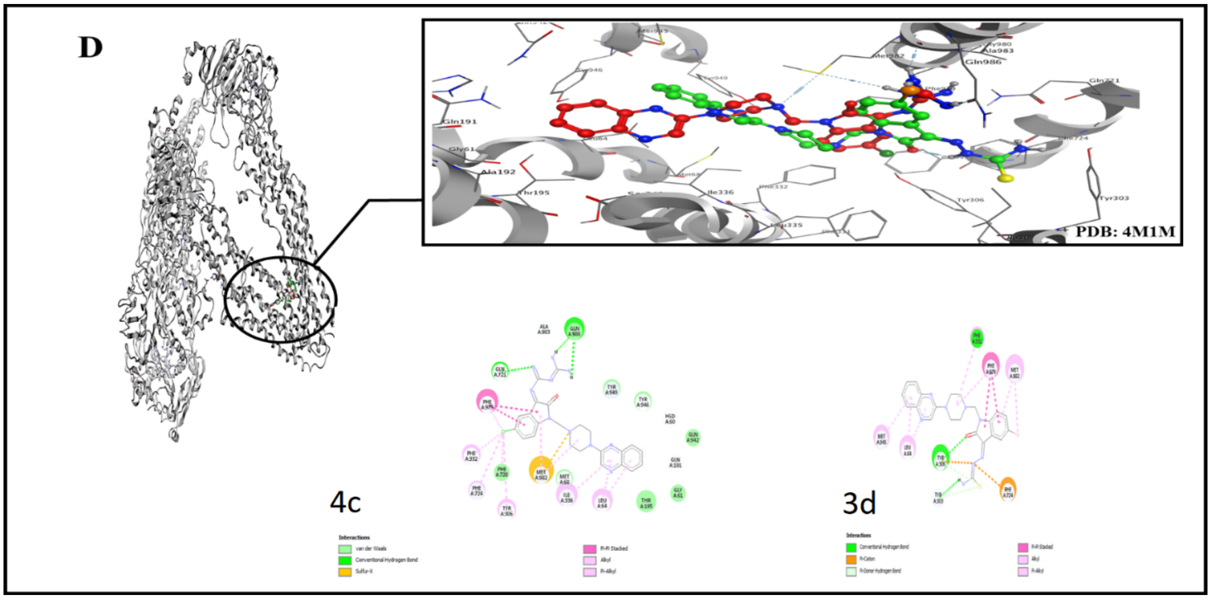
D
PDB: 4M1M
4c
3d

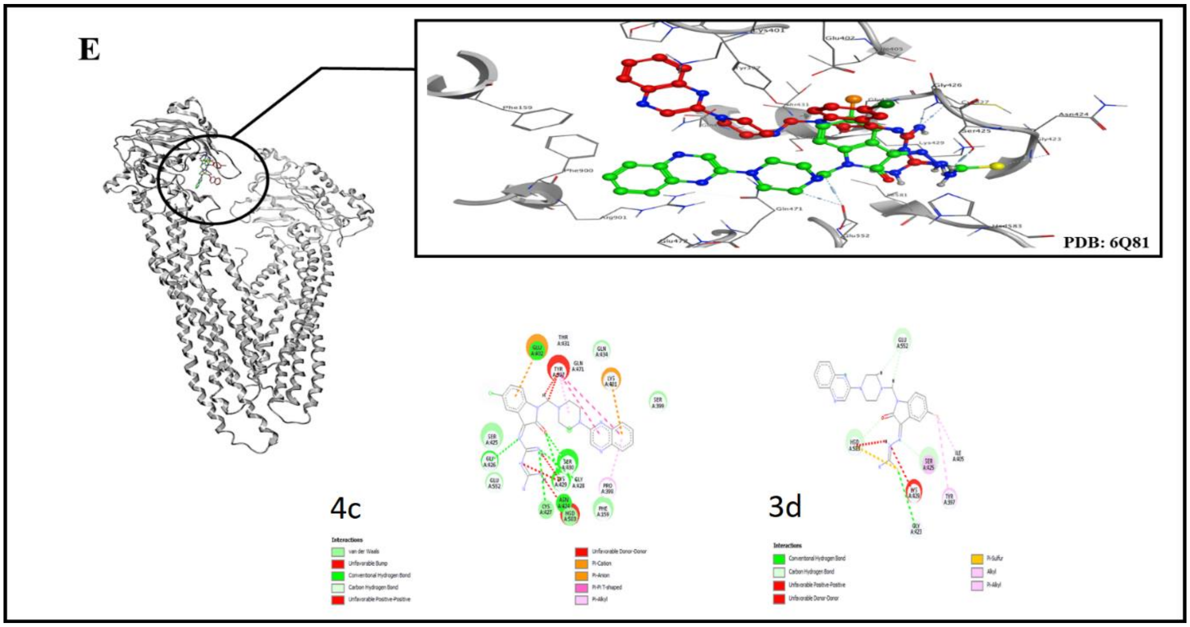

Figure S5: Important interactions of compounds 3d and 4c ligand with the receptors 3G5U (part A), 3G60 (part B), 3G61 (part C), 4M1M (part D) and 6Q81 (part E).

2. Materials and methods

2.1. Synthetic procedures for preparation of the main intermediate compound 2-piperazinyl quinoxaline (I)

The main initial substrate 2-(piperazin-1-yl) quinoxaline (I) was prepared from Butyl 2-oxoacetate (Scheme S1). The detailed explanation of these procedures can be found in our previously published research [1].

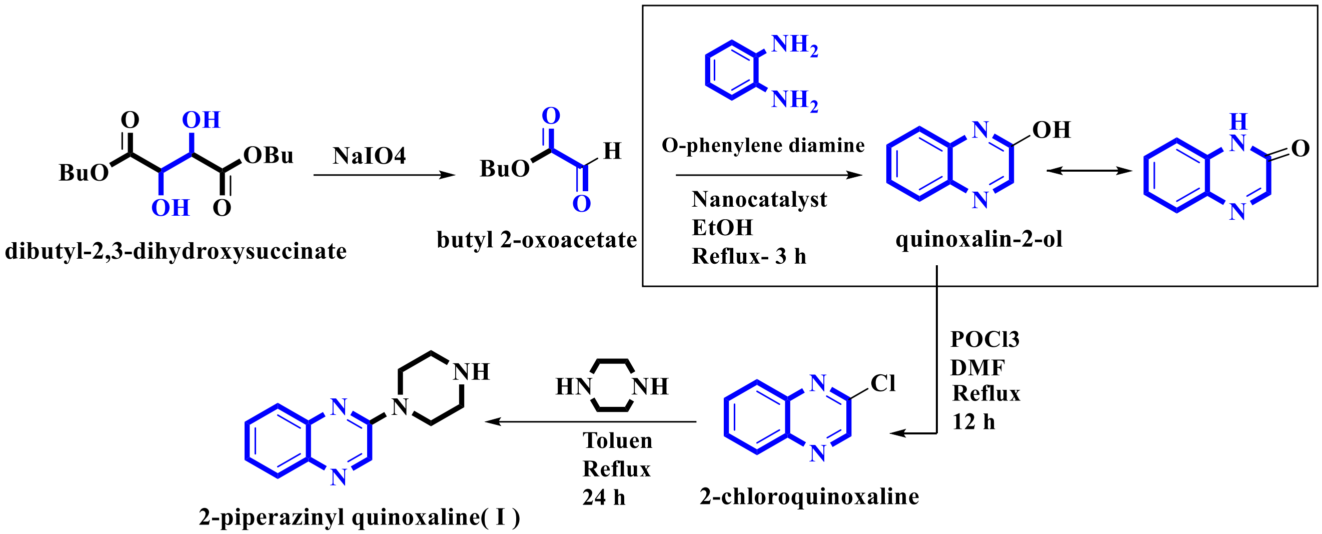

Scheme S1. Typical experimental procedure for the synthesis of quinoxaline-based intermediate compound (I).

2.2. General synthetic procedure for compounds 2a-2d

The final products (series A: 2a-2d) were synthesized through two different methods (via a one-pot multicomponent reaction in the presence of our newly synthesized nanocatalyst or via formation of iminium unstable intermediate in the absence of the nanocatalyst) as outlined in Scheme S2. The mentioned Mannich reaction can be performed via one pot three-component protocol in the presence of the acidic catalyst requiring (substituted) isatin, formaldehyde and a secondary amine containing main core (2-piperazinylquinoxaline), or via a preformed iminium ion requiring (substituted) isatin, formaldehyde and 2-piperazinylquinoxaline in the absence of the catalyst. Apart from catalytic procedure which can be found in our previously published research [1], 1mmol of 2-piperazinylquinoxaline with excess amounts of formaldehyde were dissolved in absolute ethanol and iminium ion formed after about 45min in reflux condition. Then this unstable intermediate in situ reacted with (substituted) isatin (Scheme S2) in reflux condition (Table S2) to furnish the desired N-Mannich 2-piperazinylquinoxaline derivatives (2a-2d).

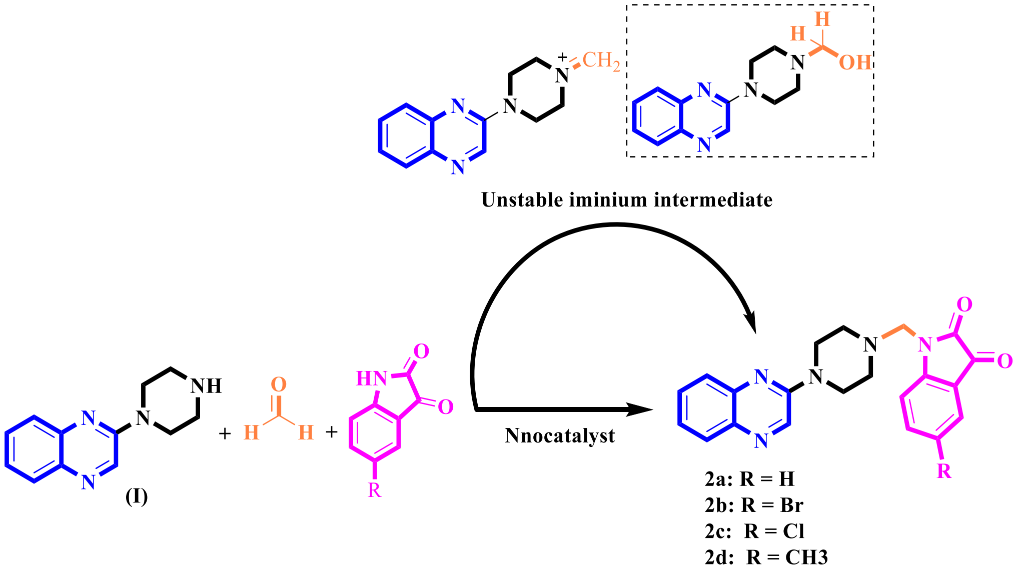

Scheme S2. General synthetic procedure for compounds 2a-2d.

Table S2. Two different procedures to produce final products 2a-2d.

Catalytic condition	solvent	Temperature	Time	Yields %
-	Absolute ethanol	Reflux	48-72h	45
Nanocatalyst	Absolute ethanol	Reflux	3h	95

2.3. General synthetic procedure for compounds 3a-3h and 4a-4d

The final products 3a-3h and 4a-4d were synthesized via two different methods (via a one-pot multicomponent reaction in the presence of the nanocatalyst or via formation of iminium unstable intermediate in the absence of the nanocatalyst) as outlined in Scheme S3 and Table S3. The Mannich reaction corresponding for the synthesis of compounds 3a-3h and 4a-4d can be performed via one pot four-component protocol in the presence of the acidic catalyst requiring (substituted) isatin, formaldehyde, 2-piperazinylquinoxaline as amine containing main core and an amine containing hetero acyclic tail (thio/semicarbazone or Metformine). In the absence of the catalyst,

synthesis of final products 3a-3h and 4a-4d is possible via formation of iminium ion as described above if only, at the second phase, this unstable intermediate in situ reacted with pre-synthesized compounds CO_1-CO_{12} (a hybrid molecule from (substituted) isatin and amine containing tail portion (thio/semicarbazone or Metformine).

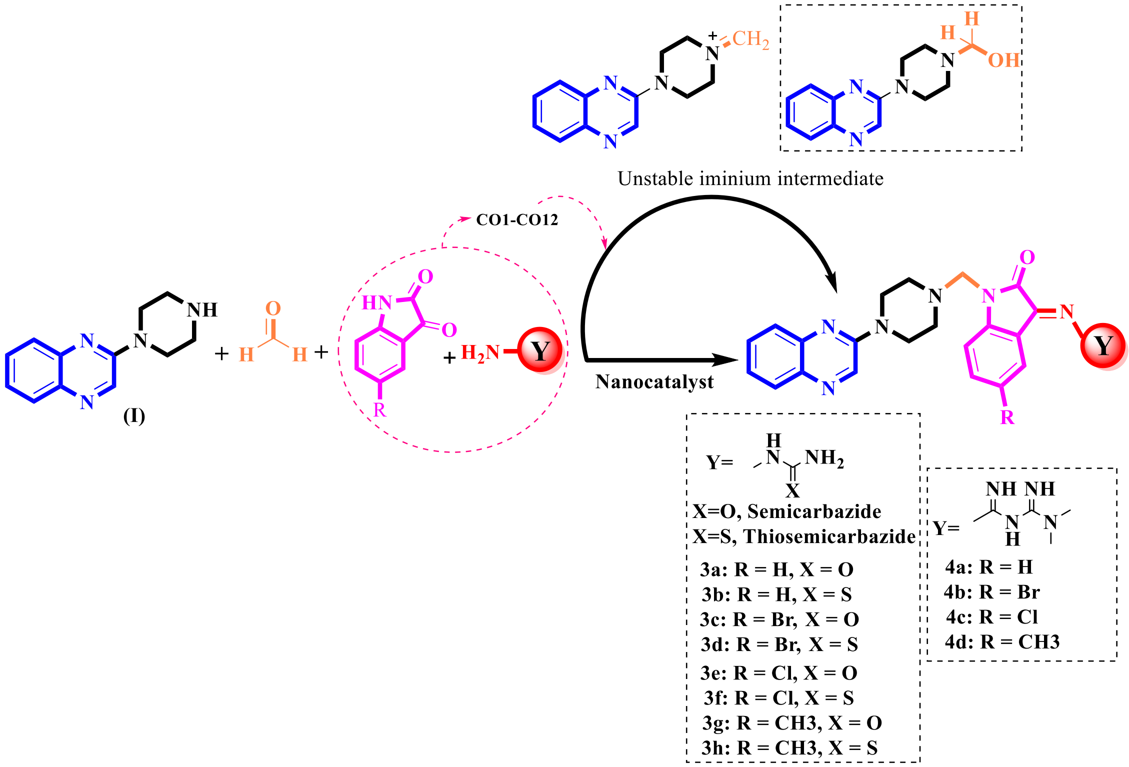

Scheme S3. General synthetic procedure for compounds 3a-3h and 4a-4d.

Table S3. Two different procedures to produce final products 3a-3h and 4a-4d.

Catalytic condition	solvent	Temperature	Time	Yields %
-	Absolute ethanol	Reflux	All at once, 48-72 h<br>Step by step, 48-72 h	-<br>40
Nanocatalyst	Absolute ethanol	Reflux	3h	95

2.4. General synthetic procedure for intermediate compounds CO_1-CO_{12}

The (substituted) isatin-3-thio/semicarbazones (CO_1-CO_8) and N, N dimethyl biguanidin imino isatin derivatives (CO_9-CO_{12}) can be produced in the presence or in the absence of the catalyst. These stable intermediate compounds were synthesized by condensation of isatin with thio/semicarbazone and metformine (N, N-dimethylbiguanide) as summarized in Scheme S4 and Table S4.

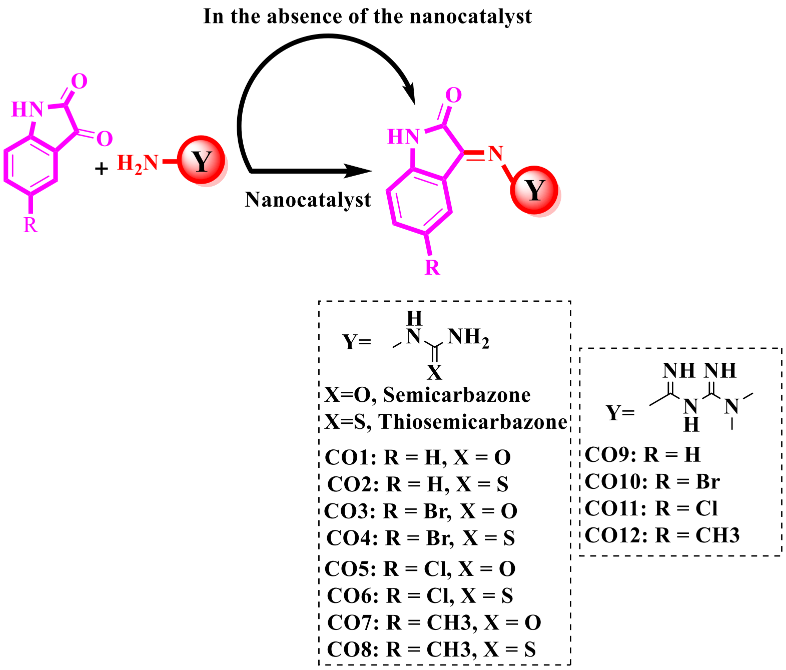

Scheme S4. General synthetic procedure for compounds CO1-CO12.

Table S4. Two different procedures to produce intermediate products CO1-CO12.

Catalytic condition	solvent	Temperature	Time	Yields %
GAA	Absolute ethanol	Reflux	48-72 h	60% yields
Nanocatalyst	Absolute ethanol	Reflux	150min	95% yields

2.5. Biological evaluations: In vitro cytotoxicity

This rapid assay is based on the reduction of tetrazolium salt to dark blue formazan product by mitochondrial enzymes of living, not dead cells after 24 and 48h exposure of cells to tested compounds at different concentrations. DMSO (with the less concentration than 1%) was the solvent in this biological test. serial concentrations were made by diluting stock solution of each compound (50µM) with culture media (RPMI 1640).

2.6. Molecular modeling

2.6.1. Docking studies

Protein-ligand docking was initiated using LeDock software (http://lephar.com). The structure of all compounds was sketched using HyperChem [12]. The geometry and energy of the structures were being optimized using ORCA software [8] at DFT, B3LYP/cc-pvdz level of theory. The chain A of the structure of c-Kit PDB ID 1T46 was selected for docking [9], cognate ligand and all crystallographic water molecules removed from the macromolecular structure using the LePro module (http://lephar.com). The docking parameters are set in a way that the center box of docking centered on the CB atom of Val603 as this is one of the key residues in the active site of the c-kit enzyme. The grid box was set to16 ×16 ×16 with a spacing value of 1.0 Å and the number of binding poses was set to 100. The best conformation with the least binding energy and better interacting residues was selected.

2.6.2. Molecular Dynamics Simulations

In order to explore which part of the ligands is responsible for their layout in the active site of the enzyme and consequently their inhibitory activities, the three compounds were chosen from the best docking pose from each different subset. Each of the three chosen ligands as well as the

crystallographic one as a control bound to the protein has been simulated for a period of 100 ns molecular dynamic simulation in explicit water. For each ligand, full Amber topology/coordinate files were created using the AmberTools package [10]. Using the antechamber program of AmberTools The VDW and bonded parameters for the ligands were taken from the general amber force field (GAFF) [11]. Partial atomic charges were then assigned based on the RESP charge Derive Server [12]. The AMBER format files of ligands were converted to the GROMACS format using the ACPYPE python tool [13].

Each of the complexes was solvated in the TIP3P water model and ions were introduced into the system to make the whole system neutral. All MD simulations were carried out by the GROMACS 2020.1 package [14]. Amber99sb-ildn force field from the investigation of Beauchamp et al. [20] was chosen to describe the protein behaviour. Periodic boundary conditions were applied in all three directions of space. Each system became energy minimized with a steeped descent algorithm. After minimization, the NVT followed by the NPT ensemble was applied while the Ca atoms of protein were positioned restrained. The NVT ensemble was adopted at a constant temperature of 300 K and with a coupling constant of 0.1 ps in modified Berendsen thermostat [15] with time duration of 500 ps. After stabilization of temperature, 1 ns NPT simulation was performed in which Berendsen [16] barostat was employed with a coupling constant of 2.0 ps at 1 bar. The production run in NPT and the time step of 2 fs was the followed step where the restraint was removed. The particle mesh Ewald (PME) method interaction was used [17] for long-range electrostatic. A 12 Å cutoff for long-range and the LINKS algorithm for H-bond constraints were applied [18] in both the equilibration and production run

2.7. Molecular docking

Molecular docking is a structure-based drug design (SBDD) method which is used significantly in calculations related to the interaction between ligand and protein; Because these interactions play an important and effective role in biochemical and biological processes; Also, in the field of molecular docking studies. As a result, by using this molecular modeling technique, the final result reported as a best pose of binding energy, can lead to a stable ligand-protein complex [19]. In the computational studies on the structure of Pgps, we can point to the docking and molecular dynamic simulation of inhibitors related to this structure by Isca and his colleagues, which was performed on PDB ID: 3G60 and 3G61 and investigated the placement of ligands in the active site of the protein [20, 21]. Palestro et all evaluated different methods as the best tools for virtual screening on the structures they proposed as P-gp's inhibitors, and finally introduced Vina and Autodock as suitable tools. In this study, the docking was on PDB IDs: 3G60 and 3G61 too [22], In this study, we used computational approaches containing molecular docking to investigate the binding affinity of two selected ligands with selected murine P-gp crystallographic structures.

In this study, we chose two selected and suitable ligands and molecular docking employed with murine P-gp crystallographic structures. Selected PDB IDs were: 3G5U, 3G60, 3G61, 4M1M and 6Q81 which are accessible in PDB databank (https://www.rcsb.org/). First, the desired ligands were drawn by Pymol [23] and optimized with MOPAC sofware [24] and did quantum semi-empirical optimizations by using AM1 method. Then, we used Pymol to convert files to Mol2 format file. After that, we employed Ledock software [25], that genetic algorithm (GA) is used in this package to optimize the position of the docked ligand (such as orientation, and rotatable bonds) and finally do molecular docking of the selected two ligands and each of the proteins. The dimensions of the box were 40 * 40 * 40 A˚ and we performed 90 docking runs for each compound.

The analytical and spectroscopic data for the unknown final products (2a-2d, 3a-3h, 4a-4d)

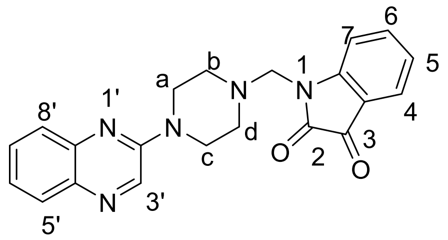

1-((4-(quinoxalin-2-yl) piperazin-1-yl) methyl) indoline-2,3-dione (2a)

Reddish rang crystals; IR (KBr, v_{max}, cm^{-1}): 1735 (N-C=O), 1687 (C=O), 2777 (Ar-CH), 1491 (C=N), 1027 (C–N) cm^{-1}; ^{1}H NMR (600 MHz, $CDCl_3$, DMSO) δ: 2.75-2.78 (m, 4H, b-2H, d-2H), 3.78-3.81 (m, 4H, a-2H, c-2H), 4.53 (s, 2H, CH_2), 7.13-7.18 (m, 1H, 6'-H), 7.36-7.44 (m, 2H, 5-H, 6-H), 7.55- 7.58 (d, J = 8.8 Hz, 1H, 5'-H), 7.59-7.62 (m, 2H, 4-H, 7-H) 7.66-7.72 (m, 1H, 7'-H), 7.80-7.83 (d, J = 8.8 Hz, 1H, 8'-H), 8.84 (s, 1H, 3'-H); ^{13}C NMR (150 MHz, $CDCl_3$, DMSO) δ: 44.57 (2C), 50.46 (2C), 62.38, 111.60, 117.64, 124.06, 125.02, 125.45, 126.56, 128.69, 130.18, 135.64, 136.93, 138.42, 141.52, 151.45, 152.08, 158.95(C=O),182.98(C=O); MS: (m/z) M^+ Anal. calcd for $C_{21}H_{19}N_5O_2$: 373.1, Found: 373.1

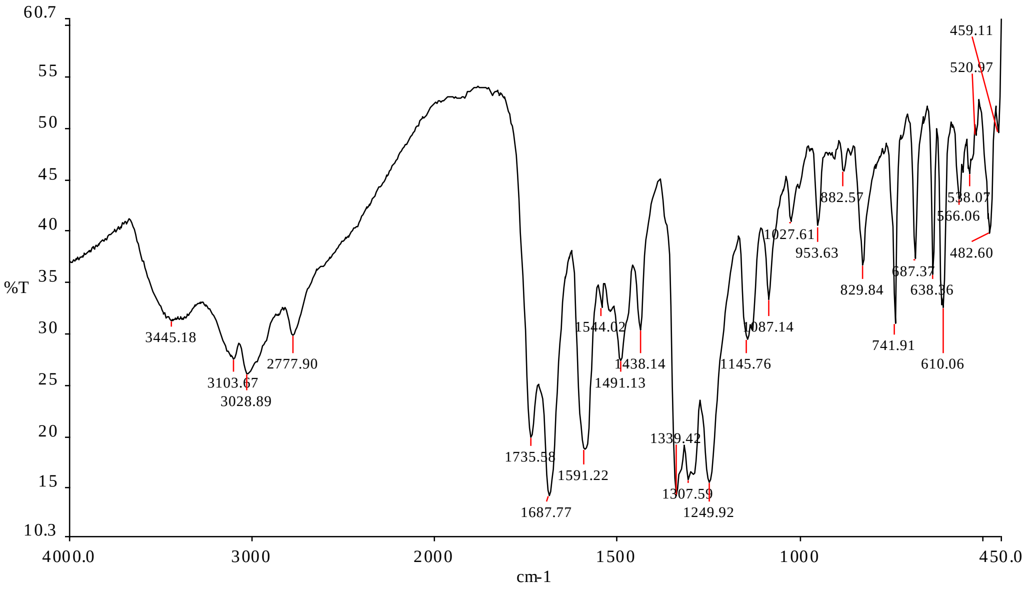

Fig S1. (FT-IR). 1-((4-(quinoxalin-2-yl) piperazin-1-yl) methyl) indoline-2,3-dione (2a)

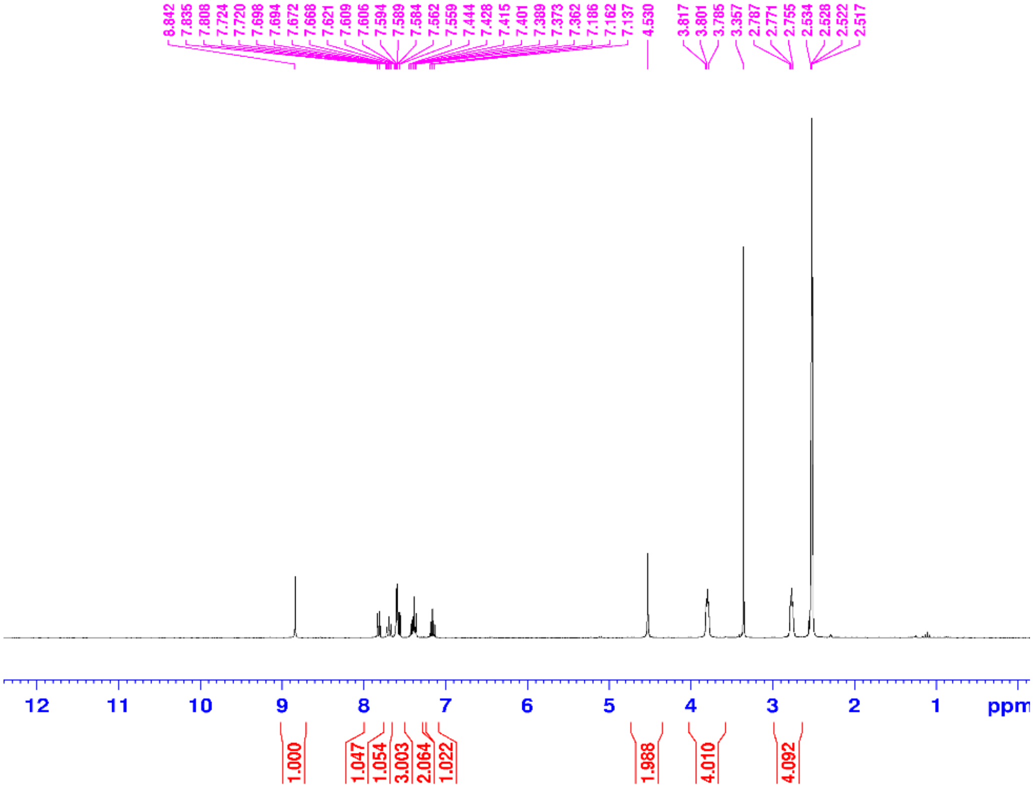

Fig S2. ^{1}HNMR Spectra.1-((4-(quinoxalin-2-yl) piperazin-1-yl) methyl) indoline-2,3-dione (2a)

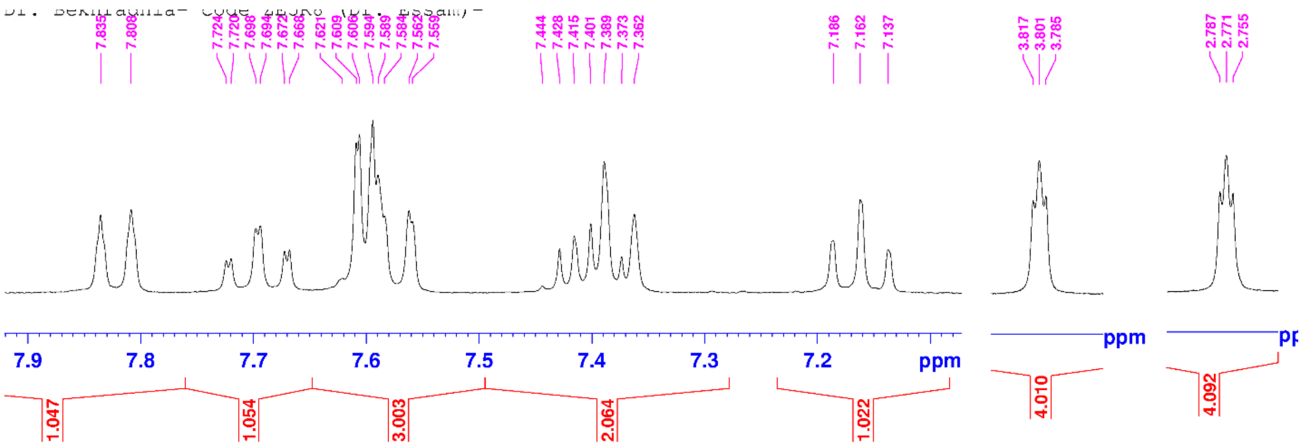

Fig S3. ^{1}HNMR expand spectra- aliphatic and aromatic regions. 1-((4-(quinoxalin-2-yl) piperazin-1-yl) methyl) indoline-2,3-dione (2a)

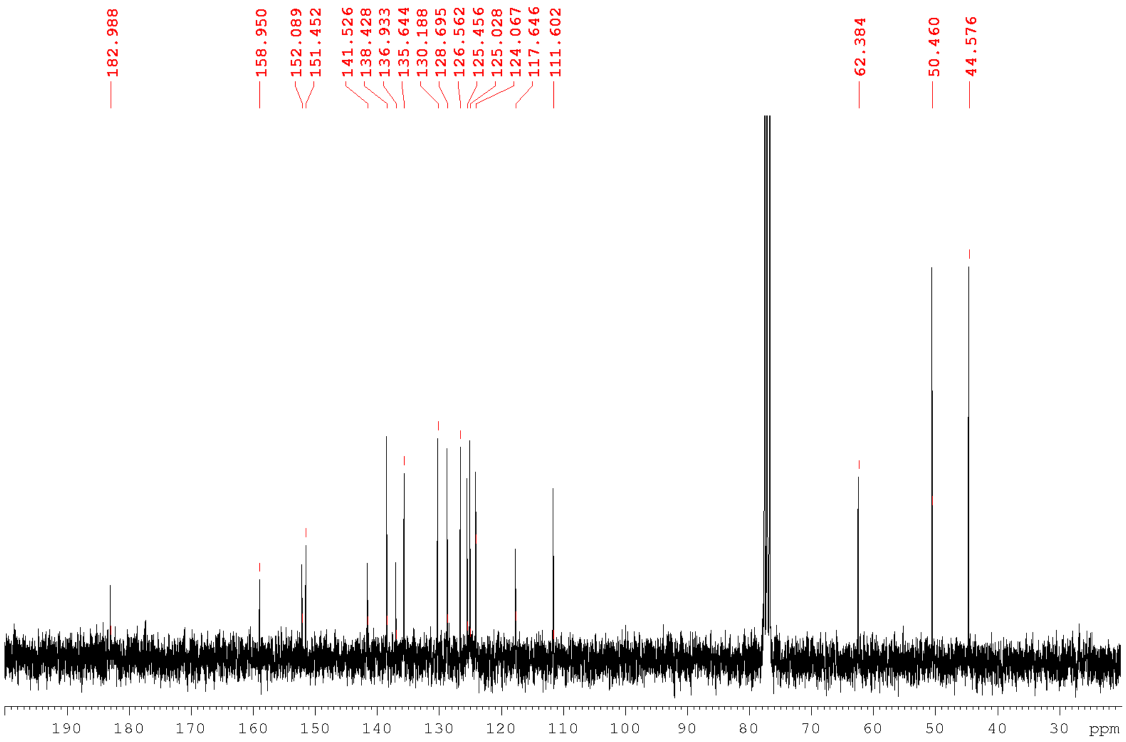

Fig S4. ^{13}CNMR Spectra. 1-((4-(quinoxalin-2-yl) piperazin-1-yl) methyl) indoline-2,3-dione (2a)

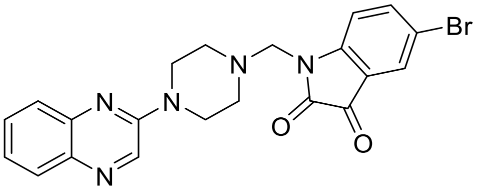

5-bromo-1-((4-(quinoxalin-2-yl) piperazin-1-yl) methyl) indoline-2,3-dione (2b)

Reddish orange crystals; IR (KBr, v_{max}, cm^{-1}): 1727 (N-C=O), 1579 (C=O), 2841 (Ar-CH), 1482 (C=N), 1067 (C–N), 757 (C-Br), cm^{-1}; ^{1}H NMR (600 MHz, $CDCl_3$, DMSO) δ: 2.72-2.76 (m, 4H, b-2H, d-2H), 3.83-3.86 (m, 4H, a-2H, c-2H), 4.19 (s, 2H, CH_2), 6.92-6.95 (d, j = 8, 1H, 7-H), 7.01-7.04 (d, j = 8, 1H, 6-H) 7.27 (s, 1H, 4-H), 7.40-7.45 (m, 1H, 7'-H), 7.58-7.63 (m, 1H, 6'-H), 7.70-7.73 (d, j = 8.8 , 1H, 5’-H), 7.89-7.91 (d, j = 8.8 , 1H, 8’-H), 8.59 (s, 1H, 3'-H); ^{13}C NMR (150 MHz, $CDCl_3$, DMSO) δ: 44.76 (2C), 51.23 (2C), 73.54, 111.79, 115.85, 124.76, 126.50, 126.93,

128.62, 130.12, 131.74, 132.72, 135.73, 136.75, 141.72, 142.68, 152.38, 166.39(C=O), 176.96(C=O); MS: (m/z) M^+ Anal. calcd for $C_{21}H_{14b}rN_5O_2$: 451.0, Found: 451.0

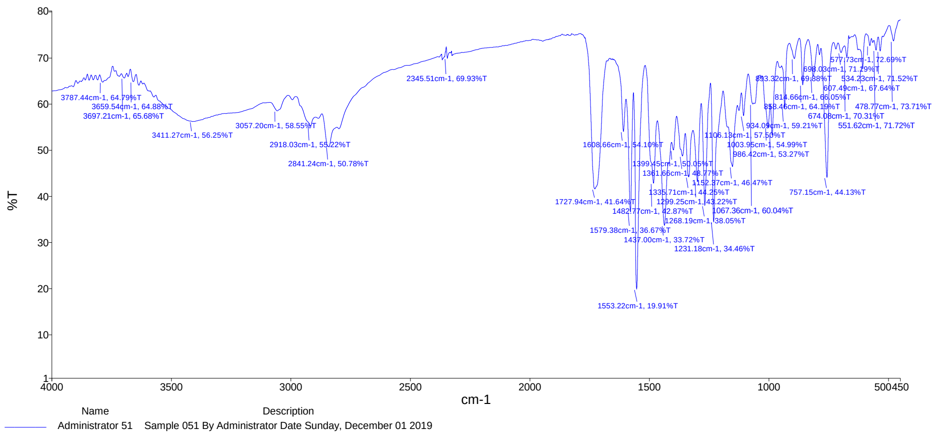

Fig S5. (FT-IR). 5-bromo-1-((4-(quinoxalin-2-yl) piperazin-1-yl) methyl) indoline-2,3-dione (2b)

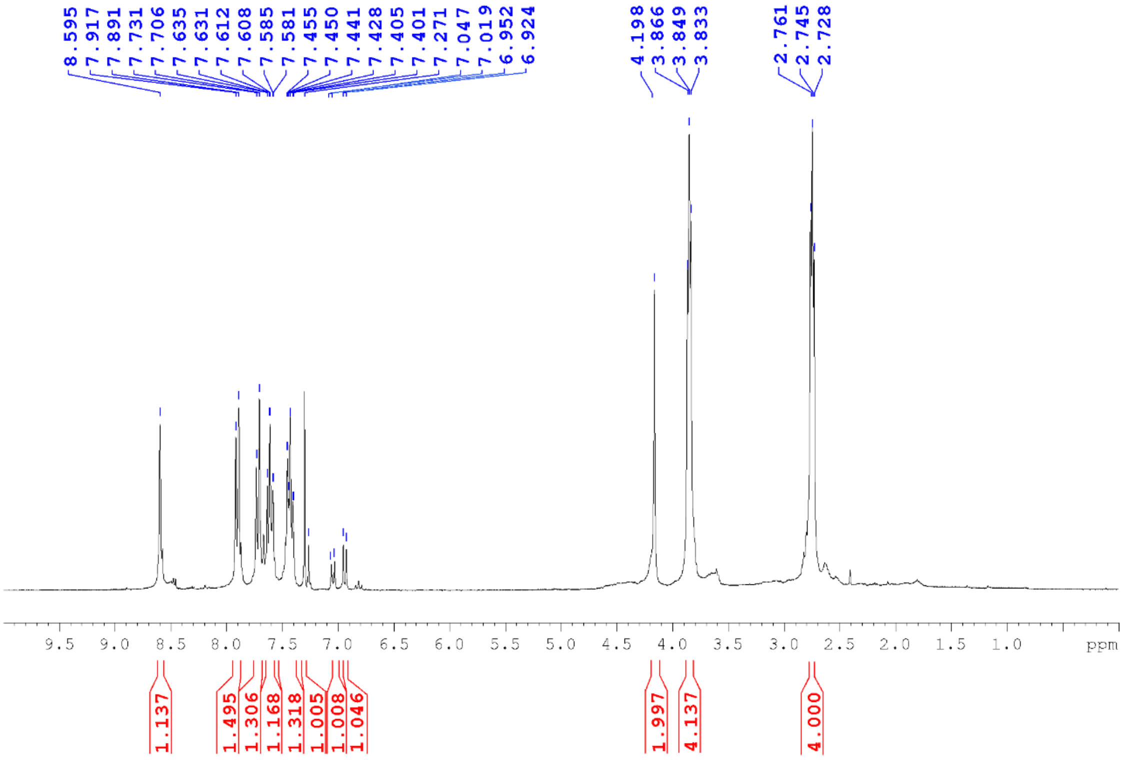

Fig S6. ^{1}HNMR Spectra. 5-bromo-1-((4-(quinoxalin-2-yl) piperazin-1-yl) methyl) indoline-2,3-dione (2b)

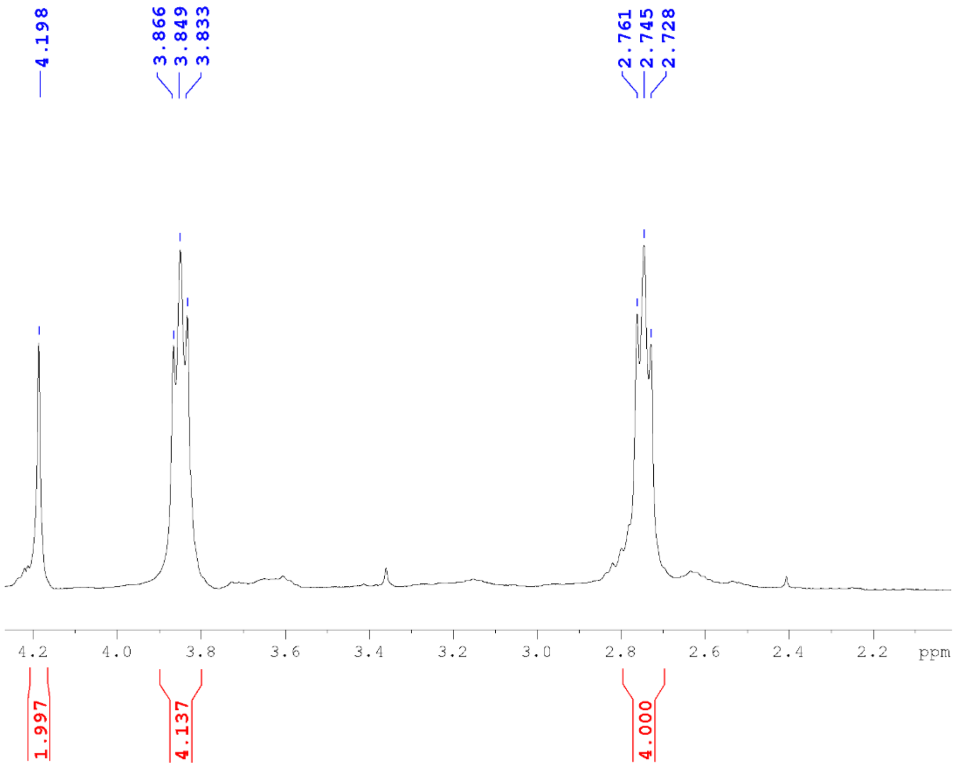

Fig S7. ^{1}HNMR Spectra. Expand- aliphatic region. 5-bromo-1-((4-(quinoxalin-2-yl) piperazin-1-yl) methyl) indoline-2,3-dione (2b)

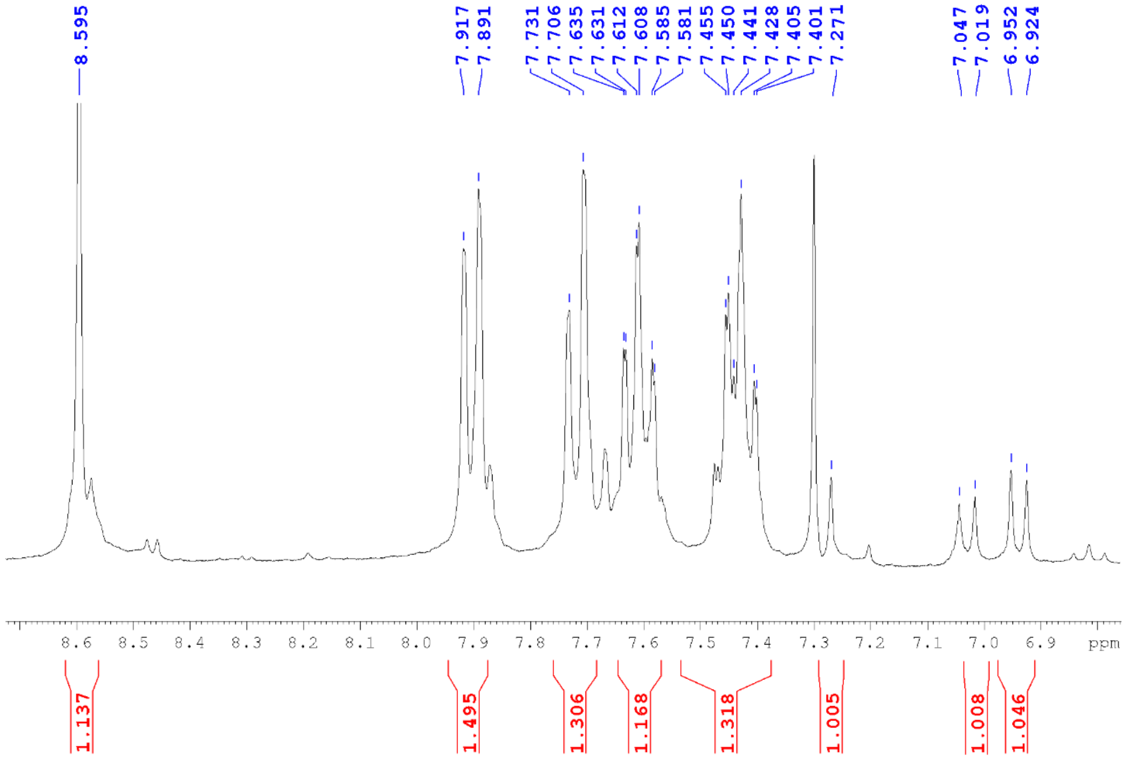

Fig S8. ^{1}HNMR Spectra. Expand- aromatic region. 5-bromo-1-((4-(quinoxalin-2-yl) piperazin-1-yl) methyl) indoline-2,3-dione (2b)

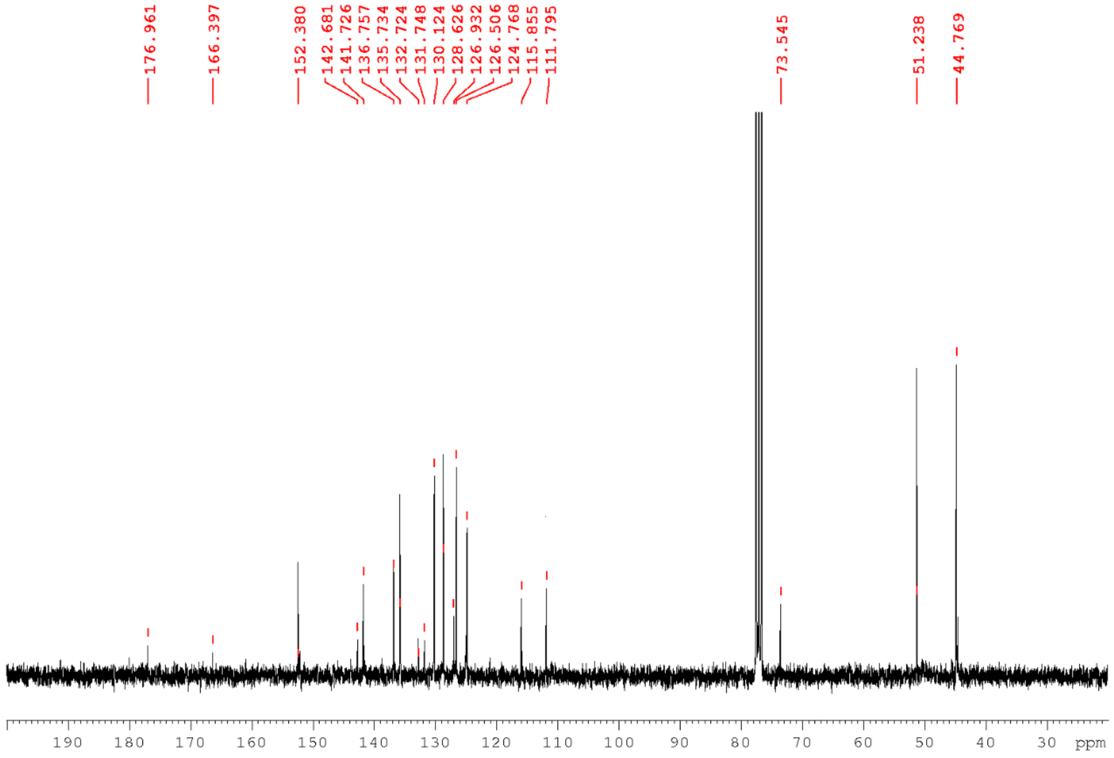

Fig S9. ^{13}**CNMR Spectra.** 5-bromo-1-((4-(quinoxalin-2-yl) piperazin-1-yl) methyl) indoline-2,3-dione (2b)

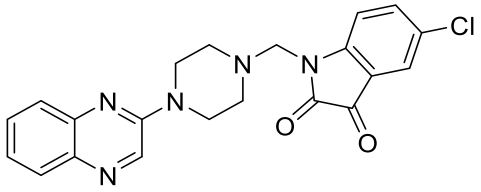

5-chloro-1-((4-(quinoxalin-2-yl) piperazin-1-yl) methyl) indoline-2,3-dione (2c)

Orange crystals; IR (KBr, ν_{max}, cm^{-1}): 1722 (N-C=O), 1636 (C=O), 2951 (Ar-CH), 1446 (C=N), 1068 (C–N), 693 (C-Cl), cm^{-1}; ^{1}H NMR (600 MHz, $CDCl_3$, DMSO) δ: 2.75-2.78 (m, 4H, b-2H, d-2H), 3.73-3.76 (m, 4H, a-2H, c-2H), 4.37 (s, 2H, CH_2), 6.97-6.99 (d, j =8, 1H, 7-H), 7.27-7.31 (m, 1H, 6'-H), 7.35 (s, 1H, 4-H), 7.40-7.42 (d, j = 8 , 1H, 6-H), 7.55-7.59 (m, 1H, 7'-H), 7.63-7.66 (d, j = 8.8 , 1H, 5'-H), 7.83-7.85 (d, j = 8.8 , 1H, 8'-H), 8.44 (s, 1H, 3'-H); ^{13}C NMR (150 MHz, $CDCl_3$, DMSO) δ: 44.50 (2C), 50.23 (2C), 73.56, 111.30, 124.15, 124.91, 126.47, 128.46, 128.56,

129.79, 130.18, 131.36, 135.50, 136.57, 141.56, 142.18, 152.08, 162.5(C=O), 177.21(C=O); MS: (m/z) M^+ Anal. calcd for $C_{21}H_{14c}lN_5O_2$: 407.1, Found: 407.1

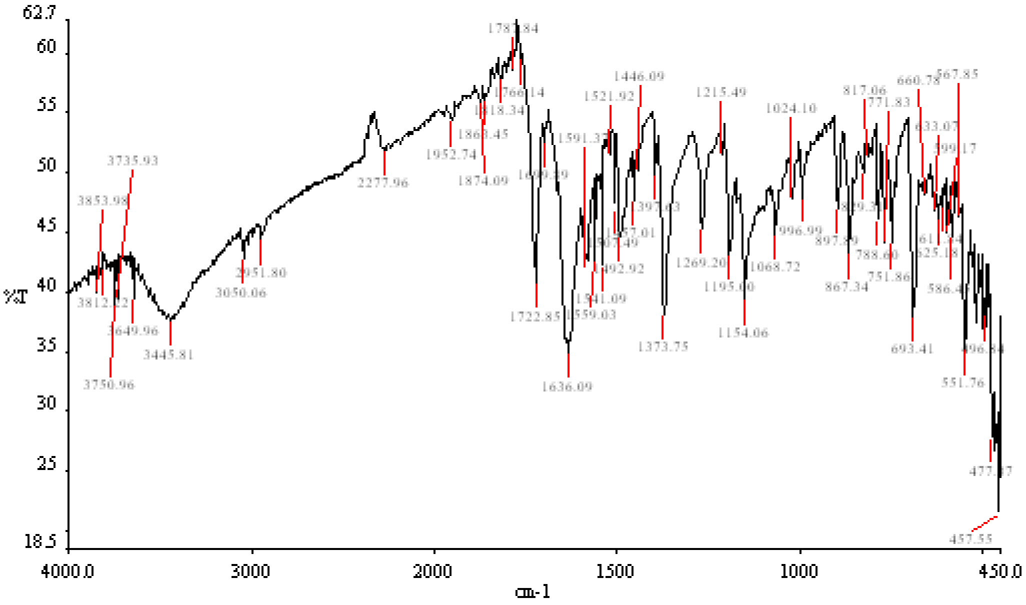

Fig S10. (FT-IR). 5-chloro-1-((4-(quinoxalin-2-yl) piperazin-1-yl) methyl) indoline-2,3-dione (2c)

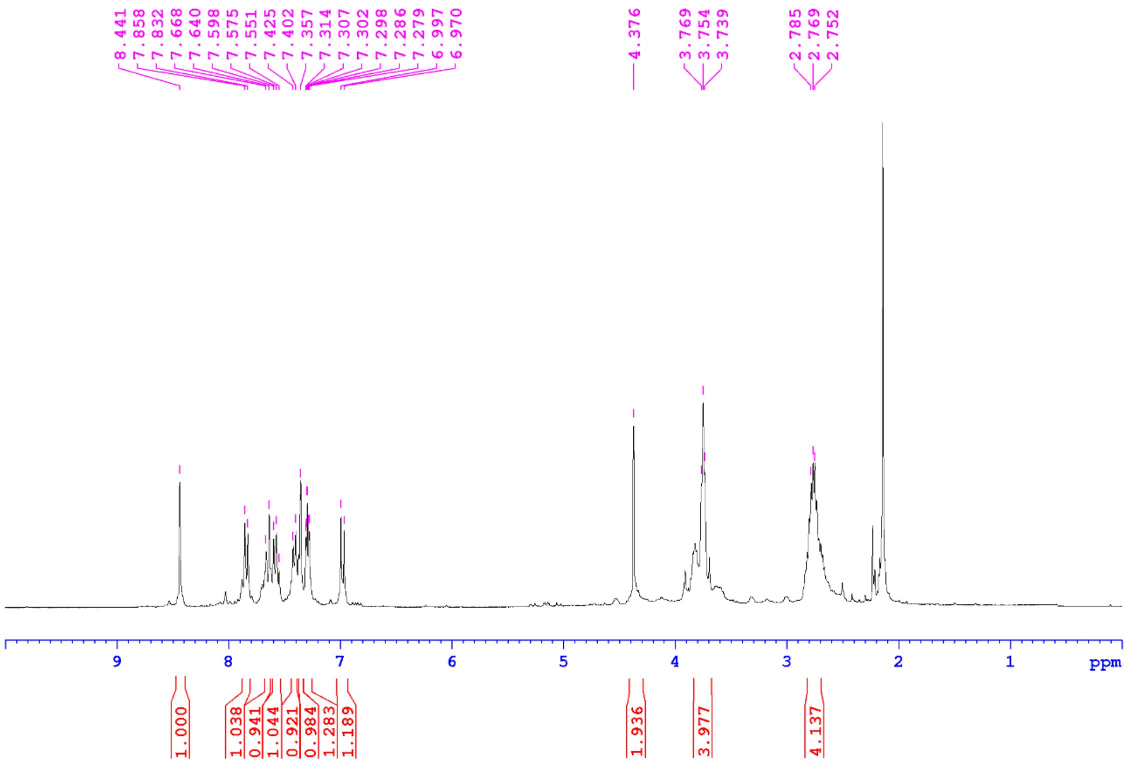

Fig S11. ^{1}HNMR Spectra. 5-chloro-1-((4-(quinoxalin-2-yl) piperazin-1-yl) methyl) indoline-2,3-dione (2c)

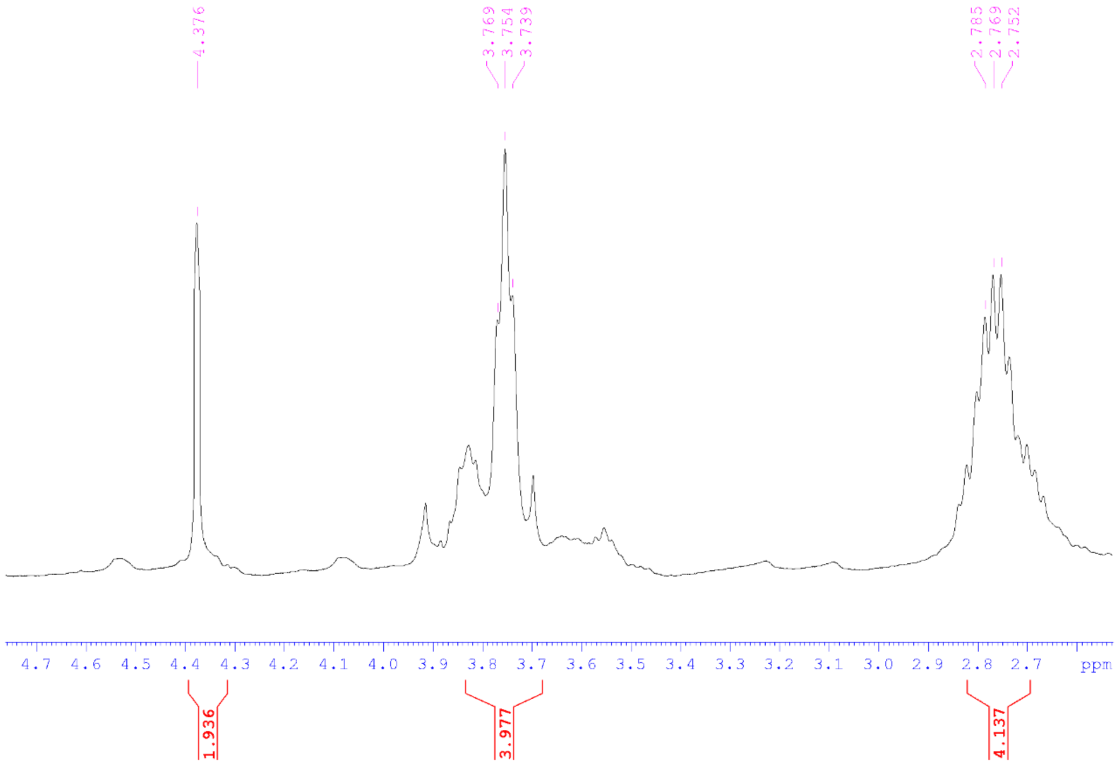

Fig S12. ^{1}HNMR Spectra. Expand- aliphatic region. 5-chloro-1-((4-(quinoxalin-2-yl) piperazin-1-yl) methyl) indoline-2,3-dione (2c)

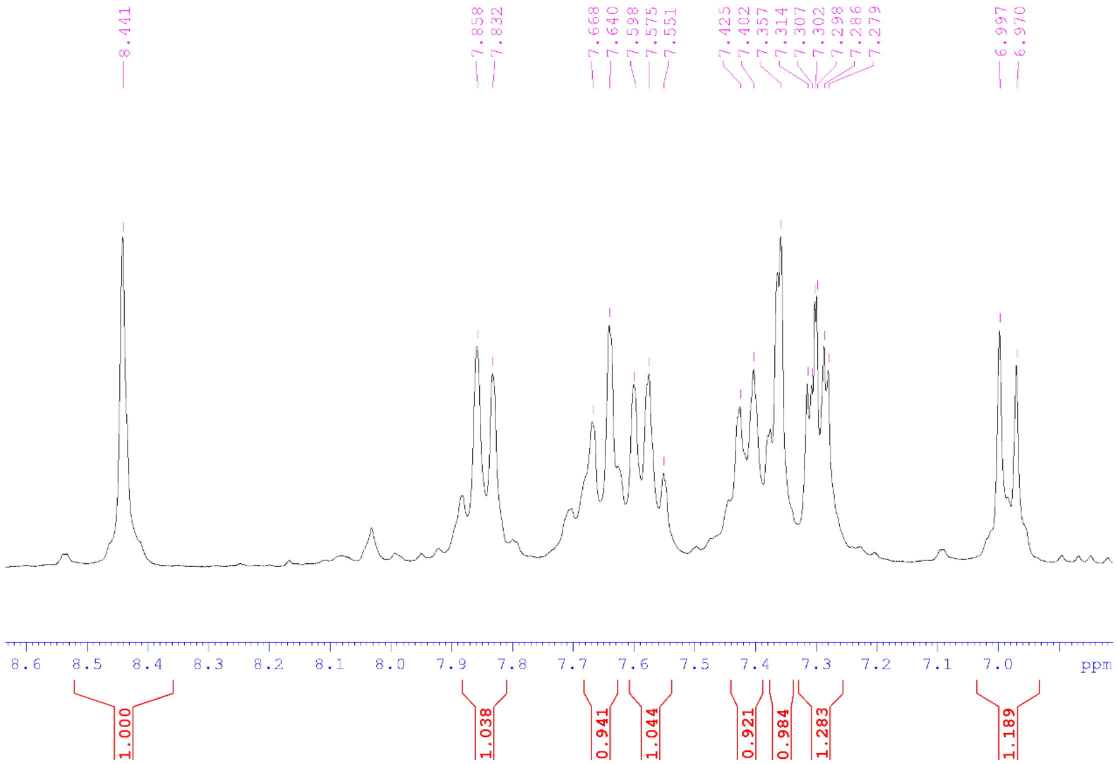

Fig S13. ^{1}HNMR Spectra. Expand- aromatic region. 5-chloro-1-((4-(quinoxalin-2-yl) piperazin-1-yl) methyl) indoline-2,3-dione (2c)

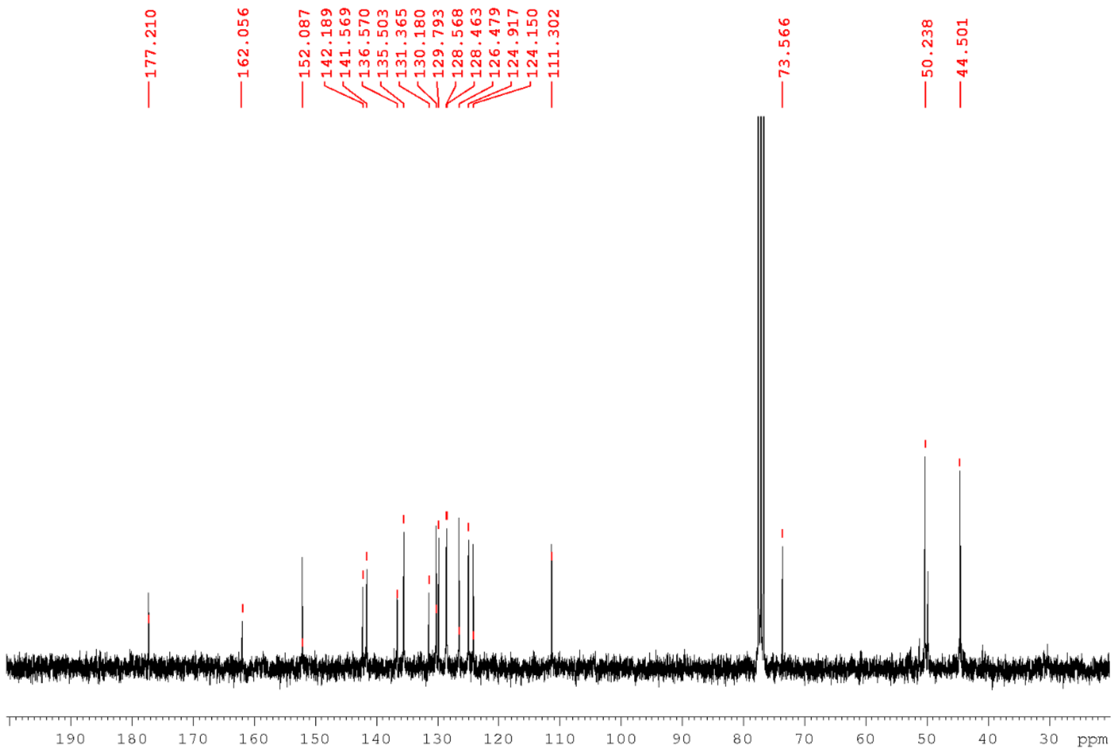

Fig S14. ^{13}CNMR Spectra. 5-chloro-1-((4-(quinoxalin-2-yl) piperazin-1-yl) methyl) indoline-2,3-dione (2c)

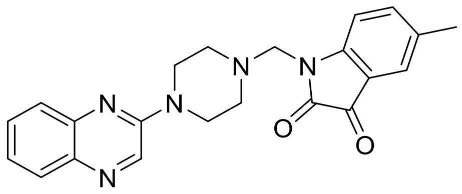

5-methyl-1-((4-(quinoxalin-2-yl) piperazin-1-yl) methyl) indoline-2,3-dione (2d)

Reddish Orange crystals; IR (KBr, v_{max}, cm^{-1}): 1738 (N-C=O), 1676 (C=O), 2917 (Ar-CH), 1445 (C=N), 1098 (C–N), cm^{-1}; ^{1}H NMR (600 MHz, $CDCl_3$, DMSO) δ: 2.36 (s, 3H, CH_3), 2.80-2.82 (m, 4H, b-2H, d-2H), 3.81-3.83 (m, 4H, a-2H, c-2H), 4.55 (s, 2H, CH_2), 7.02-7.03 (d, j= 8, 1H, 7-H), 7.40-7.44 (m, 2H, 6-H, 6’-H), 7.45 (s, 1H, 4-H), 7.57-7.60 (m, 1H, 7’-H), 7.67-7.69 (d, j= 8.8, 1H, 5’-H), 7.87-7.89 (d, j= 8.8, 1H, 8’-H), 8.56 (s, 1H, 3’-H); ^{13}C NMR (150 MHz, $CDCl_3$, DMSO) δ: 20.69, 44.58 (2C), 50.44 (2C), 62.34, 111.39, 117.65, 124.99, 125.70, 126.55, 128.69, 130.17,

133.92, 135.64, 136.92, 138.88, 141.53, 149.31, 152.09, 159.11(C=O), 183.23(C=O); MS: (m/z) M^+ Anal. calcd for $C_{22}H_{21}N_5O_2$: 387.1, Found: 387.1

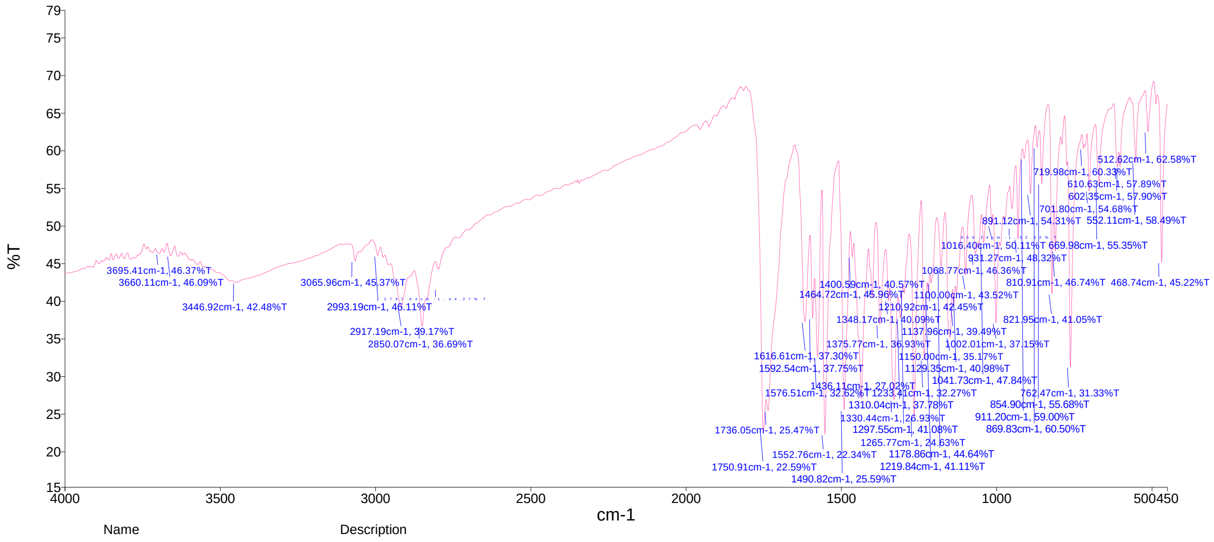

Fig S15. (FT-IR). 5-methyl-1-((4-(quinoxalin-2-yl) piperazin-1-yl) methyl) indoline-2,3-dione (2d)

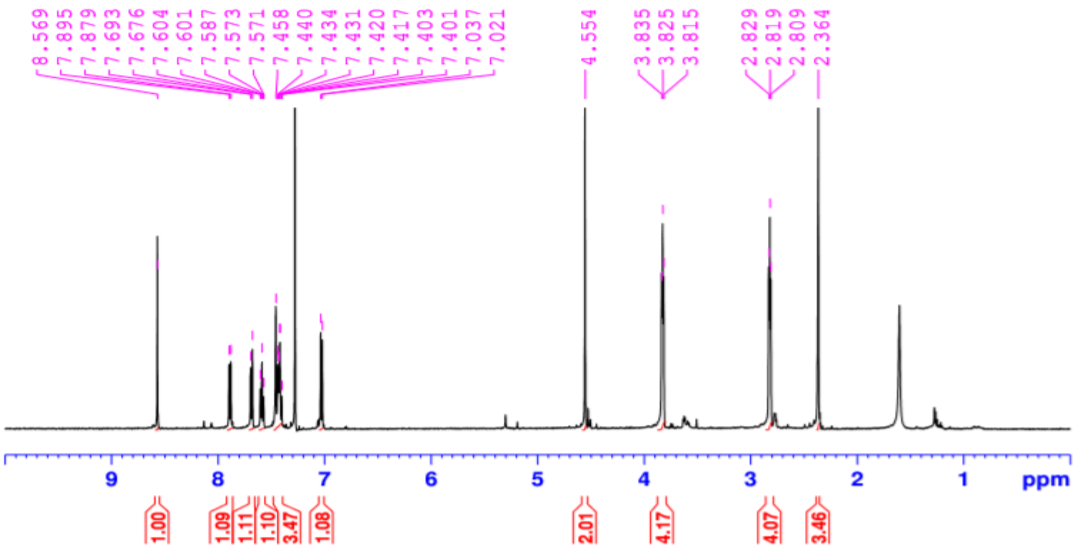

Fig S16. ^{1}HNMR Spectra. 5-methyl-1-((4-(quinoxalin-2-yl) piperazin-1-yl) methyl) indoline-2,3-dione (2d)

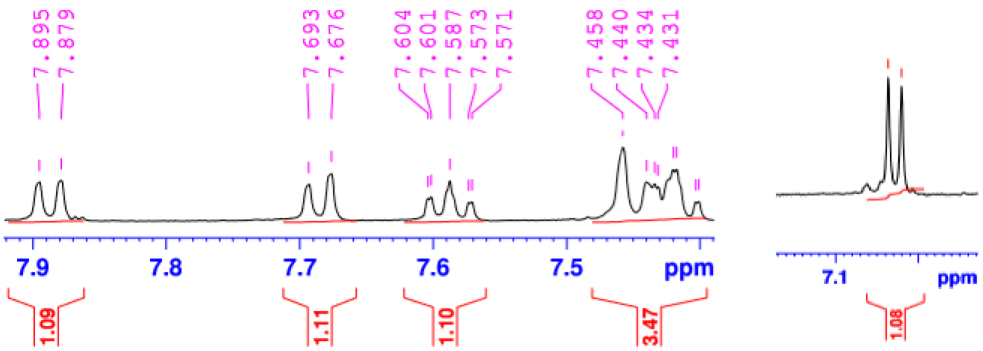

Fig S17. ¹HNMR Spectra. Expand- aromatic region. 5-methyl-1-((4-(quinoxalin-2-yl) piperazin-1-yl) methyl) indoline-2,3-dione (2d)

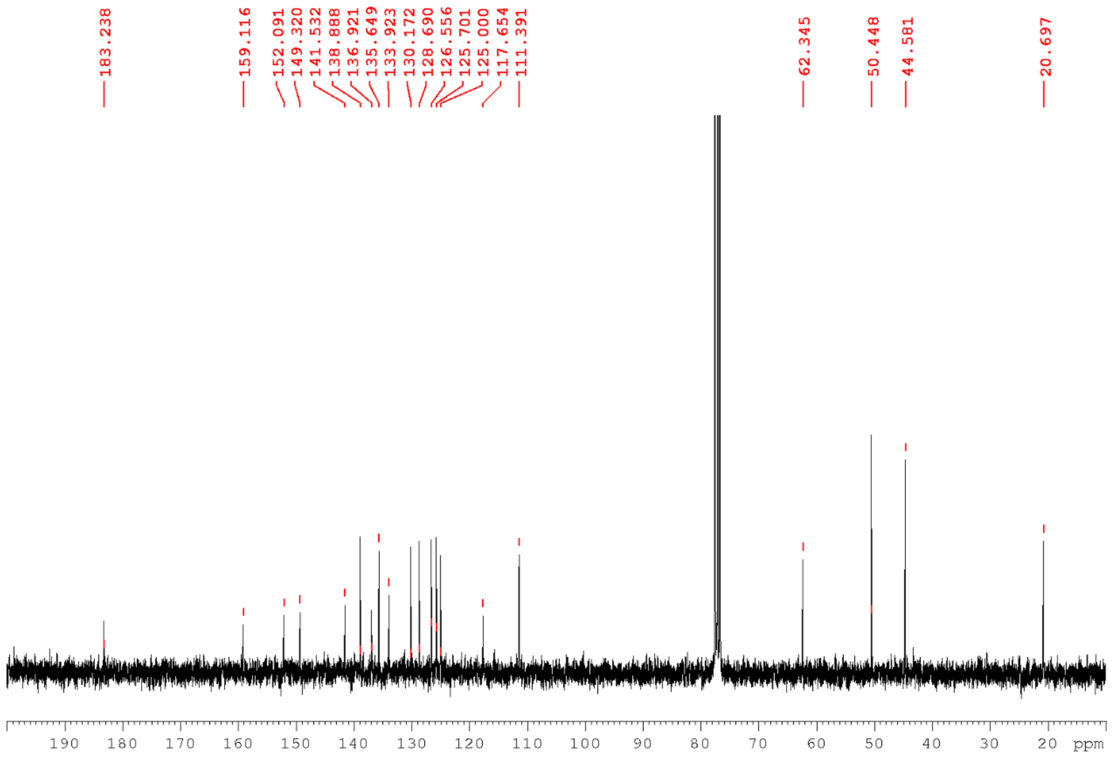

Fig S18. ¹³CNMR Spectra. 5-methyl-1-((4-(quinoxalin-2-yl) piperazin-1-yl) methyl) indoline-2,3-dione (2d)

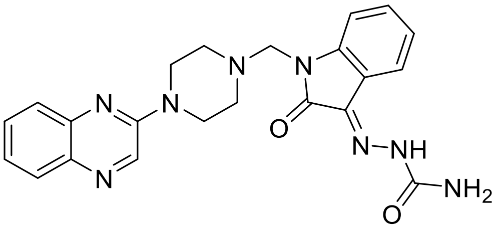

2-(2-oxo-1-((4-(quinoxalin-2-yl) piperazin-1-yl) methyl) indolin-3-ylidene) hydrazine-1-carboxamide (3a)

Orange crystals; IR (KBr, v_{max}, cm^{-1}): 3411 (N-H), 1678 (N-C=O), 1685 (N-C=O), 2851 (Ar-CH), 1436 (C=N), 1046 (C–N), cm^{-1}; ^{1}H NMR (600 MHz, $CDCl_3$, DMSO) δ: 2.81-2.83 (m, 4H, b-2H, d-2H), 3.64-3.65 (d, j= 6, 1H, NH_2-H), 3.81-3.83 (m, 4H, a-2H, c-2H), 4.62 (s, 2H, CH_2), 4.91-4.92 (d, j= 6, 1H, NH_2-H), 7.09-7.11 (m, 1H, 6-H), 7.14-7.17 (m, 1H, 5-H), 7.38-7.41 (m, 1H, 6'-H), 7.56-7.61 (m, 2H, 4-H, 7-H), 7.66-7.68 (m, 1H, 7'-H), 7.87-7.88 (d, j= 8.8, 1H, 5'-H), 8.54-8.55 (d, j= 8.8, 1H, 8'-H), 8.56 (s, 1H, 3'-H), 11.96 (s, 1H, NH); ^{13}C NMR (150 MHz, $CDCl_3$, DMSO) δ: 44.56 (2C), 50.50 (2C), 70.66, 110.38, 119.67, 120.40, 123.38, 124.94, 126.52, 128.63, 130.15, 130.78, 130.97, 135.62, 136.82, 141.54, 143.06, 152.04, 154.43 (C=O), 162.17 (C=O); MS: (m/z) M^+ Anal. calcd for $C_{22}H_{22}N_8O_2$: 430.1, Found: 430.1

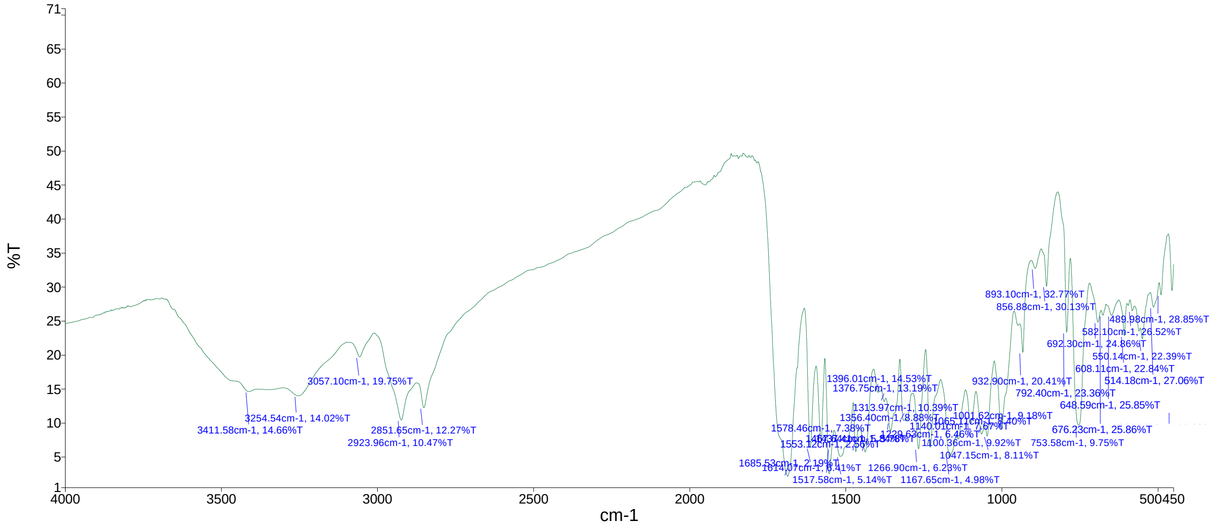

Fig S19. (FT-IR). 2-(2-oxo-1-((4-(quinoxalin-2-yl) piperazin-1-yl) methyl) indolin-3-ylidene) hydrazine-1-carboxamide (3a)

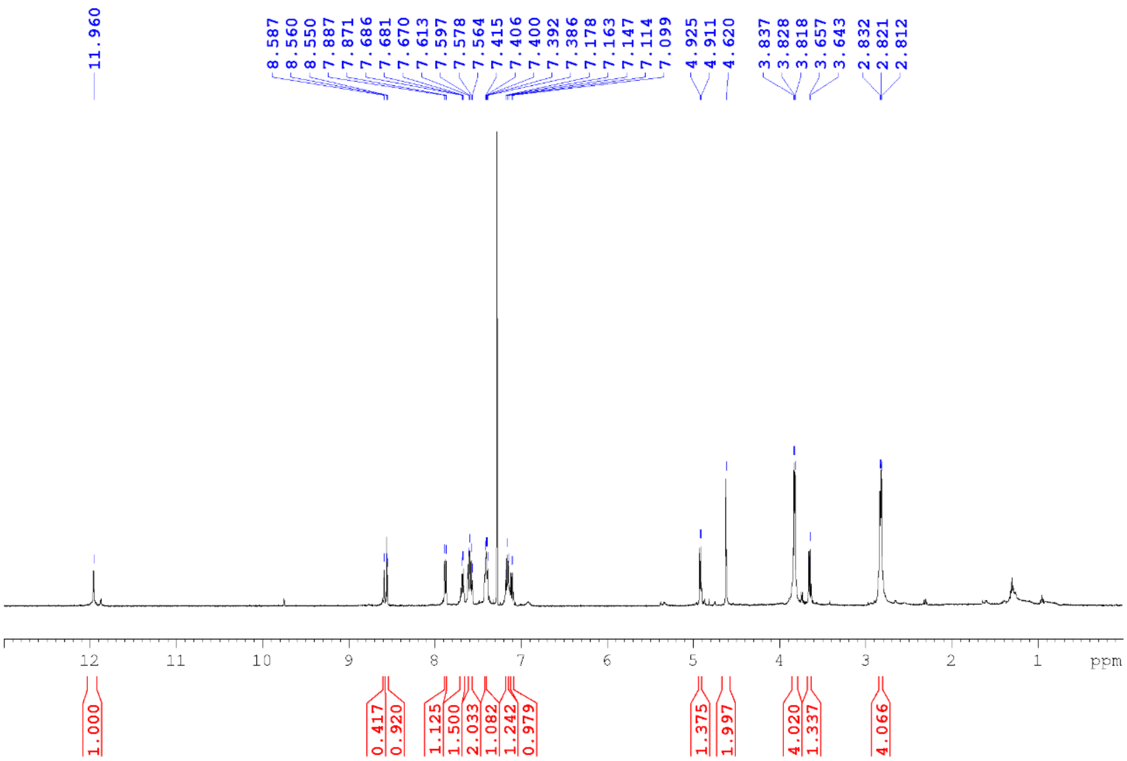

Fig S20. ^{1}HNMR Spectra. 2-(2-oxo-1-((4-(quinoxalin-2-yl) piperazin-1-yl) methyl) indolin-3-ylidene) hydrazine-1-carboxamide (3a)

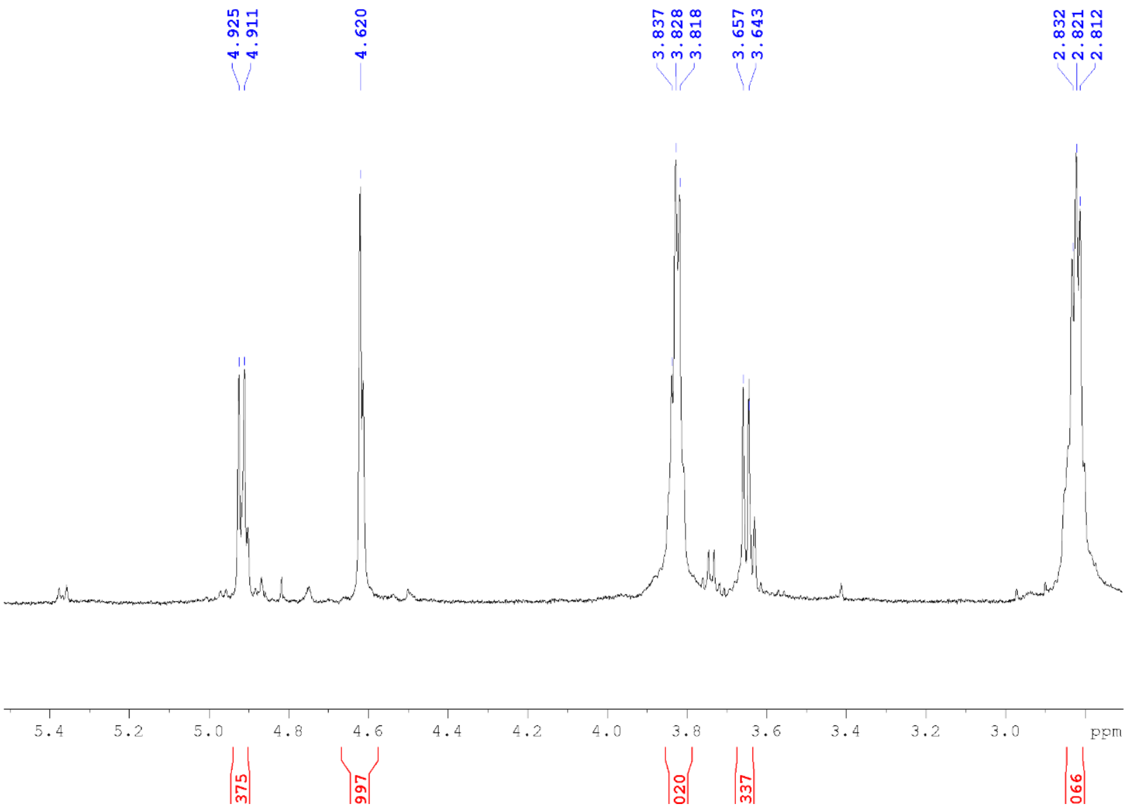

Fig S21. ^{1}HNMR Spectra. Expand- aliphatic region. 2-(2-oxo-1-((4-(quinoxalin-2-yl) piperazin-1-yl) methyl) indolin-3-ylidene) hydrazine-1-carboxamide (3a)

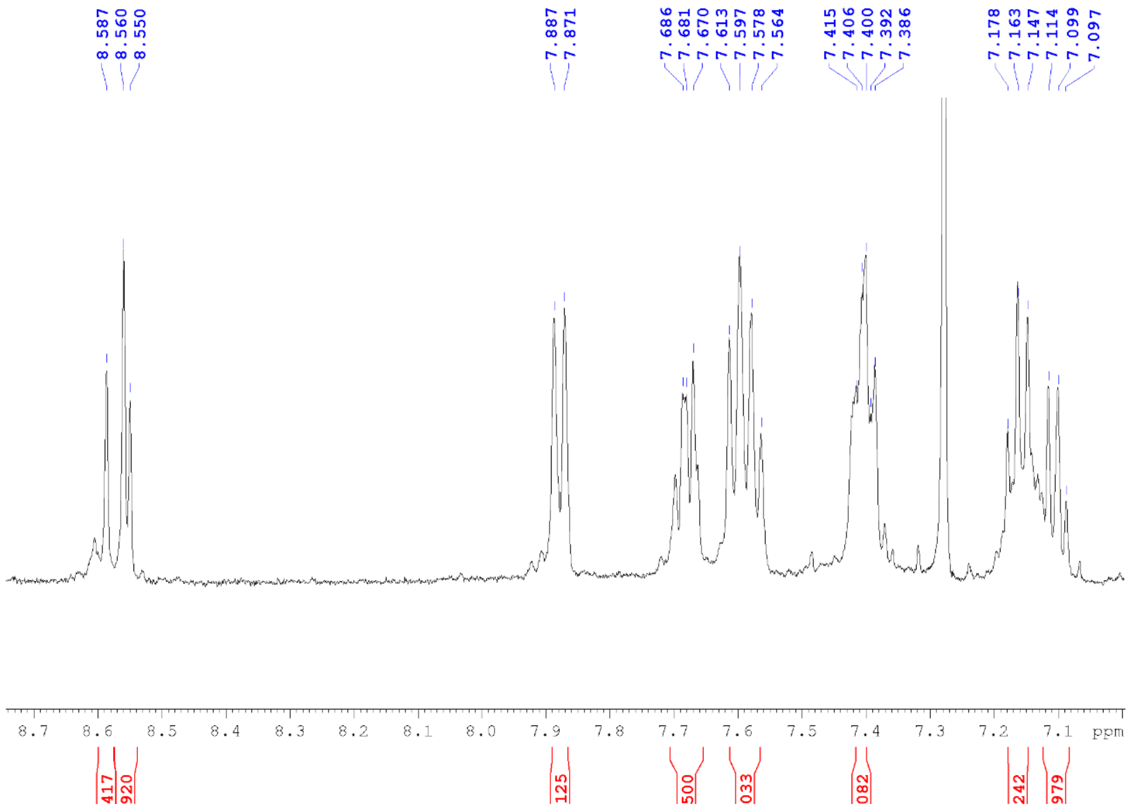

Fig S22. ¹HNMR Spectra. Expand- aromatic region. 2-(2-oxo-1-((4-(quinoxalin-2-yl) piperazin-1-yl) methyl) indolin-3-ylidene) hydrazine-1-carboxamide (3a)

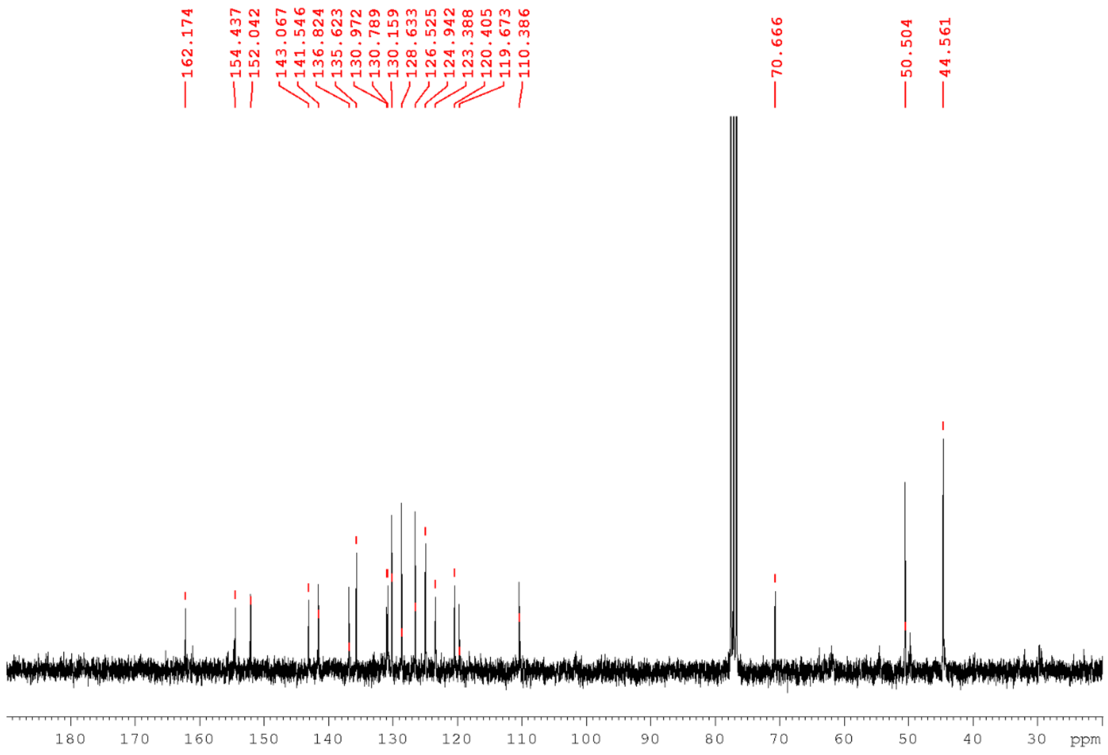

Fig S23. ^{13}CNMR Spectra. 2-(2-oxo-1-((4-(quinoxalin-2-yl) piperazin-1-yl) methyl) indolin-3-ylidene) hydrazine-1-carboxamide (3a)

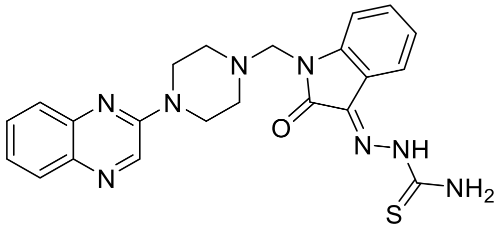

2-(2-oxo-1-((4-(quinoxalin-2-yl) piperazin-1-yl) methyl) indolin-3-ylidene) hydrazine-1-carbothioamide (3b)

Orange crystals; IR (KBr, v_{max}, cm^{-1}): 3465 (N-H), 1579 (N-C=O), 1691 (N-C=S), 2917 (Ar-CH), 1437 (C=N), 1067 (C–N), cm^{-1}; ^{1}H NMR (600 MHz, $CDCl_3$, DMSO) δ: 2.81-2.83 (m, 4H, b-2H, d-2H), 3.82-3.84 (m, 4H, a-2H, c-2H), 4.62 (s, 2H, CH_2), 4.78-4.79 (d, j= 6, 1H, NH_2-H), 5.31-5.32 (d, j= 6, 1H, NH_2-H), 7.09-7.11 (m, 1H, 5-H), 7.14-7.18 (m, 1H, 6-H), 7.39-7.44 (m, 2H, 4-H, 7-H), 7.56-7.60 (m, 1H, 6'-H), 7.67-7.69 (m, 1H, 7'-H), 7.87-7.88 (d, j= 8.8, 1H, 5'-H), 8.55-

8.56 (d, j= 8.8, 1H, 8'-H), 8.59 (s, 1H, 3'-H), 12.82 (s, 1H, NH); ^{13}C NMR (150 MHz, $CDCl_3$, DMSO) δ: 44.55 (2C), 50.53 (2C), 74.45, 110.56, 119.34, 120.85, 123.50, 124.93, 126.52, 128.61, 130.15, 131.09, 131.45, 135.69, 136.79, 141.54, 143.71, 152.13, 161.93(C=O), 179.24(C=S); MS: (m/z) M^+ Anal. calcd for $C_{22}H_{22}N_8OS$: 446.1, Found: 446.1

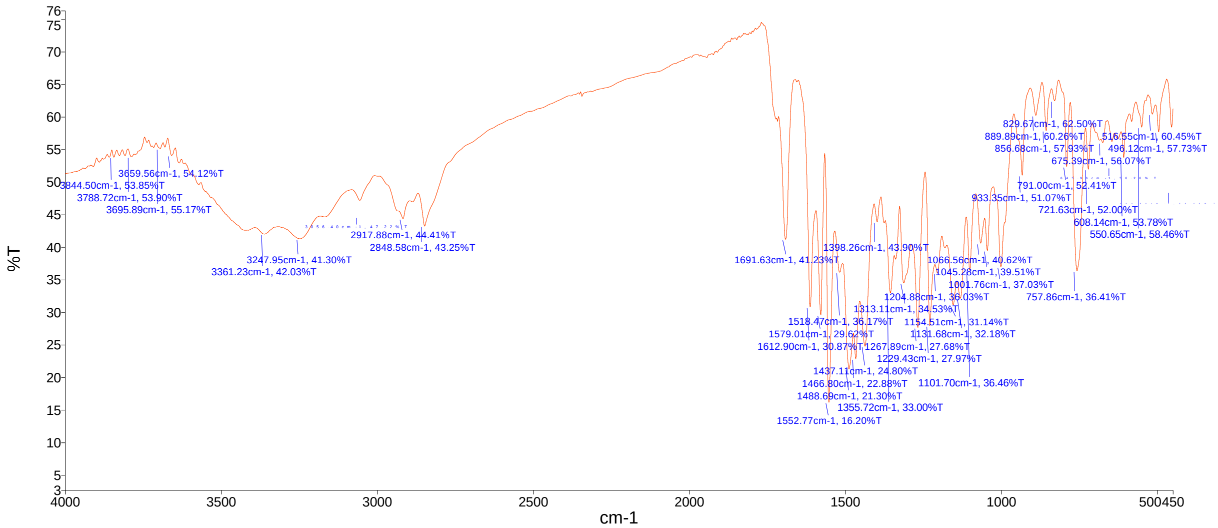

Fig S24. (FT-IR). 2-(2-oxo-1-((4-(quinoxalin-2-yl) piperazin-1-yl) methyl) indolin-3-ylidene) hydrazine-1-carbothioamide (3b)

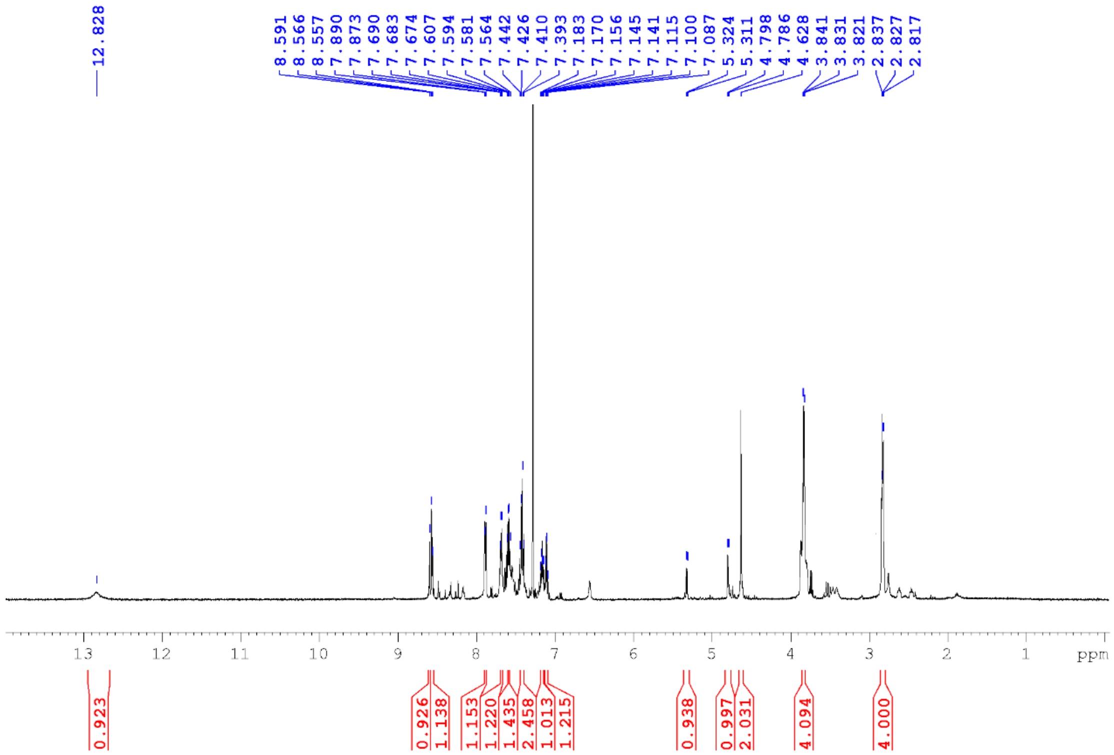

Fig S25. ^{1}HNMR Spectra. 2-(2-oxo-1-((4-(quinoxalin-2-yl) piperazin-1-yl) methyl) indolin-3-ylidene) hydrazine-1-carbothioamide (3b)

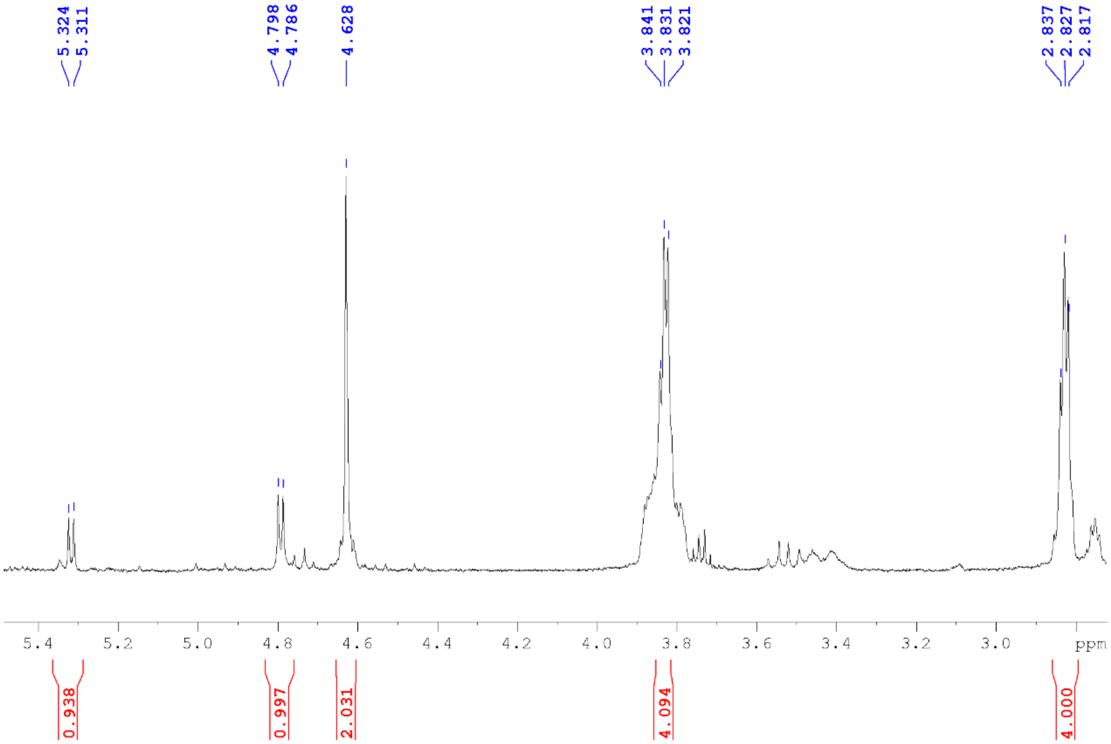

Fig S26. ^{1}HNMR Spectra. Expand- aliphatic region. 2-(2-oxo-1-((4-(quinoxalin-2-yl) piperazin-1-yl) methyl) indolin-3-ylidene) hydrazine-1-carbothioamide (3b)

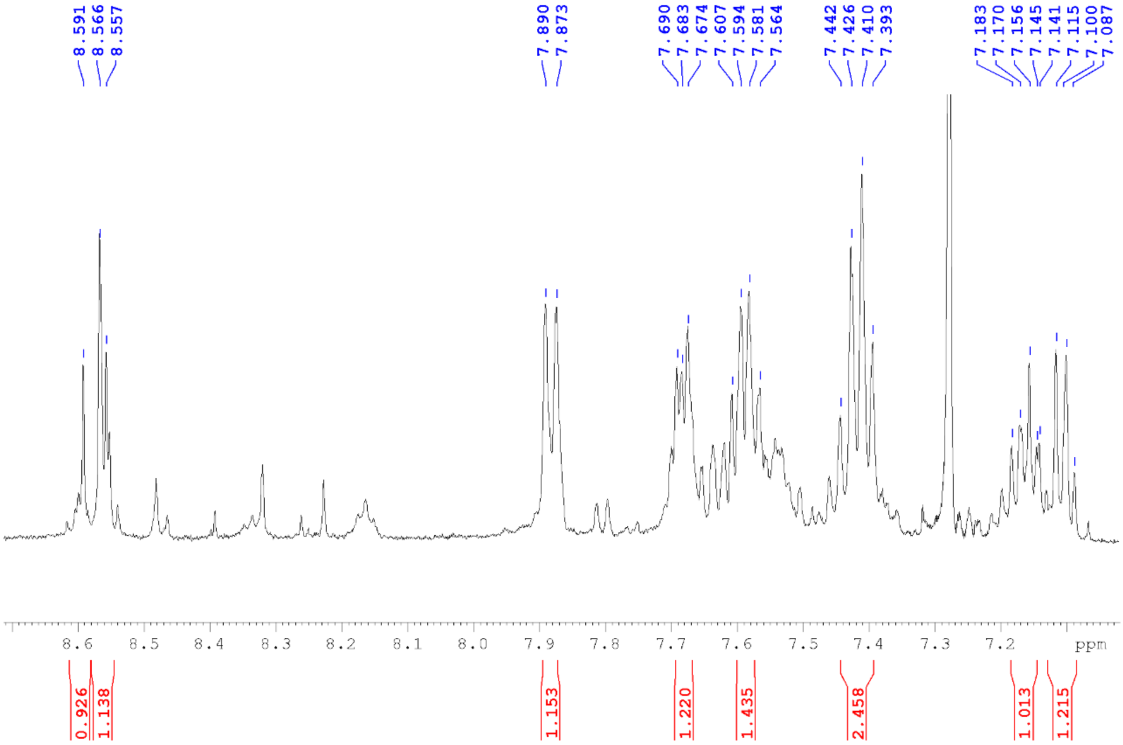

Fig S27. ^{1}HNMR Spectra. Expand- aromatic region. 2-(2-oxo-1-((4-(quinoxalin-2-yl) piperazin-1-yl) methyl) indolin-3-ylidene) hydrazine-1-carbothioamide (3b)

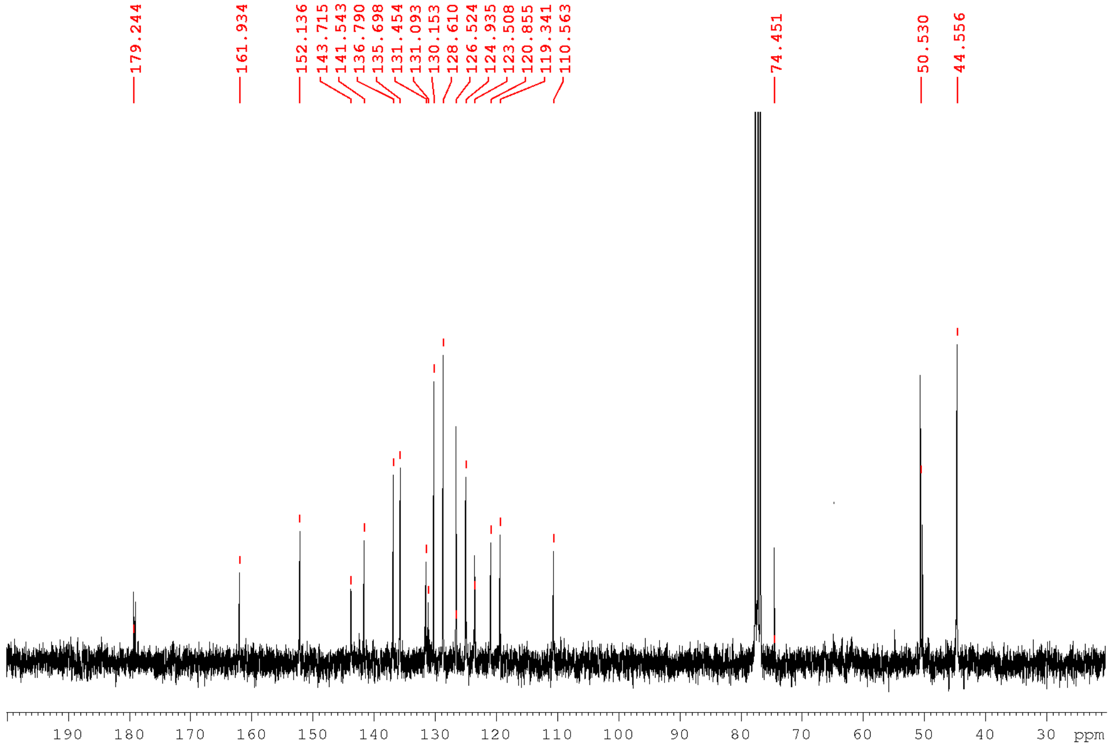

Fig S28. ^{13}CNMR Spectra. 2-(2-oxo-1-((4-(quinoxalin-2-yl) piperazin-1-yl) methyl) indolin-3-ylidene) hydrazine-1-carbothioamide (3b)

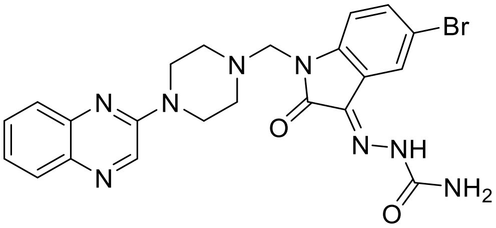

2-(5-bromo-2-oxo-1-((4-(quinoxalin-2-yl) piperazin-1-yl) methyl) indolin-3-ylidene) hydrazine-1-carboxamide (3c)

Orange crystals; IR (KBr, v_{max}, cm^{-1}): 3410 (N-H), 1699 (N-C=O), 1733 (N-C=O), 2798 (Ar-CH), 1457 (C=N), 1072 (C–N), 887 (C-Br) cm^{-1}; ^{1}H NMR (600 MHz, $CDCl_3$, DMSO) δ: 2.78-2.80 (m, 4H, b-2H, d-2H), 3.63-3.65 (d, j= 6, 1H, NH_2-H), 3.81-3.83 (m, 4H, a-2H, c-2H), 4.59 (s, 2H, CH_2), 4.90-4.91 (d, j= 6, 1H, NH_2-H), 7.00-7.01 (d, j= 8, 1H, 7-H), 7.40-7.41 (d, j= 8, 1H, 6-H),

7.43 (s, 1H, 4-H) 7.48-7.51 (m, 1H, 6'-H), 7.57-7.59 (m, 1H, 7'-H), 7.87-7.89 (d, j= 8.8, 1H, 5’-H), 8.55-8.56 (d, j= 8.8, 1H, 8'-H), 8.61 (s, 1H, 3'-H), 11.90 (s, 1H, NH); ^{13}C NMR (150 MHz, $CDCl_3$, DMSO) δ: 44.53 (2C), 50.43 (2C), 61.80, 111.98, 116.20, 121.43, 123.24, 124.99, 126.53, 128.66, 130.17, 133.12, 135.60, 136.87, 141.50, 141.67, 152.01, 154.12, 155.16(C=O), 161.64(C=O); MS: (m/z) M^+ Anal. calcd for $C_{22}H_{21}BrN_8O_2$: 508.1, Found: 508.1

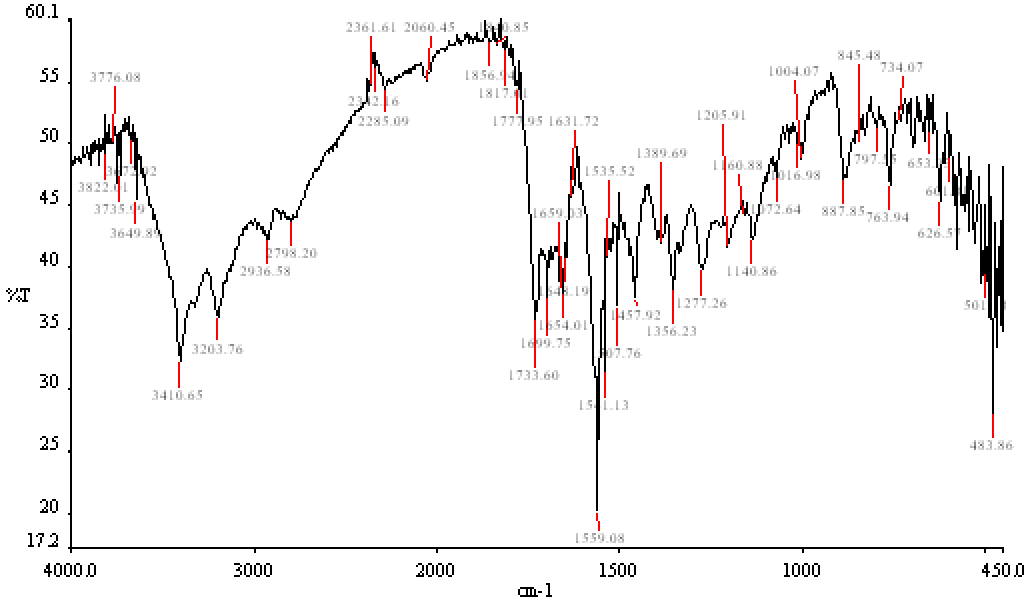

Fig S29. (FT-IR). 2-(5-bromo-2-oxo-1-((4-(quinoxalin-2-yl) piperazin-1-yl) methyl) indolin-3-ylidene) hydrazine-1-carboxamide (3c)

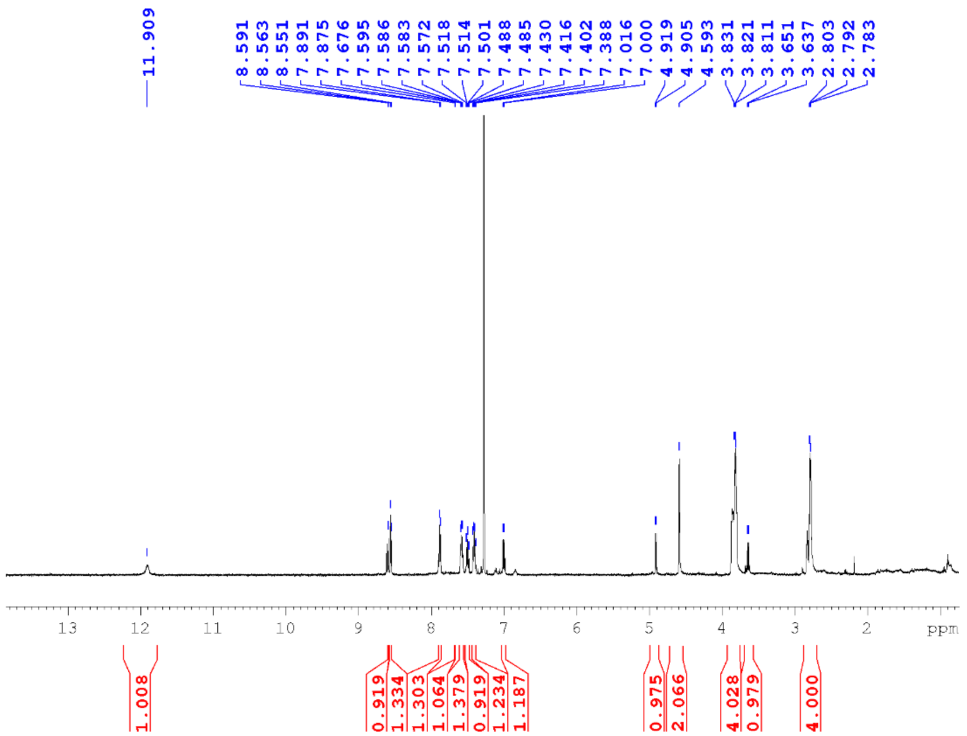

Fig S30. ^{1}HNMR Spectra. 2-(5-bromo-2-oxo-1-((4-(quinoxalin-2-yl) piperazin-1-yl) methyl) indolin-3-ylidene) hydrazine-1-carboxamide (3c)

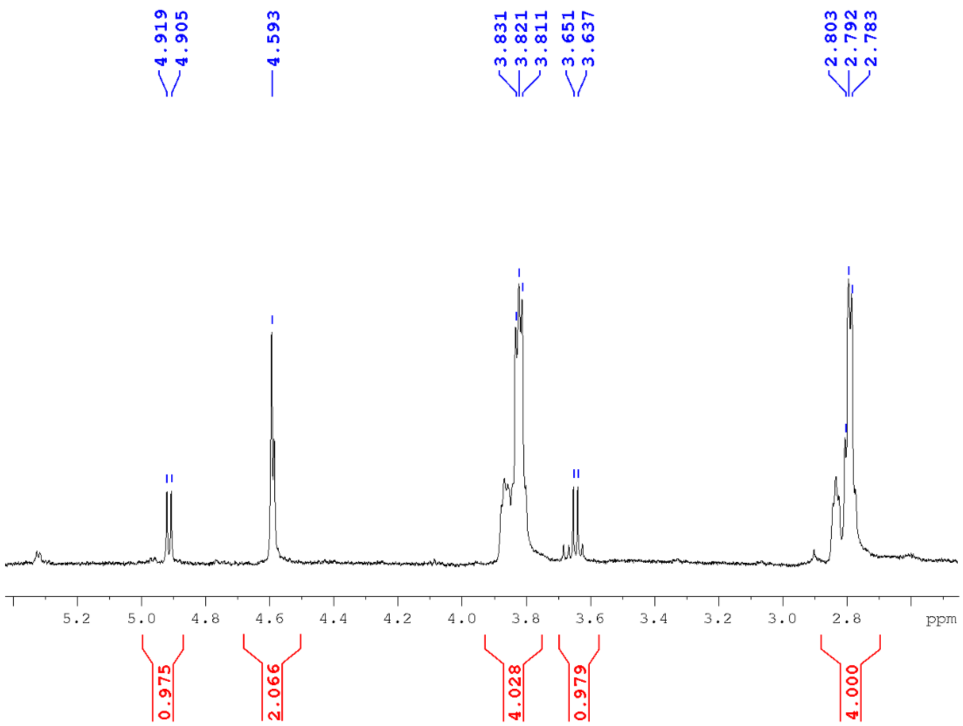

Fig S31. ¹HNMR Spectra. Expand- aliphatic region. 2-(5-bromo-2-oxo-1-((4-(quinoxalin-2-yl) piperazin-1-yl) methyl) indolin-3-ylidene) hydrazine-1-carboxamide (3c)

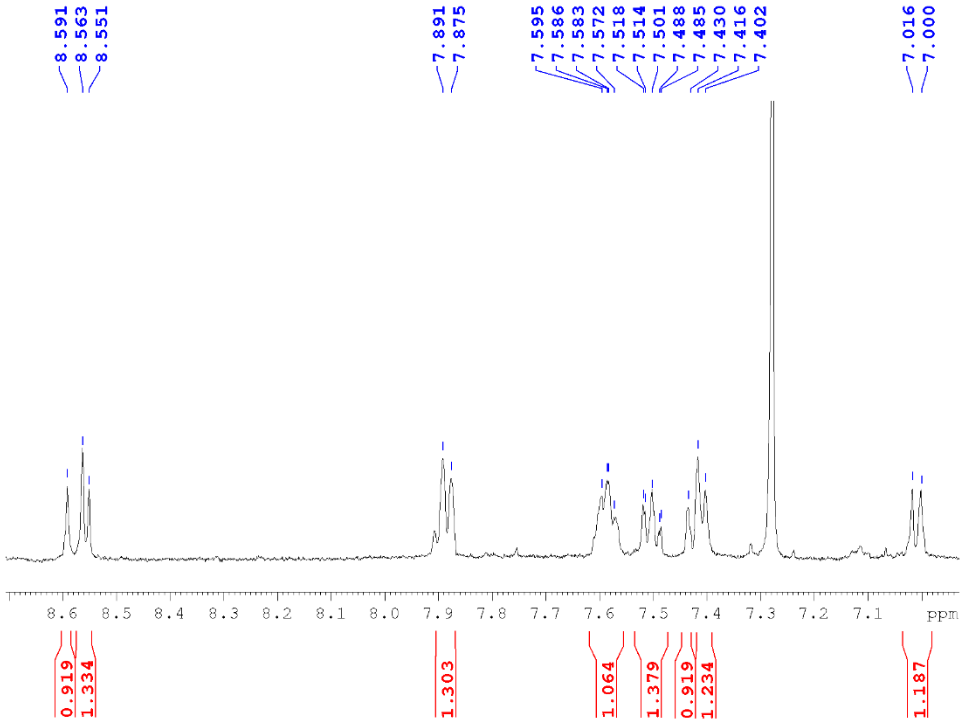

Fig S32. ^{1}HNMR Spectra. Expand- aromatic region. 2-(5-bromo-2-oxo-1-((4-(quinoxalin-2-yl) piperazin-1-yl) methyl) indolin-3-ylidene) hydrazine-1-carboxamide (3c)

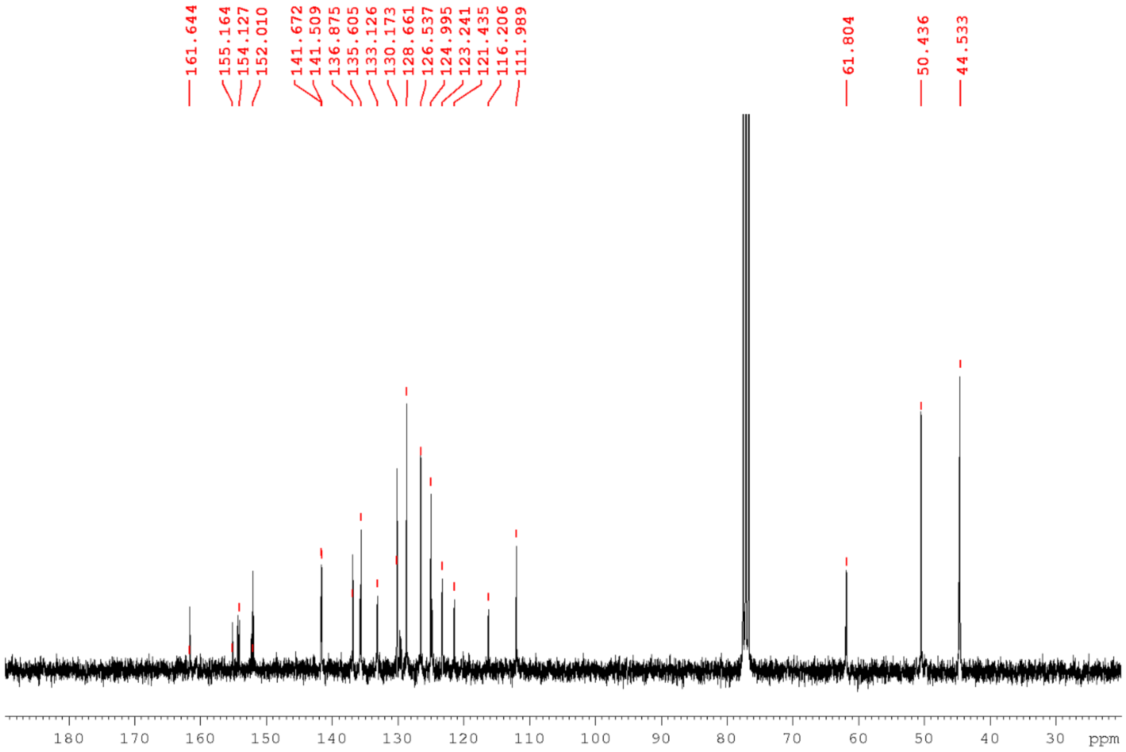

Fig S33. ^{13}CNMR Spectra. 2-(5-bromo-2-oxo-1-((4-(quinoxalin-2-yl) piperazin-1-yl) methyl) indolin-3-ylidene) hydrazine-1-carboxamide (3c)

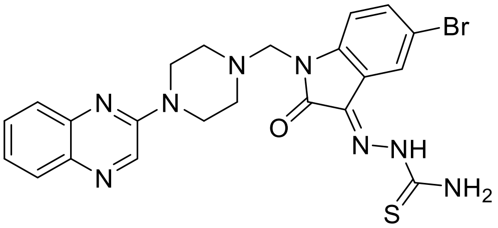

2-(5-bromo-2-oxo-1-((4-(quinoxalin-2-yl) piperazin-1-yl) methyl) indolin-3-ylidene) hydrazine-1-carbothioamide (3d)

Orange crystals; IR (KBr, v_{max}, cm^{-1}): 3440 (N-H), 1722 (N-C=O), 1634 (N-C=S), 2952 (Ar-CH), 1484 (C=N), 1024 (C–N), 867 (C-Br) cm^{-1}; ^{1}H NMR (600 MHz, $CDCl_3$, DMSO) δ: 2.78-2.80 (m, 4H, b-2H, d-2H), 3.71-3.73 (d, j= 6, 1H, NH_2-H), 3.81-3.83 (m, 4H, a-2H, c-2H), 4.60 (s, 2H, CH_2), 5.29-5.31 (d, j= 6, 1H, NH_2-H), 6.99-7.01 (d, j= 8, 1H, 7-H), 7.39-7.43 (m, 1H, 6'-H), 7.51-7.54 (m, 1H, 6-H), 7.56-7.60 (m, 1H, 7'-H), 7.67-7.69 (m, 1H, 5'-H), 7.76 (s, 1H, 4-H), 7.87-7.89

(m, 1H, 8'-H), 8.56 (s, 1H, 3'-H), 12.76 (s, 1H, NH); ^{13}C NMR (150 MHz, $CDCl_3$, DMSO) δ: 44.54 (2C), 50.48 (2C), 74.51, 112.14,116.40, 121.16, 123.68, 124.82, 125.00, 126.55, 128.66, 130.18, 133.74, 135.62, 136.89, 141.51, 142.29, 152.01, 161.43(C=O), 179.11(C=S); MS: (m/z) M^+ Anal. calcd for $C_{22}H_{21}BrN_8OS$: 524.0, Found: 524.0

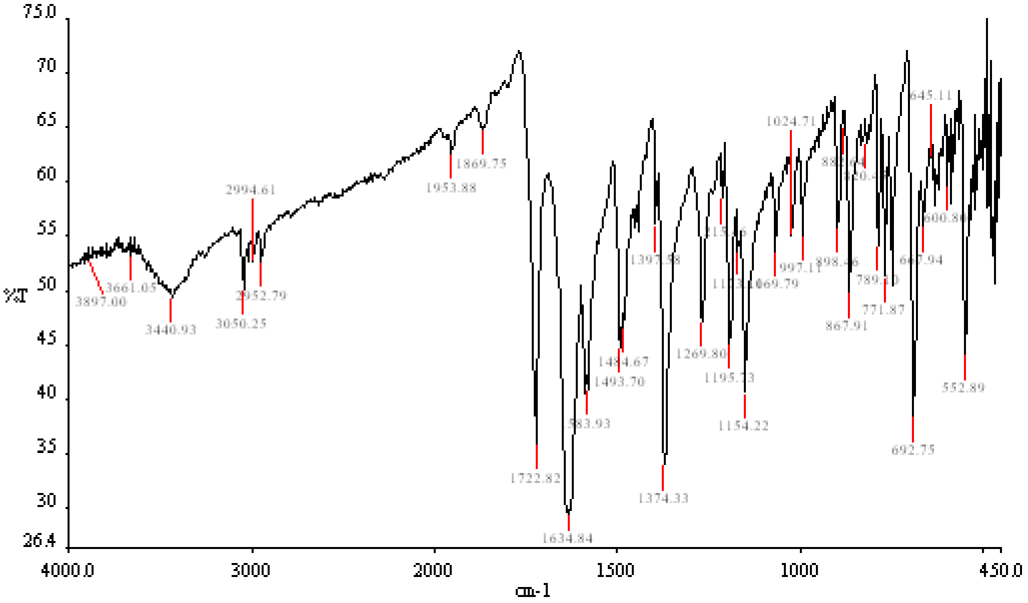

Fig S34. (FT-IR). 2-(5-bromo-2-oxo-1-((4-(quinoxalin-2-yl) piperazin-1-yl) methyl) indolin-3-ylidene) hydrazine-1-carbothioamide (3d)

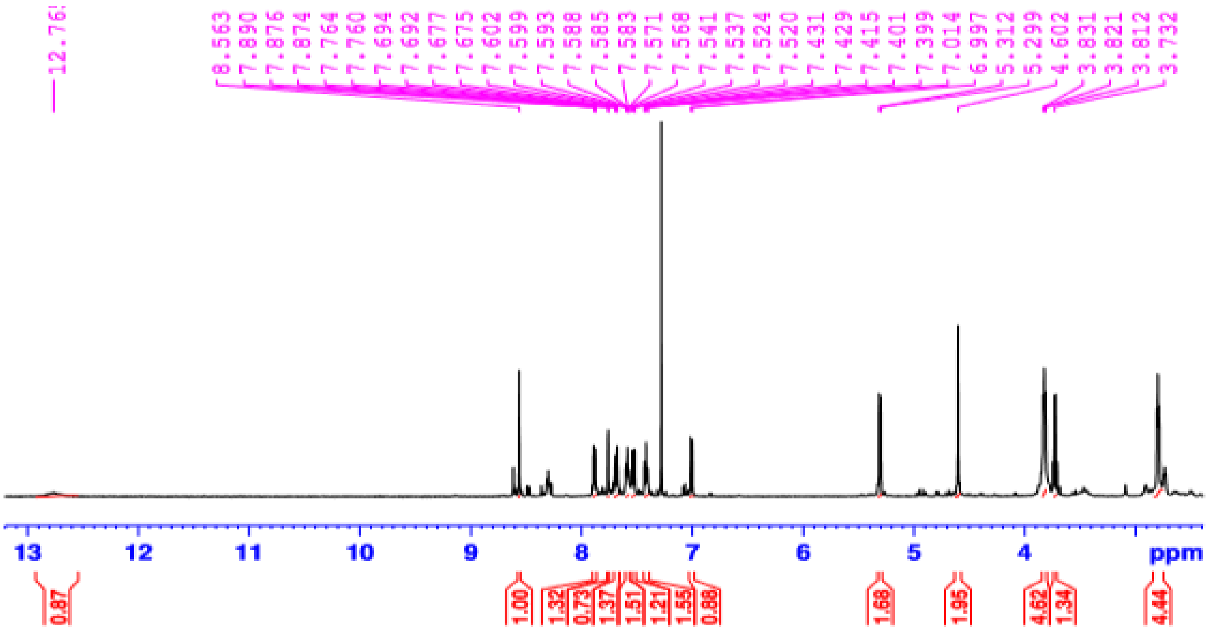

Fig S35. ^{1}HNMR Spectra. 2-(5-bromo-2-oxo-1-((4-(quinoxalin-2-yl) piperazin-1-yl) methyl) indolin-3-ylidene) hydrazine-1-carbothioamide (3d)

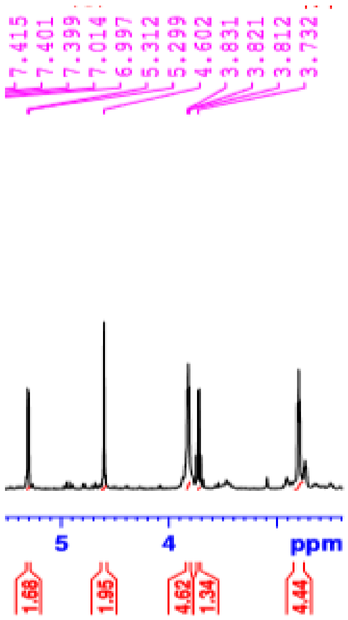

Fig S36. ^{1}HNMR Spectra. Expand- aliphatic region. 2-(5-bromo-2-oxo-1-((4-(quinoxalin-2-yl) piperazin-1-yl) methyl) indolin-3-ylidene) hydrazine-1-carbothioamide (3d)

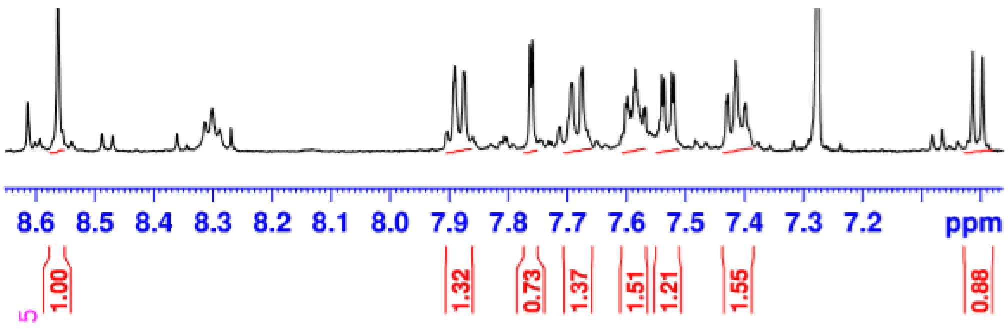

Fig S37. ^{1}HNMR Spectra. Expand- aromatic region. 2-(5-bromo-2-oxo-1-((4-(quinoxalin-2-yl) piperazin-1-yl) methyl) indolin-3-ylidene) hydrazine-1-carbothioamide (3d)

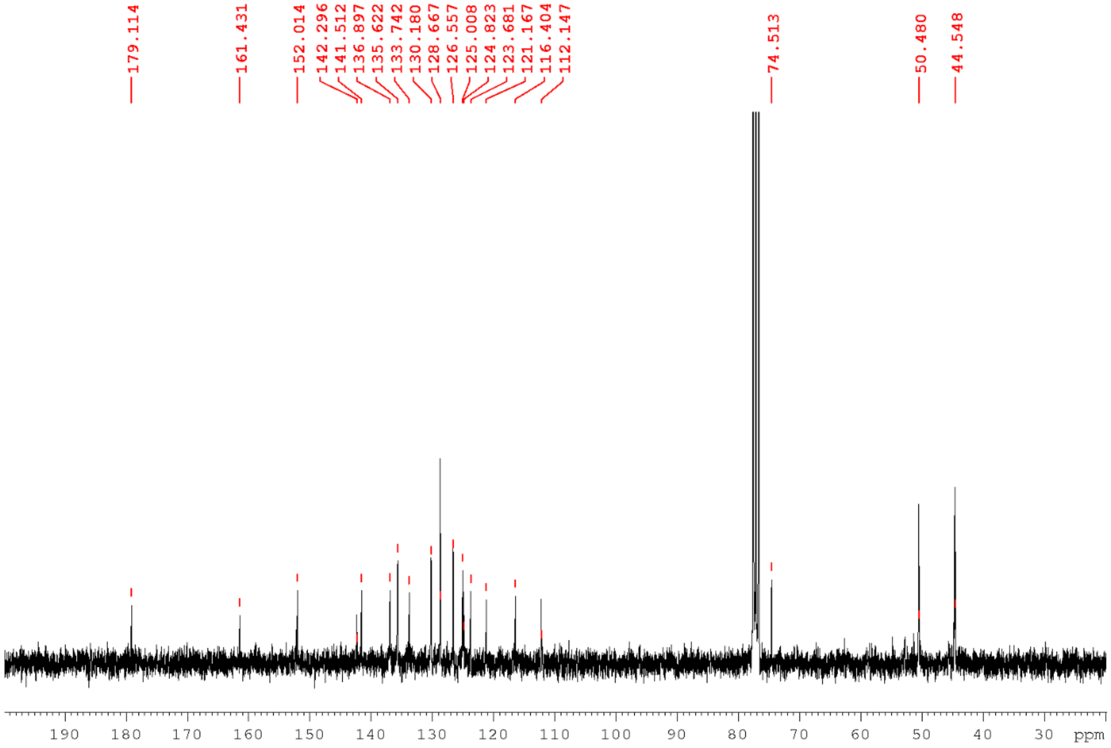

Fig S38. ^{13}CNMR Spectra. 2-(5-bromo-2-oxo-1-((4-(quinoxalin-2-yl) piperazin-1-yl) methyl) indolin-3-ylidene) hydrazine-1-carbothioamide (3d)

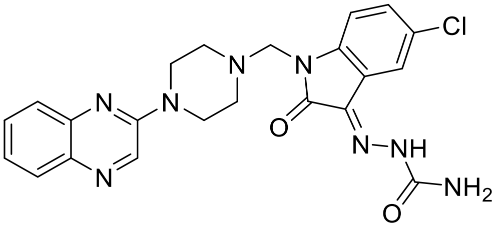

2-(5-chloro-2-oxo-1-((4-(quinoxalin-2-yl) piperazin-1-yl) methyl) indolin-3-ylidene) hydrazine-1-carboxamide (3e)

Orange crystals; IR (KBr, v_{max}, cm^{-1}): 3338 (N-H), 1722 (N-C=O), 1712 (N-C=O), 2775 (Ar-CH), 1411 (C=N), 1036 (C–N), 755 (C-Cl) cm^{-1}; ^{1}H NMR (600 MHz, $CDCl_3$, DMSO) δ: 2.78-2.81 (m, 4H, b-2H, d-2H), 3.64-3.67 (d, j= 6, 1H, NH_2-H), 3.81-3.84 (m, 4H, a-2H, c-2H), 4.59 (s, 2H, CH_2), 4.91-4.93 (d, j= 6, 1H, NH_2-H), 7.06 (s, 1H, 4-H), 7.34-7.35 (d, j= 8, 1H, 7-H), 7.36-7.37 (dd, j= 8, 6-H), 7.39-7.44 (m, 2H, 6'-H, 7'H), 7.56-7.61 (m, 1H, 5'-H), 7.67-7.70 (m, 1H, 8'-H),

8.57 (s, 1H, 3'-H), 11.91 (s, 1H, NH); ^{13}C NMR (150 MHz, $CDCl_3$, DMSO) δ: 44.54 (2C), 50.44 (2C), 70.70, 111.57, 120.45, 121.09, 125.00, 126.53, 128.63, 129.05, 130.18, 130.34, 135.60, 136.84, 141.24, 141.52, 152.02, 154.17, 155.32(C=O), 161.79(C=O); MS: (m/z) M^+ Anal. calcd for $C_{22}H_{21}ClN_8O_2$: 464.1, Found: 464.1

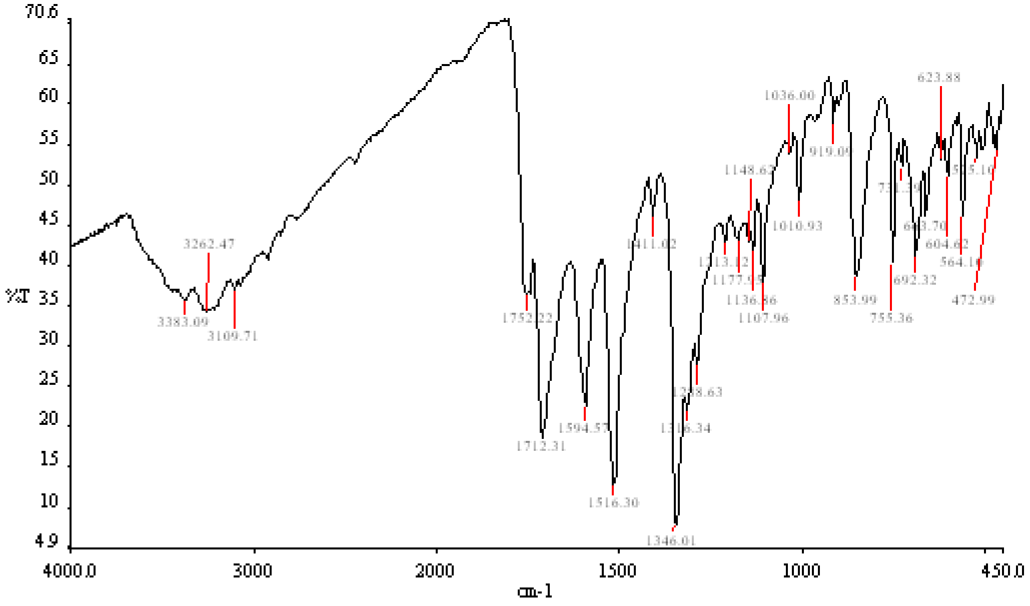

Fig S39. (FT-IR). 2-(5-chloro-2-oxo-1-((4-(quinoxalin-2-yl) piperazin-1-yl) methyl) indolin-3-ylidene) hydrazine-1-carboxamide (3e)

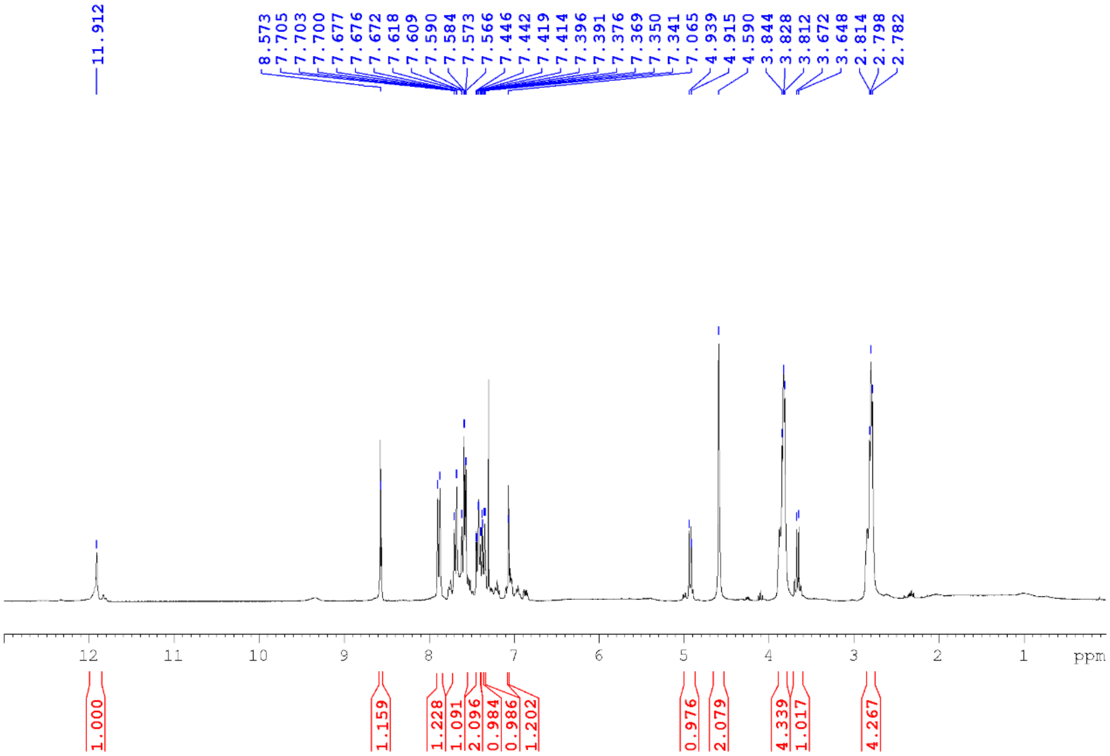

Fig S40. ¹HNMR Spectra. 2-(5-chloro-2-oxo-1-((4-(quinoxalin-2-yl) piperazin-1-yl) methyl) indolin-3-ylidene) hydrazine-1-carboxamide (3e)

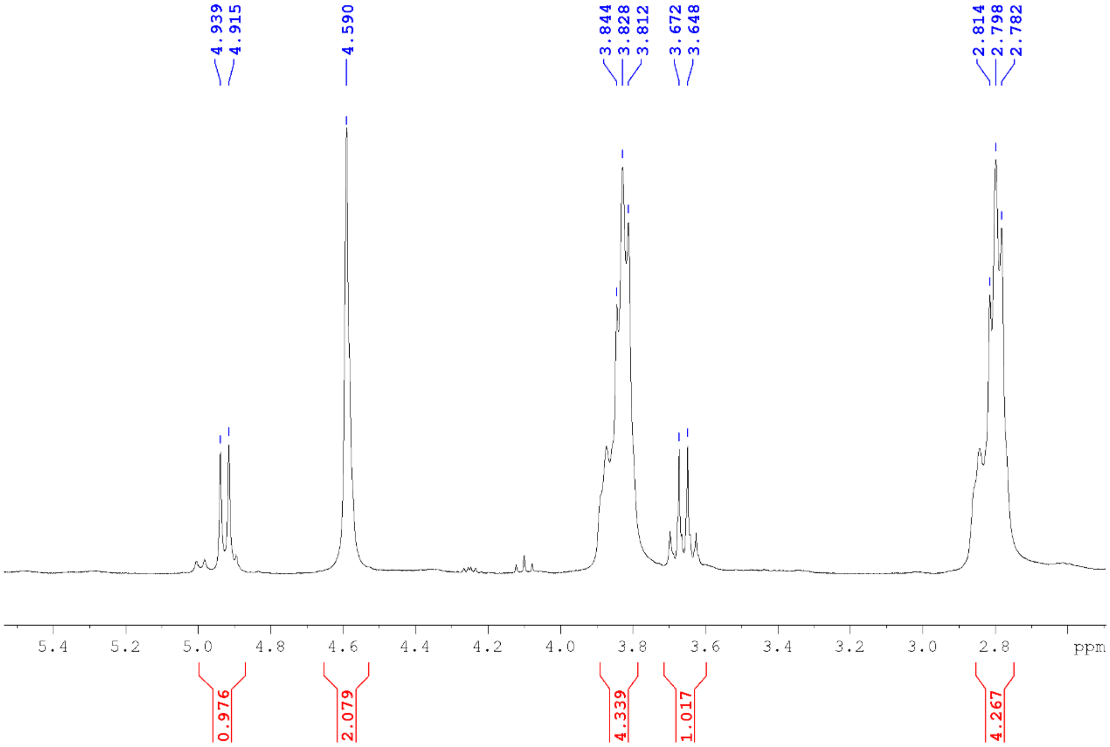

Fig S41. ¹HNMR Spectra. Expand- aliphatic region. 2-(5-chloro-2-oxo-1-((4-(quinoxalin-2-yl) piperazin-1-yl) methyl) indolin-3-ylidene) hydrazine-1-carboxamide (3e)

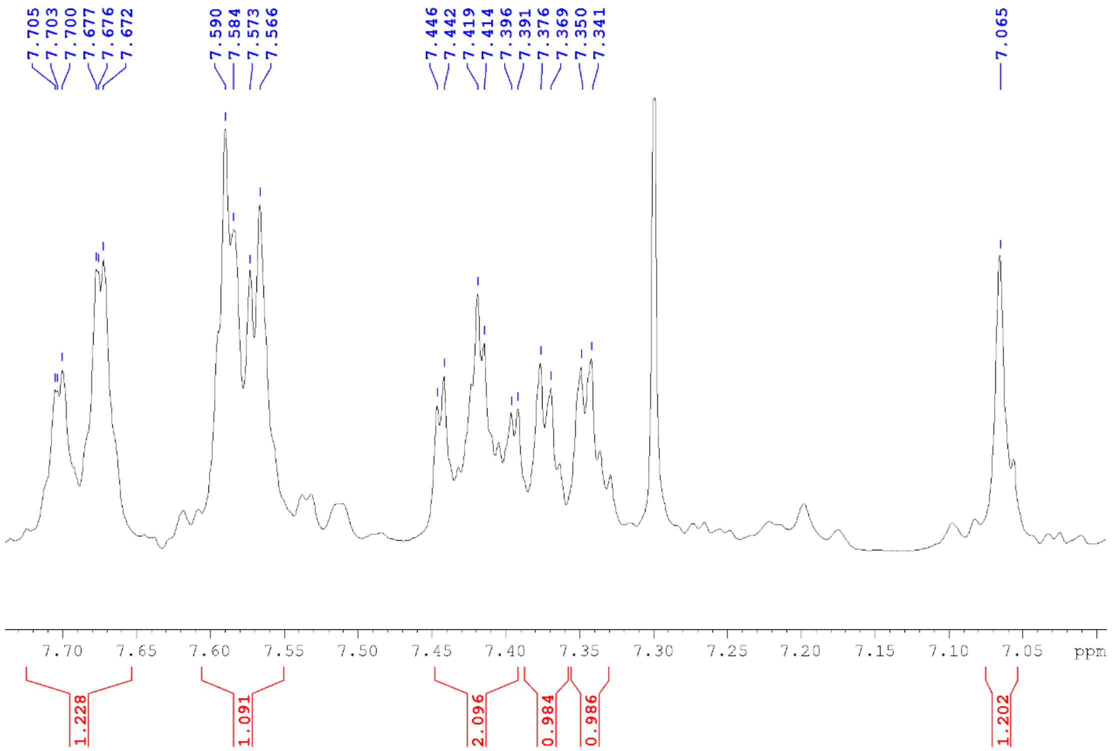

Fig S42. ¹HNMR Spectra. Expand- aromatic region. 2-(5-chloro-2-oxo-1-((4-(quinoxalin-2-yl) piperazin-1-yl) methyl) indolin-3-ylidene) hydrazine-1-carboxamide (3e)

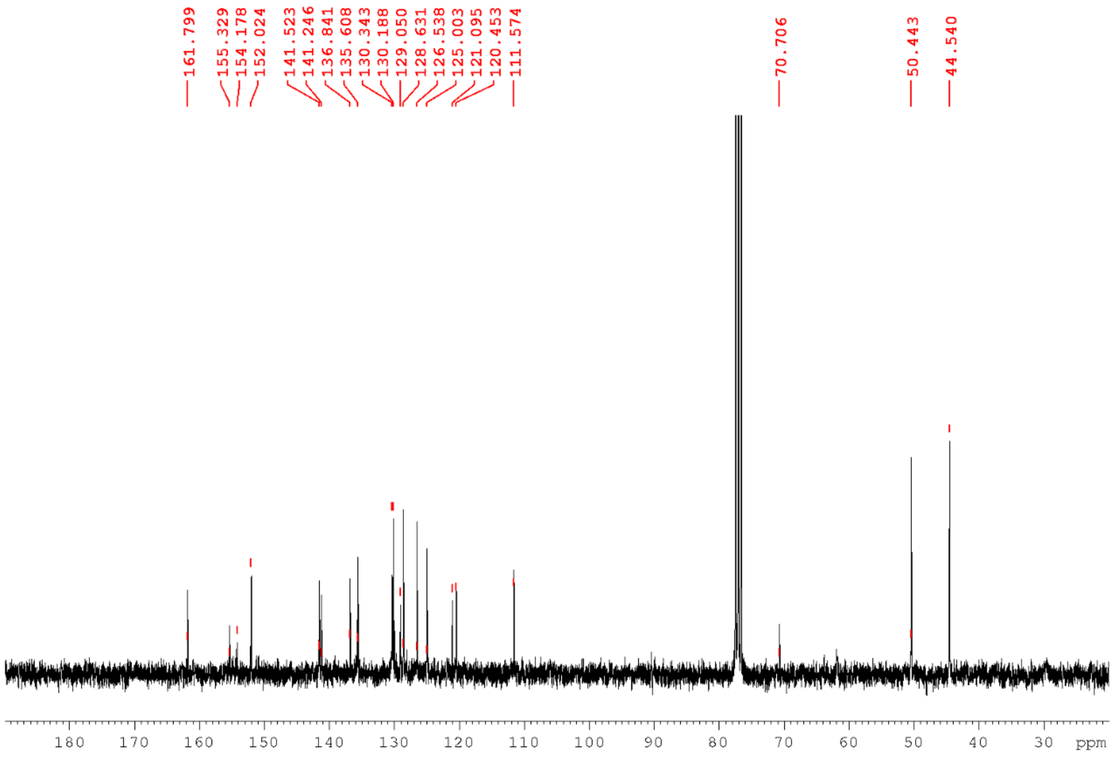

Fig S43. ^{13}CNMR Spectra. 2-(5-chloro-2-oxo-1-((4-(quinoxalin-2-yl) piperazin-1-yl) methyl) indolin-3-ylidene) hydrazine-1-carboxamide (3e)

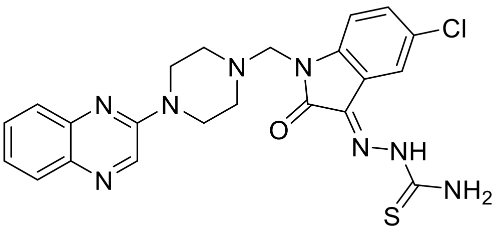

2-(5-chloro-2-oxo-1-((4-(quinoxalin-2-yl) piperazin-1-yl) methyl) indolin-3-ylidene) hydrazine-1-carbothioamide (3f)

Orange crystals; IR (KBr, v_{max}, cm^{-1}): 3447 (N-H), 1703 (N-C=O), 1602 (N-C=S), 2872 (Ar-CH), 1426 (C=N), 1033 (C–N), 762 (C-Cl) cm^{-1}; ^{1}H NMR (600 MHz, $CDCl_3$, DMSO) δ: 2.79-2.81 (m, 4H, b-2H, d-2H), 3.72-3.73 (d, j= 6, 1H, NH_2-H), 3.81-3.83 (m, 4H, a-2H, c-2H), 4.60 (s, 2H, CH_2), 5.30-5.31 (d, j= 6, 1H, NH_2-H), 6.78-6.88 (d, j= 8, 1H, 7-H), 7.04-7.06 (d, j= 8, 1H, 6-H), 7.35-7.39 (m, 1H, 6'-H), 7.58-7.61 (m, 1H, 7'-H), 7.61 (s, 1H, 4-H), 7.67-7.69 (d, j= 8.8, 1H, 5'-

H), 7.87-7.89 (d, j= 8.8, 1H, 8'-H), 8.56 (s, 1H, 3'-H), 12.79 (s, 1H, NH); ^{13}C NMR (150 MHz, $CDCl_3$, DMSO) δ: 45.95 (2C), 50.04 (2C), 74.68, 112.11, 117.31, 124.17, 126.09, 127.25, 129.37, 129.79, 133.35, 134.79, 136.64, 137.80, 139.93, 148.77, 150.28, 153.08, 159.73(C=O), 184.26(C=S); MS: (m/z) M^+ Anal. calcd for $C_{22}H_{21}ClN_8OS$: 480.1, Found: 480.1

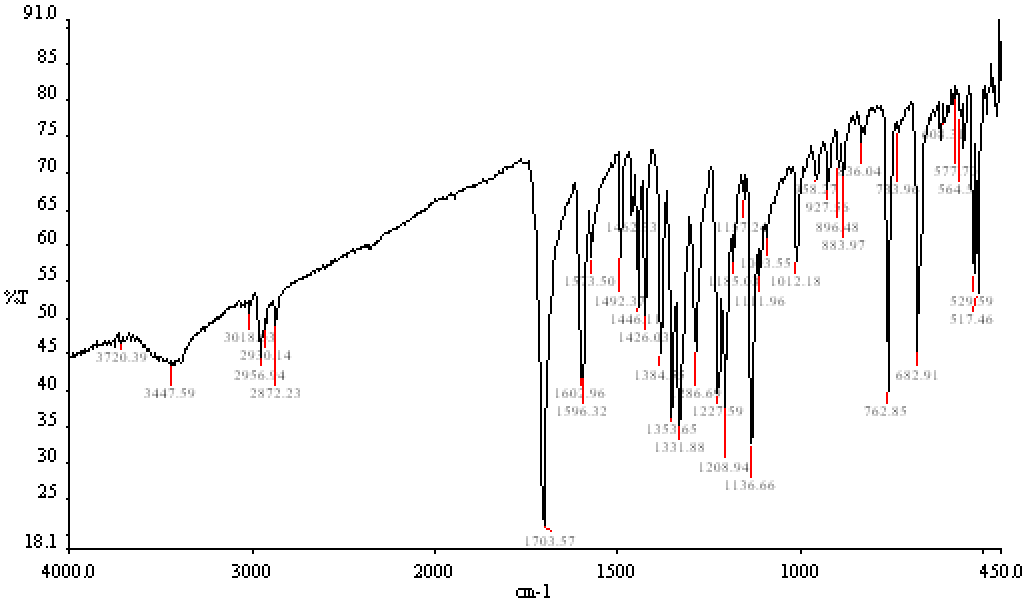

Fig S44. (FT-IR). 2-(5-chloro-2-oxo-1-((4-(quinoxalin-2-yl) piperazin-1-yl) methyl) indolin-3-ylidene) hydrazine-1-carbothioamide (3f)

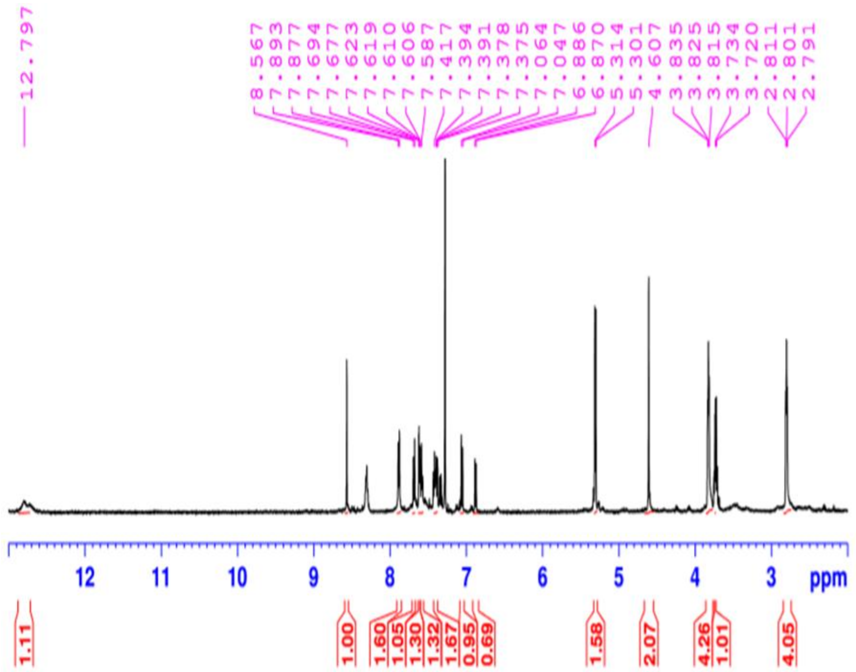

Fig S45. ¹HNMR Spectra. 2-(5-chloro-2-oxo-1-((4-(quinoxalin-2-yl) piperazin-1-yl) methyl) indolin-3-ylidene) hydrazine-1-carbothioamide (3f)

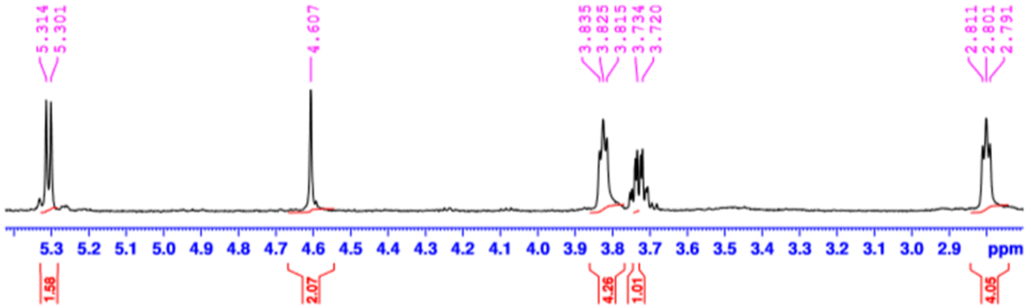

Fig S46. ¹HNMR Spectra. Expand- aliphatic region. 2-(5-chloro-2-oxo-1-((4-(quinoxalin-2-yl) piperazin-1-yl) methyl) indolin-3-ylidene) hydrazine-1-carbothioamide (3f)

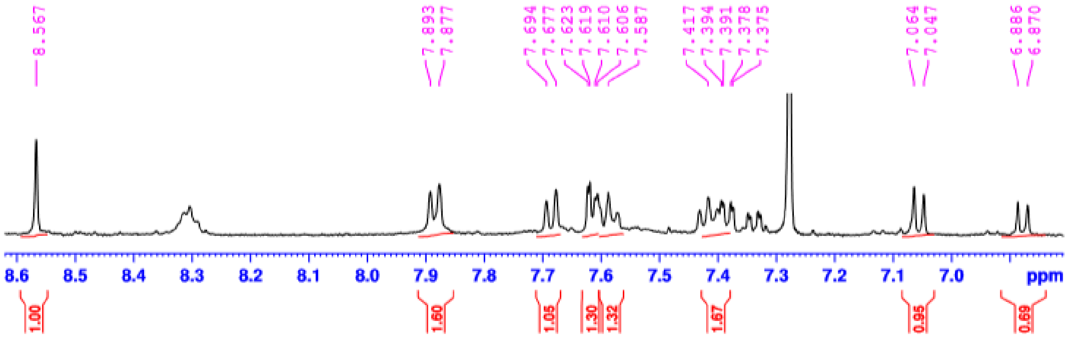

Fig S47. ^{1}HNMR Spectra. Expand- aromatic region. 2-(5-chloro-2-oxo-1-((4-(quinoxalin-2-yl) piperazin-1-yl) methyl) indolin-3-ylidene) hydrazine-1-carbothioamide (3f)

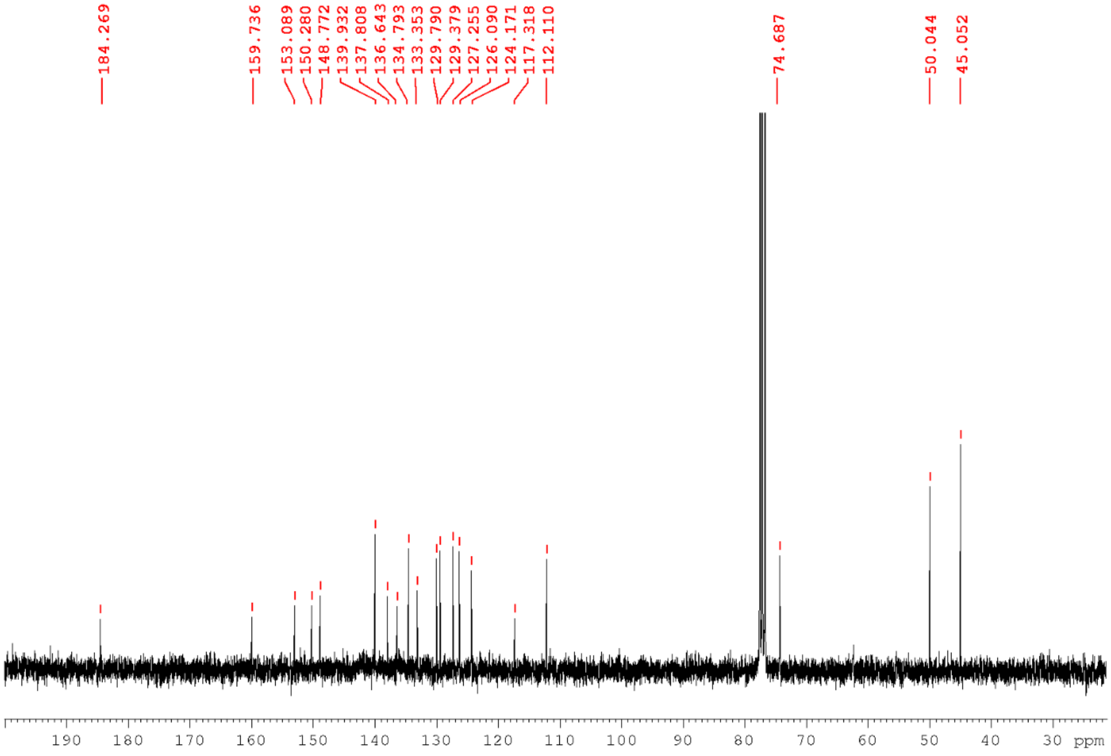

Fig S48. ^{13}CNMR Spectra. 2-(5-chloro-2-oxo-1-((4-(quinoxalin-2-yl) piperazin-1-yl) methyl) indolin-3-ylidene) hydrazine-1-carbothioamide (3f)

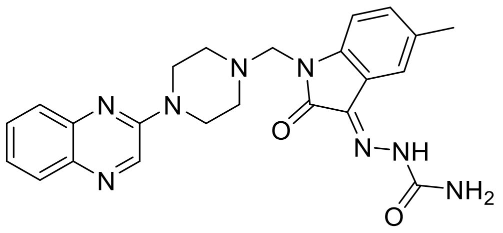

2-(5-methyl-2-oxo-1-((4-(quinoxalin-2-yl)piperazin-1-yl)methyl)indolin-3-ylidene)hydrazine-1-carboxamide (3g)

Orange crystals; IR (KBr, v_{max}, cm^{-1}): 3249 (N-H), 1685 (N-C=O), 1624 (N-C=O), 2922 (Ar-CH), 1431 (C=N), 1016 (C–N) cm^{-1}; ^{1}H NMR (600 MHz, $CDCl_3$, DMSO) δ: 2.33-2.38 (m, 4H, b-2H, d-2H), 2.81 (s, 3H, CH_3), 3.65-3.69 (m, 4H, a-2H, c-2H), 4.59 (s, 2H, CH_2), 4.91-4.93 (d, j= 6, 1H, NH_2-H), 5.27-5.29 (d, j= 6, 1H, NH_2-H), 6.97-7.00 (d, j= 8, 1H, 6-H), 7.18-7.21 (d, j= 8, 1H, 7-H), 7.36 (s, 1H, 4-H), 7.41-7.43 (m, 1H, 6'-H), 7.55-7.60 (m, 1H, 7'-H), 7.66-7.69 (d, j= 8.8, 1H, 5'-H), 7.86-7.89 (d, j= 8.8, 1H, 8'-H), 8.56 (s, 1H, 3'-H), 11.94 (s, 1H, NH); ^{13}C NMR (150 MHz, $CDCl_3$, DMSO) δ: 21.05, 44.55 (2C), 50.49 (2C), 70.65, 110.13, 119.63, 120.94, 124.92, 126.51, 128.58, 130.15, 131.30, 132.51, 133.06, 135.62, 136.75, 140.93, 141.56, 152.04, 154.68(C=O), 162.25(C=O); MS: (m/z) M^+ Anal. calcd for $C_{23}H_{24}N_8O_2$: 444.2, Found: 444.2

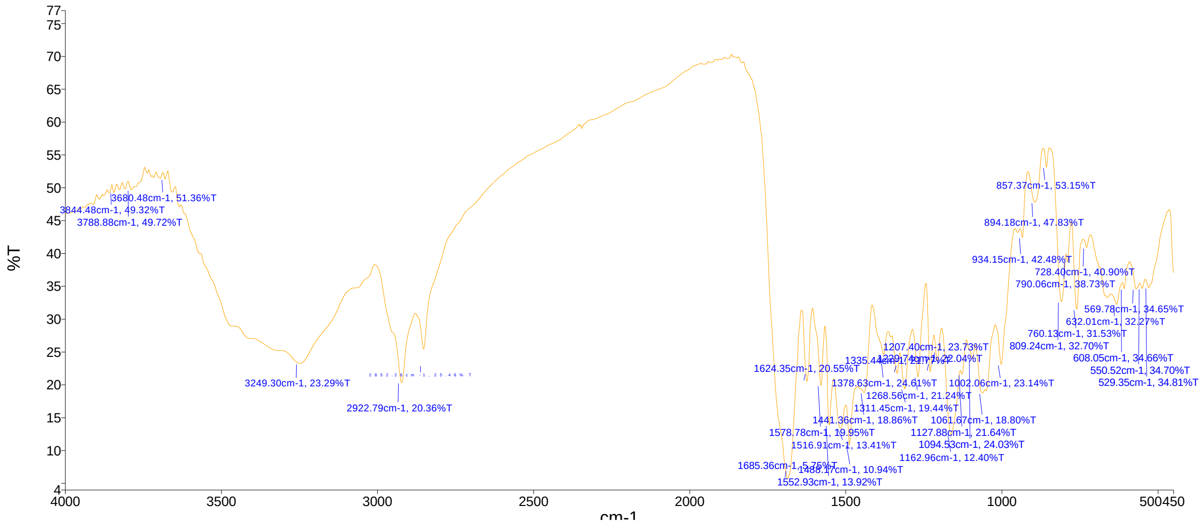

Fig S49. (FT-IR). 2-(5-methyl-2-oxo-1-((4-(quinoxalin-2-yl)piperazin-1-yl)methyl)indolin-3-ylidene)hydrazine-1-carboxamide (3g)

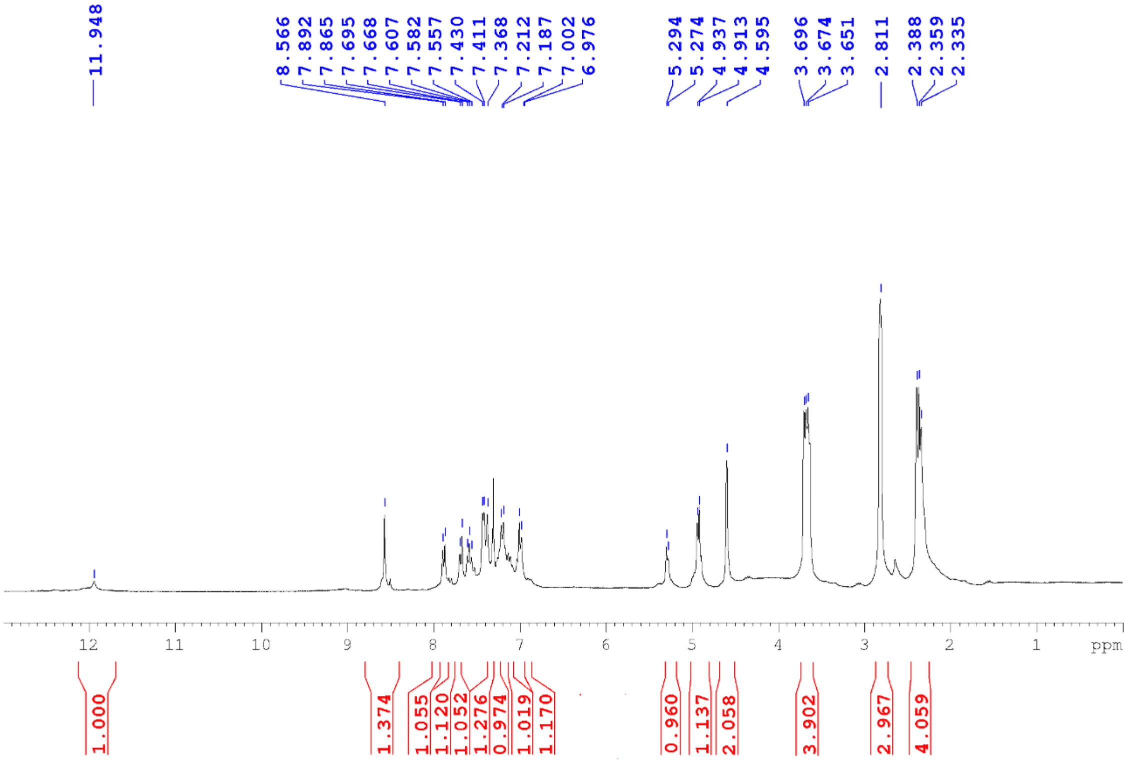

Fig S50. ^{1}HNMR Spectra. 2-(5-methyl-2-oxo-1-((4-(quinoxalin-2-yl)piperazin-1-yl)methyl)indolin-3-ylidene)hydrazine-1-carboxamide (3g)

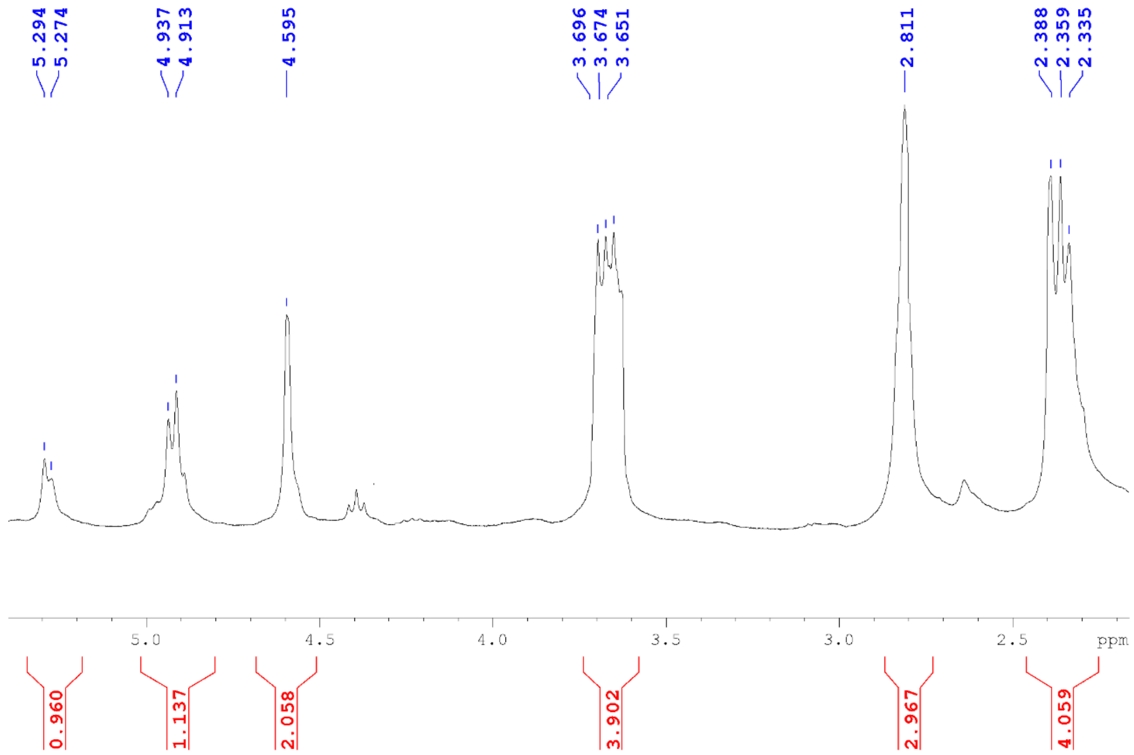

Fig S51. ^{1}HNMR Spectra. Expand- aliphatic region. 2-(5-methyl-2-oxo-1-((4-(quinoxalin-2-yl)piperazin-1-yl)methyl)indolin-3-ylidene)hydrazine-1-carboxamide (3g)

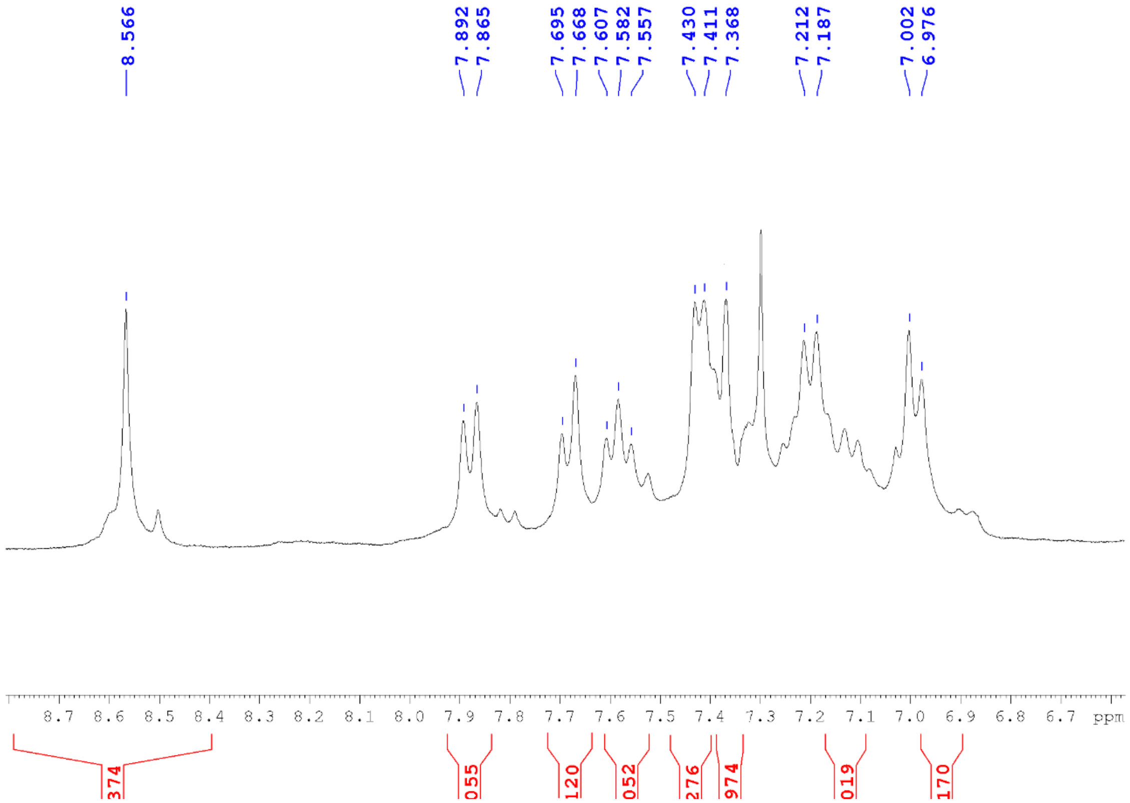

Fig S52. ^{1}HNMR Spectra. Expand- aromatic region. 2-(5-methyl-2-oxo-1-((4-(quinoxalin-2-yl)piperazin-1-yl)methyl)indolin-3-ylidene)hydrazine-1-carboxamide (3g)

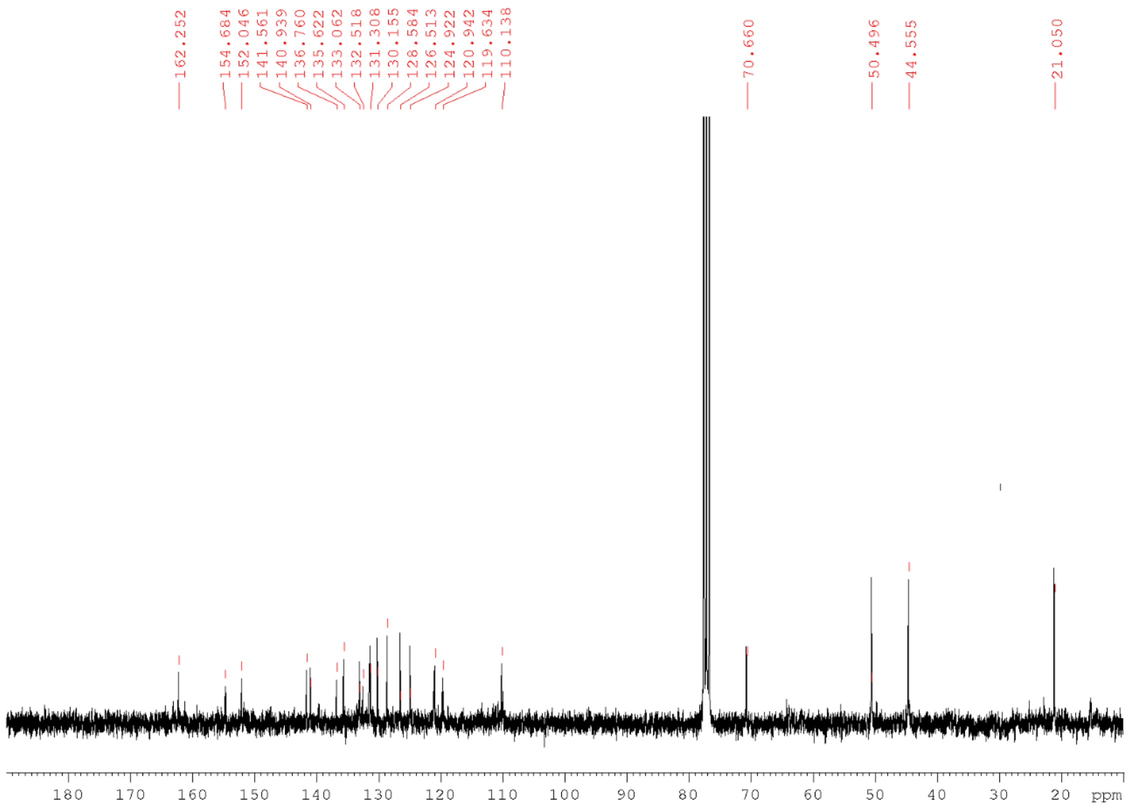

Fig S53. ^{13}CNMR Spectra. 2-(5-methyl-2-oxo-1-((4-(quinoxalin-2-yl)piperazin-1-yl)methyl)indolin-3-ylidene)hydrazine-1-carboxamide (3g)

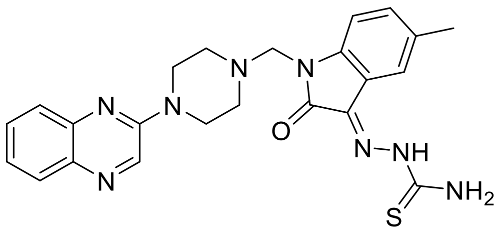

2-(5-methyl-2-oxo-1-((4-(quinoxalin-2-yl) piperazin-1-yl) methyl) indolin-3-ylidene) hydrazine-1-carbothioamide (3h)

Orange crystals; IR (KBr, v_{max}, cm^{-1}): 3325 (N-H), 1685 (N-C=O), 1624 (N-C=S), 2794 (Ar-CH), 1425 (C=N), 1018 (C–N) cm^{-1}; ^{1}H NMR (600 MHz, $CDCl_3$, DMSO) δ: 2.39 (s, 3H, CH_3), 2.80-

2.82 (m, 4H, b-2H, d-2H), 3.72-3.74 (d, j= 6, 1H, NH_2-H), 3.81-3.83 (m, 4H, a-2H, c-2H), 4.60 (s, 2H, CH_2), 5.30-5.32 (d, j= 6, 1H, NH_2-H), 6.98-6.99 (d, j= 8, 1H, 6-H), 7.21-7.23 (d, j= 8, 1H, 7-H), 7.39-7.40 (m, 1H, 6'-H), 7.45 (s, 1H, 4-H), 7.56-7.59 (m, 1H, 7'-H), 7.67-7.68 (d, j= 8.8, 1H, 5'-H), 7.87-7.88 (d, j= 8.8, 1H, 8'-H), 8.56 (s, 1H, 3'-H), 12.84 (s, 1H, NH) ; ^{13}C NMR (150 MHz, $CDCl_3$, DMSO) δ: 21.47, 43.35 (2C), 49.96 (2C), 69.16, 112.08, 118.16, 124.44, 125.27, 125.96, 127.90, 129.48, 130.87, 134.80, 136.32, 137.63, 140.81, 142.61, 150.55, 151.86, 153.38(C=O), 164.71(C= S); MS: (m/z) M^+ Anal. calcd for $C_{23}H_{24}N_8OS$: 460.1, Found: 460.1

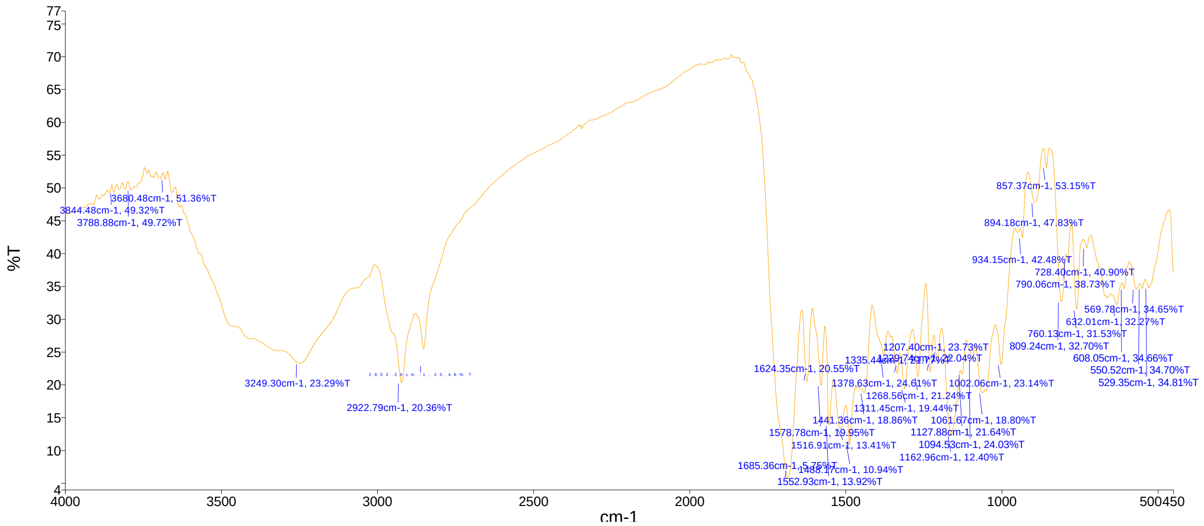

Fig S54. (FT-IR). 2-(5-methyl-2-oxo-1-((4-(quinoxalin-2-yl) piperazin-1-yl) methyl) indolin-3-ylidene) hydrazine-1-carbothioamide (3h)

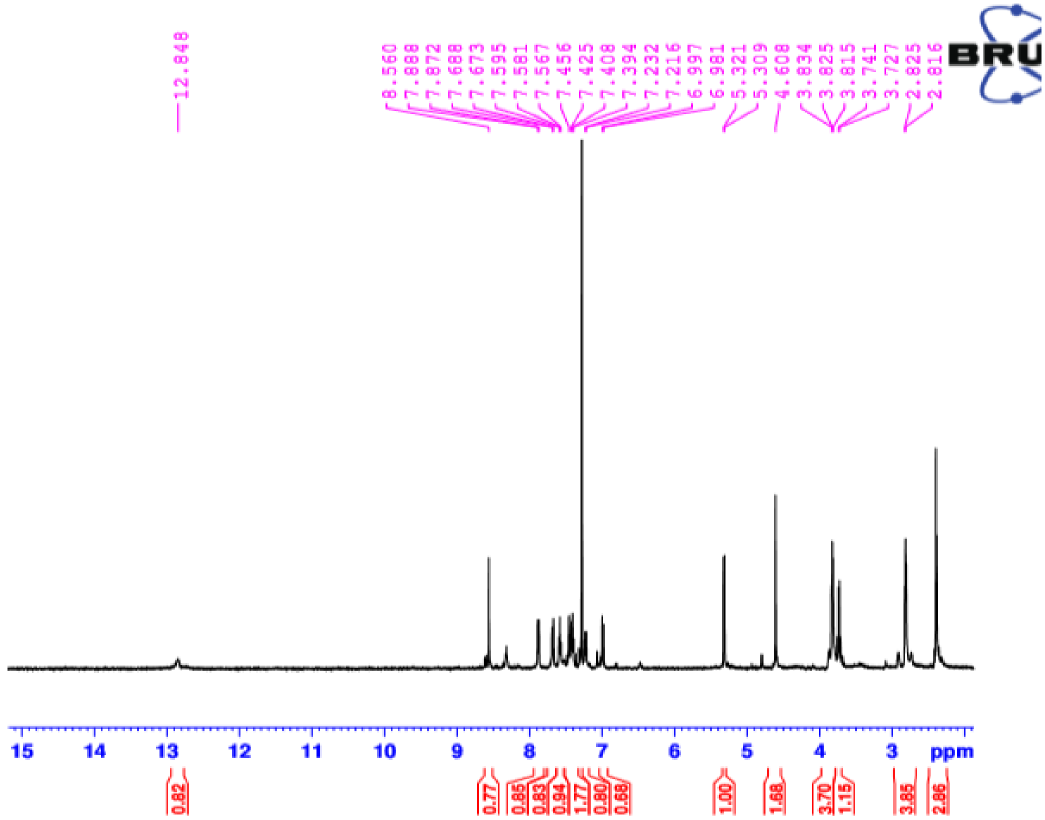

Fig S55. ^{1}HNMR Spectra. 2-(5-methyl-2-oxo-1-((4-(quinoxalin-2-yl) piperazin-1-yl) methyl) indolin-3-ylidene) hydrazine-1-carbothioamide (3h)

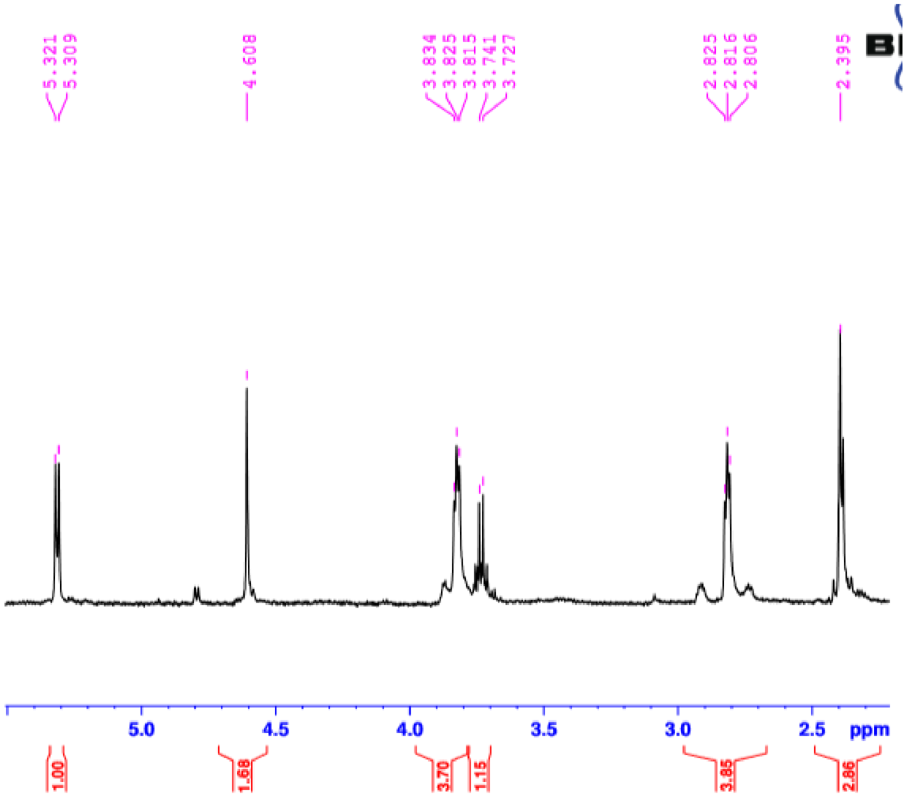

Fig S56. ^{1}HNMR Spectra. Expand- aliphatic region. 2-(5-methyl-2-oxo-1-((4-(quinoxalin-2-yl) piperazin-1-yl) methyl) indolin-3-ylidene) hydrazine-1-carbothioamide (3h)

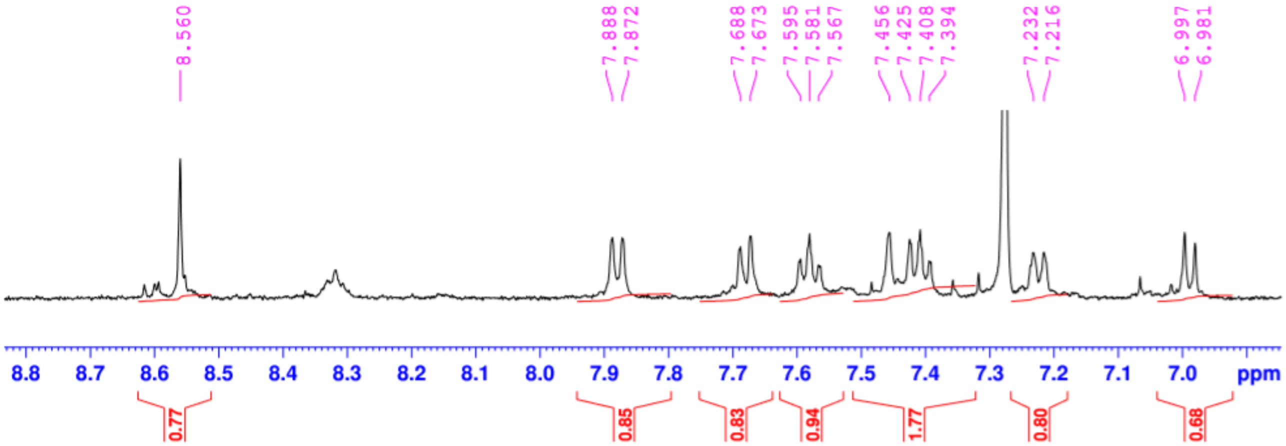

Fig S57. ^{1}HNMR Spectra. Expand- aromatic region. 2-(5-methyl-2-oxo-1-((4-(quinoxalin-2-yl) piperazin-1-yl) methyl) indolin-3-ylidene) hydrazine-1-carbothioamide (3h)

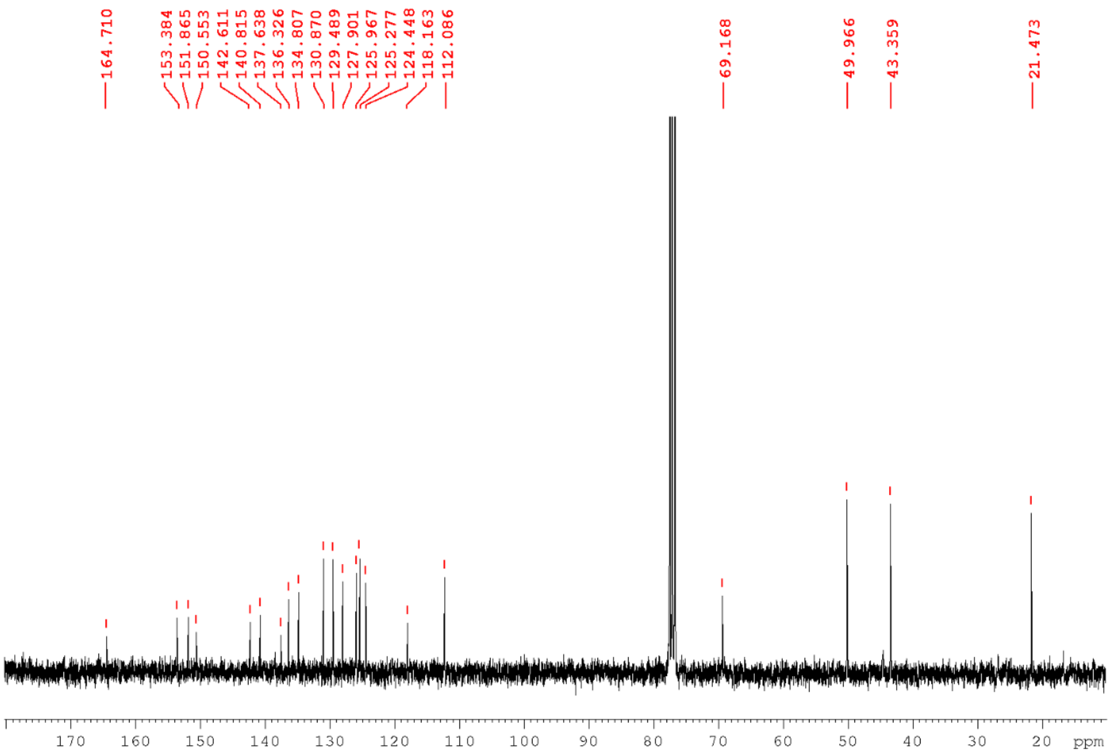

Fig S58. ^{13}CNMR Spectra. 2-(5-methyl-2-oxo-1-((4-(quinoxalin-2-yl) piperazin-1-yl) methyl) indolin-3-ylidene) hydrazine-1-carbothioamide (3h)

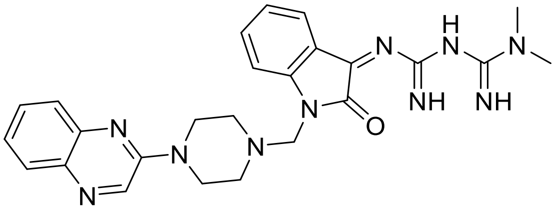

N'-[4-(quinoxaline-2-yl)-piperazine-1-yl]methyl-1-H-indole,2,3-dion-3-metformin (4a)

Dark Orange crystals; IR (KBr, v_{max}, cm^{-1}): 3424, 3064 (N-H), 1695 (N-C=O), 2526 (Ar-CH), 1430 (C=N), 1020 (C–N) cm^{-1}; ^{1}H NMR (600 MHz, $CDCl_3$, DMSO) δ: 2.40 (s, 6H, CH_3, CH_3), 2.69-2.71 (m, 4H, b-2H, d-2H), 3.25-3.27 (m, 4H, a-2H, c-2H), 4.23 (s, 2H, CH_2), 7.09- 7.13 (m, 2H, 5-H, 6-H), 7.32- 7.34 (m, 2H, 4-H, 7-H), 7.46- 7.49 (m, 2H, 6'-H, 7'-H), 7.63-7.65 (d, j= 8.8,

1H, 5'-H), 8.06-8.08 (d, j= 8.8, 1H, 8'-H), 8.48 (s, 1H, 3'-H), 8.59 (s, 1H, C=NH), 8.76 (s, 1H, C=NH), 9.98 (s, 1H, NH); ^{13}C NMR (150 MHz, $CDCl_3$, DMSO) δ: 38.40 (2C), 47.48 (2C), 55.74 (2C), 114.40, 116.148, 118.47, 121.34, 124.67, 125.56, 128.34, 133.34, 135.14, 136.39, 138.37, 139.56, 144.57, 146.27, 156.36, 156.50(C=NH), 166.88(C=NH), 167.61(C=O); MS: (m/z) M^+ Anal. calcd for $C_{25}H_{28}N_{10}O$: 484.2, Found: 484.2

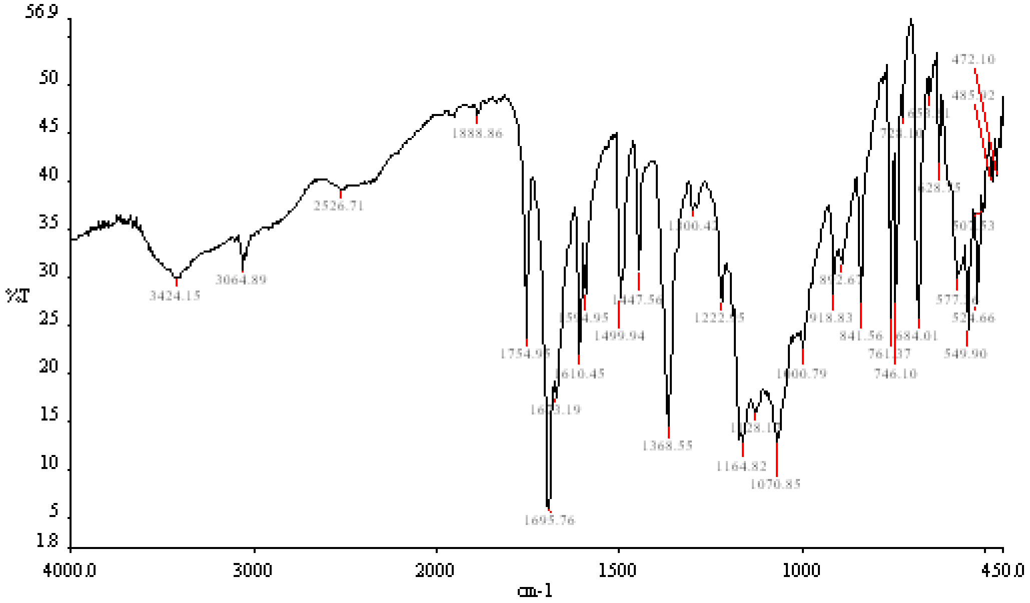

Fig S59. (FT-IR). N'-[4-(quinoxaline-2-yl)-piperazine-1-yl]methyl-1-H-indole,2,3-dion-3-metformin (4a)

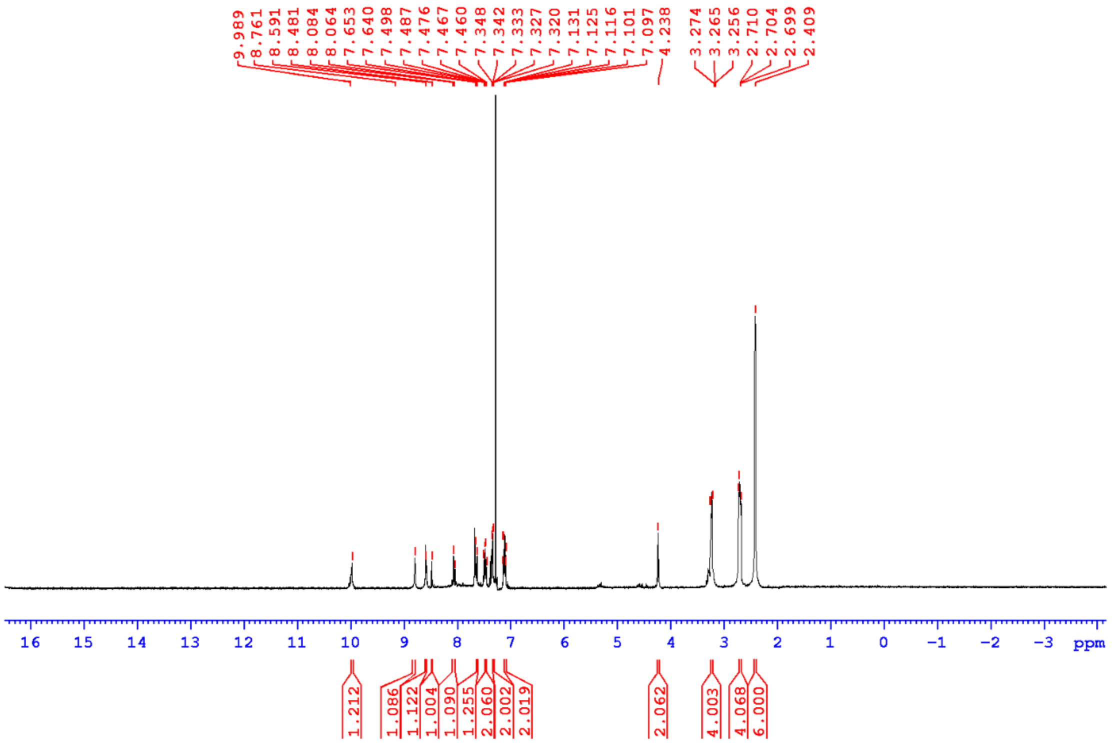

Fig S60. ^{1}HNMR Spectra. N’-[4-(quinoxaline-2-yl)-piperazine-1-yl]methyl-1-H-indole,2,3-dion-3- metformin (4a)

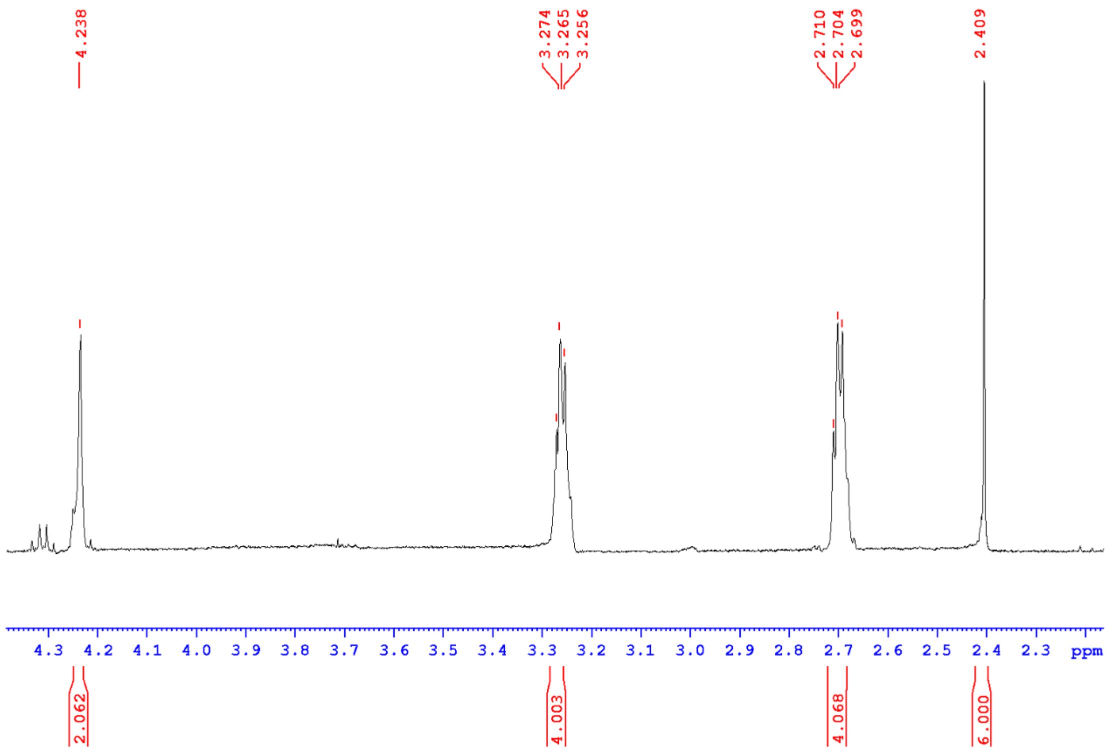

Fig S61. ^{1}HNMR Spectra. Expand- aliphatic region. N'-[4-(quinoxaline-2-yl)-piperazine-1-yl]methyl-1-H-indole,2,3-dion-3- metformin (4a)

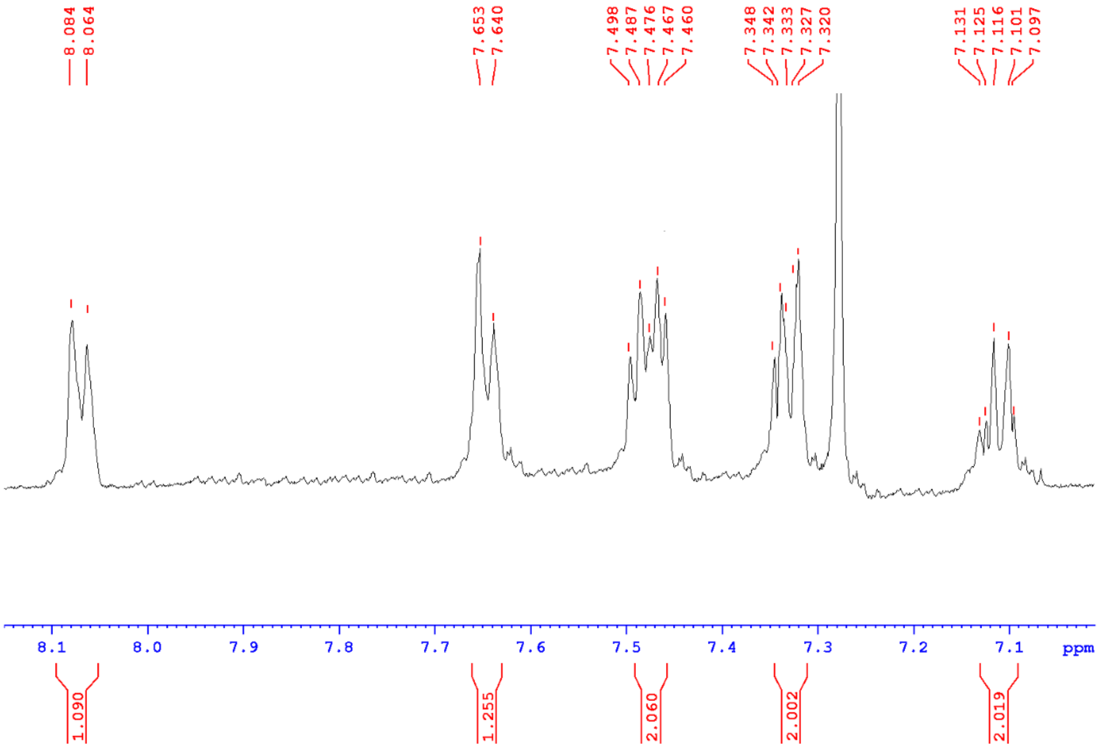

Fig S62. ^{1}HNMR Spectra. Expand- aromatic region. N'-[4-(quinoxaline-2-yl)-piperazine-1-yl]methyl-1-H-indole,2,3-dion-3- metformin (4a)

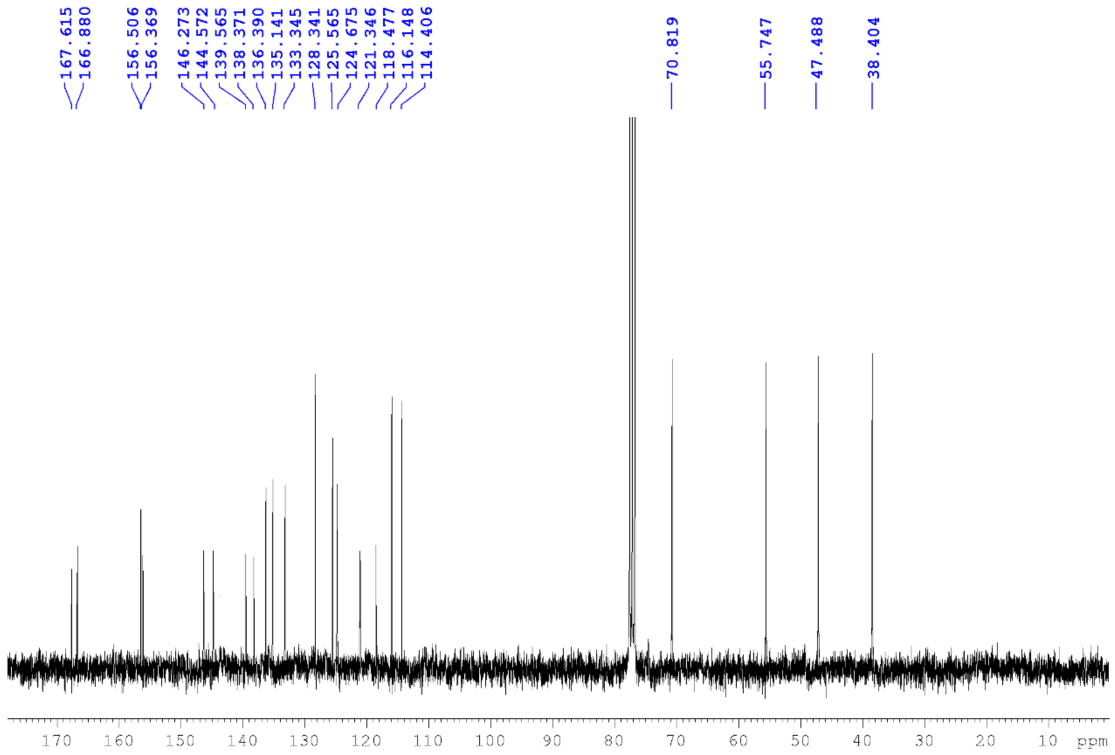

Fig S63. ^{13}CNMR Spectra. N'-[4-(quinoxaline-2-yl)-piperazine-1-yl]methyl-1-H-indole,2,3-dion-3- metformin (4a)

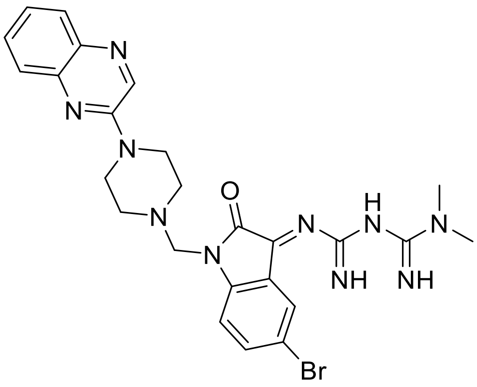

N'-[4-(quinoxaline-2-yl)-piperazine-1-yl]methyl-5-Bromo-1-H-indole,2,3-dion-3- metformin (4b)

Reddish Orangee crystals; IR (KBr, v_{max}, cm^{-1}): 3453, 3033 (N-H), 1765 (N-C=O), 2621 (Ar-CH), 1446 (C=N), 1023 (C–N), 755(C–Br) cm^{-1}; ^1H NMR (600 MHz, $CDCl_3$, DMSO) δ: δ: 2.18 (s, 6H,

CH_3, CH_3), 2.32-2.34 (m, 4H, b-2H, d-2H), 2.99-3.02 (m, 4H, a-2H, c-2H), 4.03 (s, 2H, CH_2), 7.04- 7.06 (d, j= 8, 1H, 6-H), 7.20- 7.22 (d, j= 8, 1H, 7-H), 7.31 (s, 1H, 4-H), 7.37- 7.40 (m, 2H, 6'-H, 7'-H), 7.57-7.58 (d, j= 8.8, 1H, 5’-H), 8.12-8.13 (d, j= 8.8, 1H, 8’-H), 8.30 (s, 1H, 3'-H), 8.55 (s, 1H, C=NH), 8.87 (s, 1H, C=NH), 9.82 (s, 1H, NH); ^{13}C NMR (150 MHz, $CDCl_3$, DMSO) δ: 35.30 (2C), 42.94 (2C), 53.88 (2C), 71.23, 115.83, 118.79, 119.68, 122.34, 124.40, 128.31, 129.60, 132.76, 134.84, 136.71, 138.17, 139.11, 144.85, 146.69, 156.90, 157.06 (C=NH), 162.67(C=NH), 164.18(C=O); MS: (m/z) M^+ Anal. calcd for $C_{25}H_{28}N_{10}O$: 562.1; Found: 562.1 MS: (m/z) M^+ Anal. calcd for $C_{25}H_{23b}rN_{10}O$: 562.1, Found: 562.1

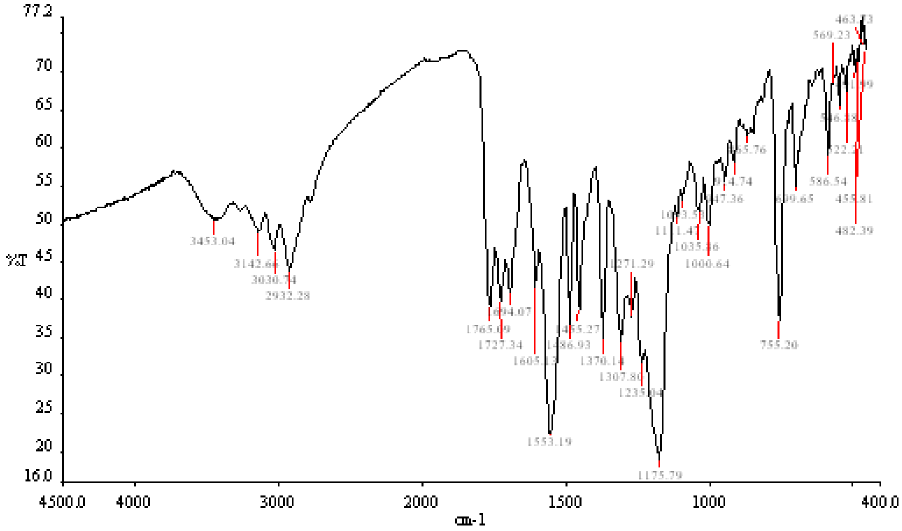

Fig S64. (FT-IR). N’-[4-(quinoxaline-2-yl)-piperazine-1-yl]methyl-5-Bromo-1-H-indole,2,3-dion-3- metformin (4b)

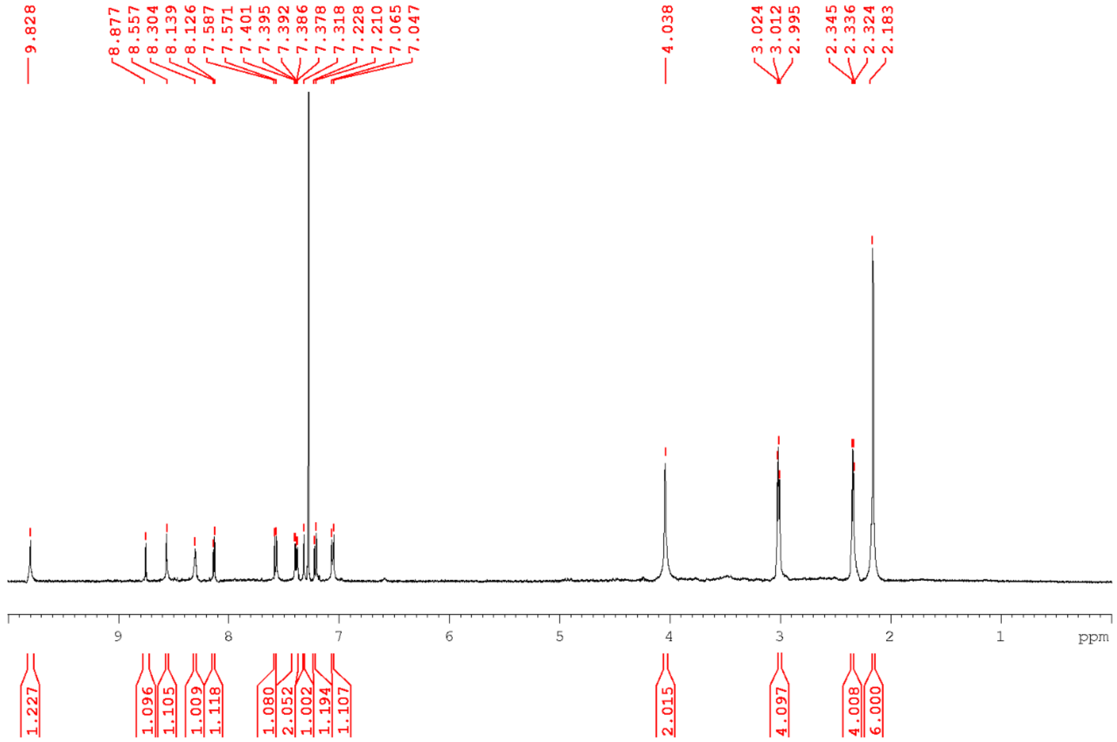

Fig S65. 1HNMR Spectra. N'-[4-(quinoxaline-2-yl)-piperazine-1-yl]methyl-5-Bromo-1-H-indole,2,3-dion-3- metformin (4b)

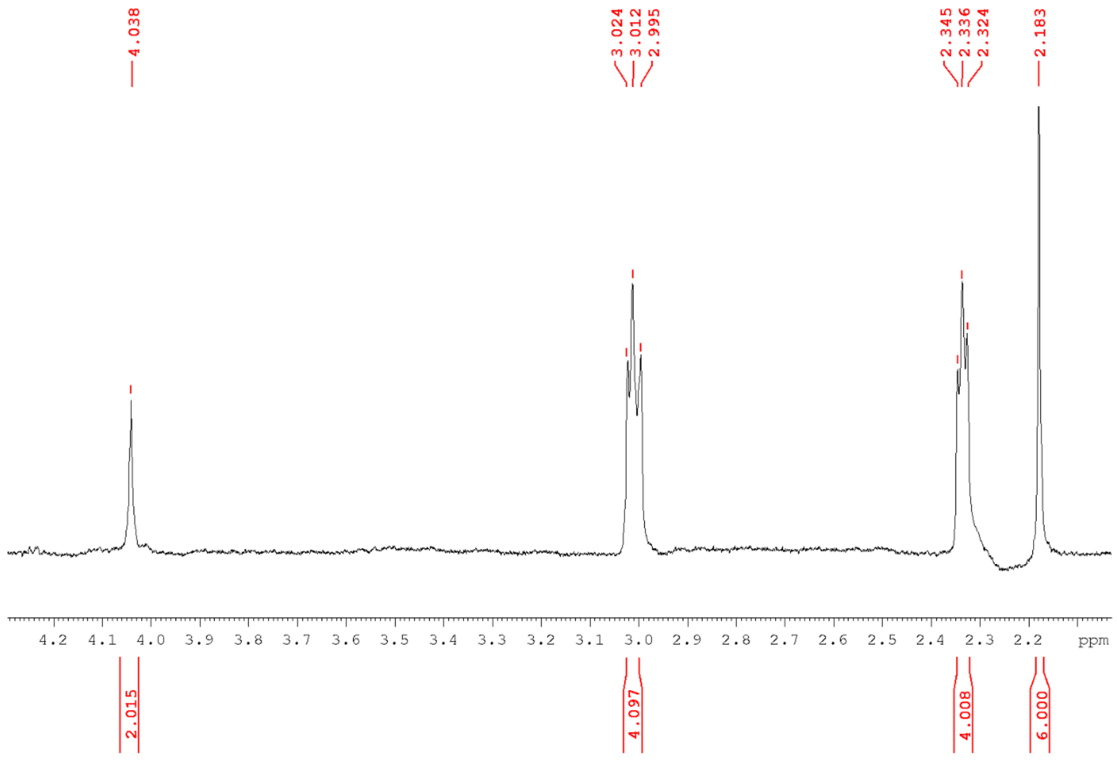

Fig S66. ^{1}HNMR Spectra. Expand- aliphatic region. N'-[4-(quinoxaline-2-yl)-piperazine-1-yl]methyl-5-Bromo-1-H-indole,2,3-dion-3- metformin (4b)

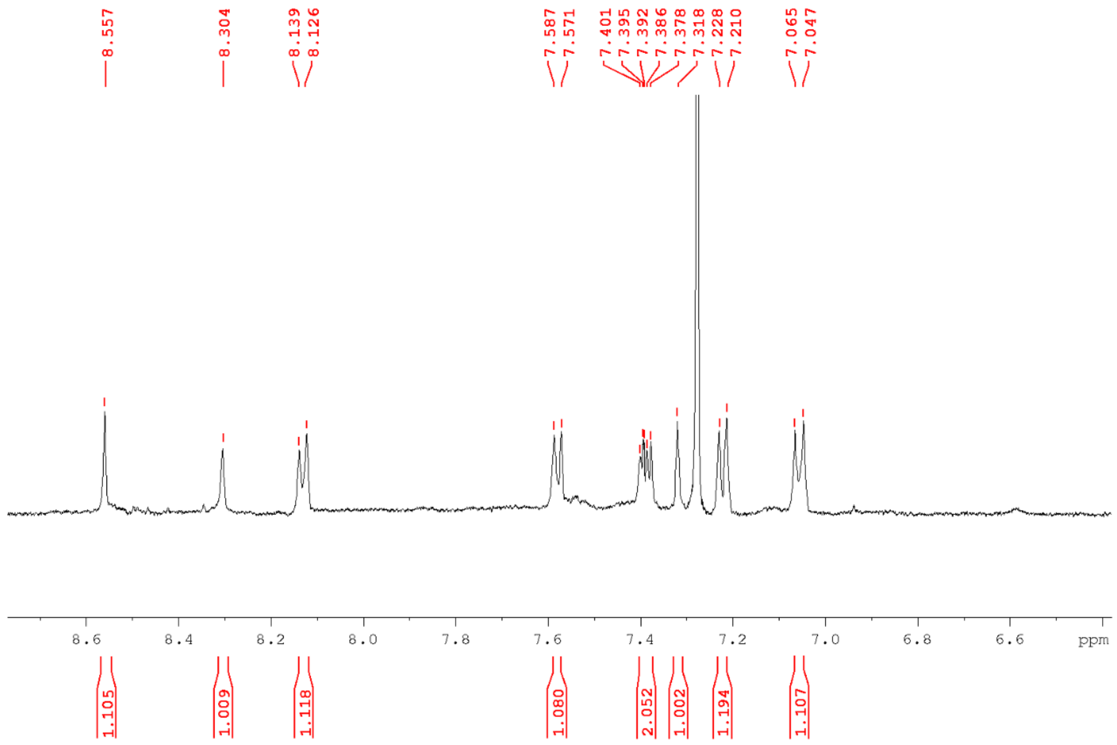

Fig S67. ^{1}HNMR Spectra. Expand- aromatic region. N'-[4-(quinoxaline-2-yl)-piperazine-1-yl]methyl-5-Bromo-1-H-indole,2,3-dion-3- metformin (4b)

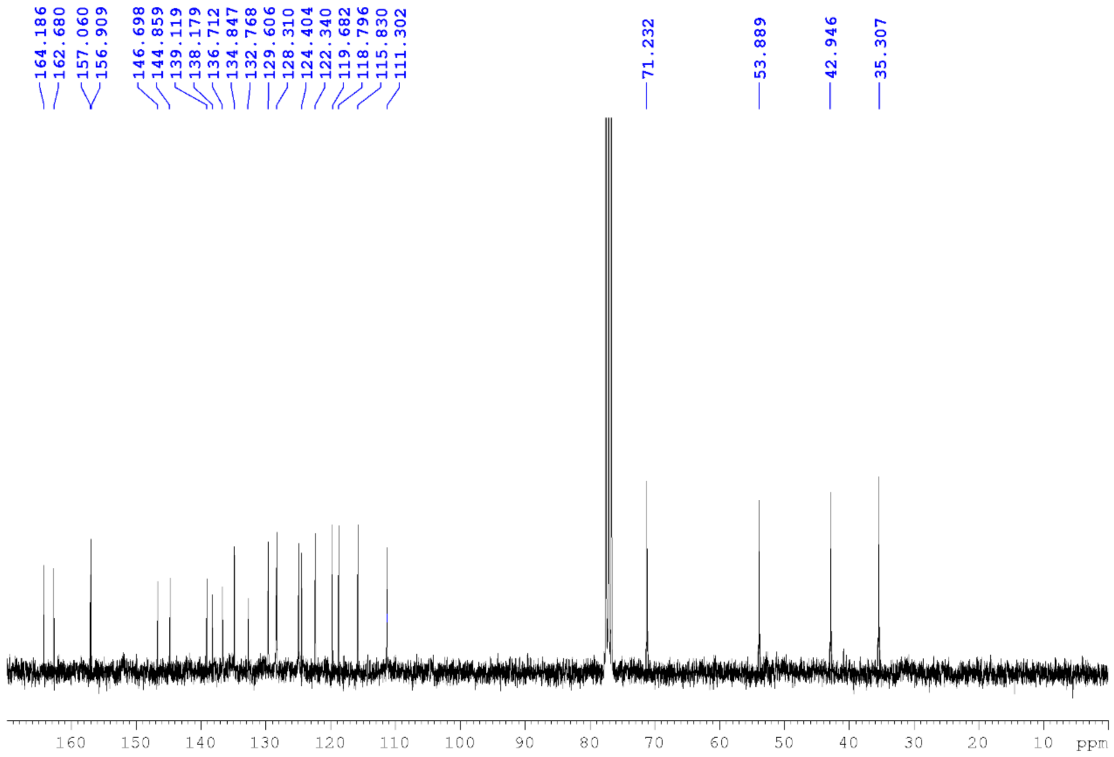

Fig S68. ^{13}CNMR Spectra. N'-[4-(quinoxaline-2-yl)-piperazine-1-yl]methyl-5-Bromo-1-H-indole,2,3-dion-3- metformin (4b)

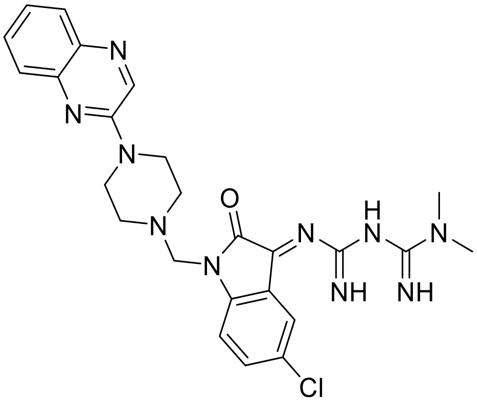

N'-[4-(quinoxaline-2-yl)-piperazine-1-yl]methyl-5-chloro-1-H-indole,2,3-dion-3- metformin (4c)

Orangee crystals; IR (KBr, v_{max}, cm^{-1}): 3436, 3067 (N-H), 1689 (N-C=O), 2632 (Ar-CH), 1464 (C=N), 1031 (C–N), 763(C–Cl) cm^{-1}; ^{1}H NMR (600 MHz, $CDCl_3$, DMSO) δ: 2.18 (s, 6H, CH_3, CH_3), 2.28-2.31 (m, 4H, b-2H, d-2H), 3.07-3.09 (m, 4H, a-2H, c-2H), 4.07 (s, 2H, CH_2), 7.06-7.08 (d, j= 8, 1H, 6-H), 7.16- 7.18 (d, j= 8, 1H, 7-H), 7.35 (s, 1H, 4-H), 7.40- 7.44 (m, 2H, 6'-H, 7'-H), 7.82-7.83 (d, j= 8.8, 1H, 5'-H), 8.06-8.07 (d, j= 8.8, 1H, 8'-H), 8.30 (s, 1H, 3'-H), 8.50 (s, 1H, C=NH), 8.78 (s, 1H, C=NH), 9.72 (s, 1H, NH); ^{13}C NMR (150 MHz, $CDCl_3$, DMSO) δ: 38.40 (2C), 48.93 (2C), 55.33 (2C), 71.43, 115.94, 117.11, 119.15, 121.33, 123.71, 126.93, 129.03, 131.68, 133.43, 136.50, 137.73, 138.34, 142.01, 143.02, 158.32, 158.43(C=NH), 161.57(C=NH), 163.99 (C=O); MS: (m/z) M+ Anal. calcd for $C_{25}H_{23cl}N_{10}O$: 518.2, Found: 518.2

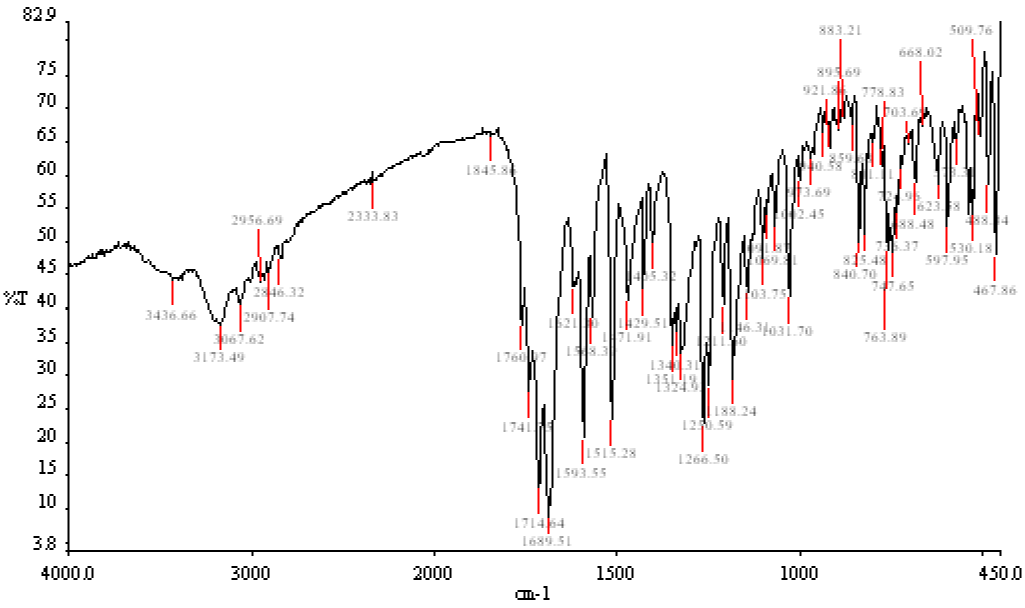

Fig S69. (FT-IR). N'-[4-(quinoxaline-2-yl)-piperazine-1-yl]methyl-5-chloro-1-H-indole,2,3-dion-3- metformin (4c)

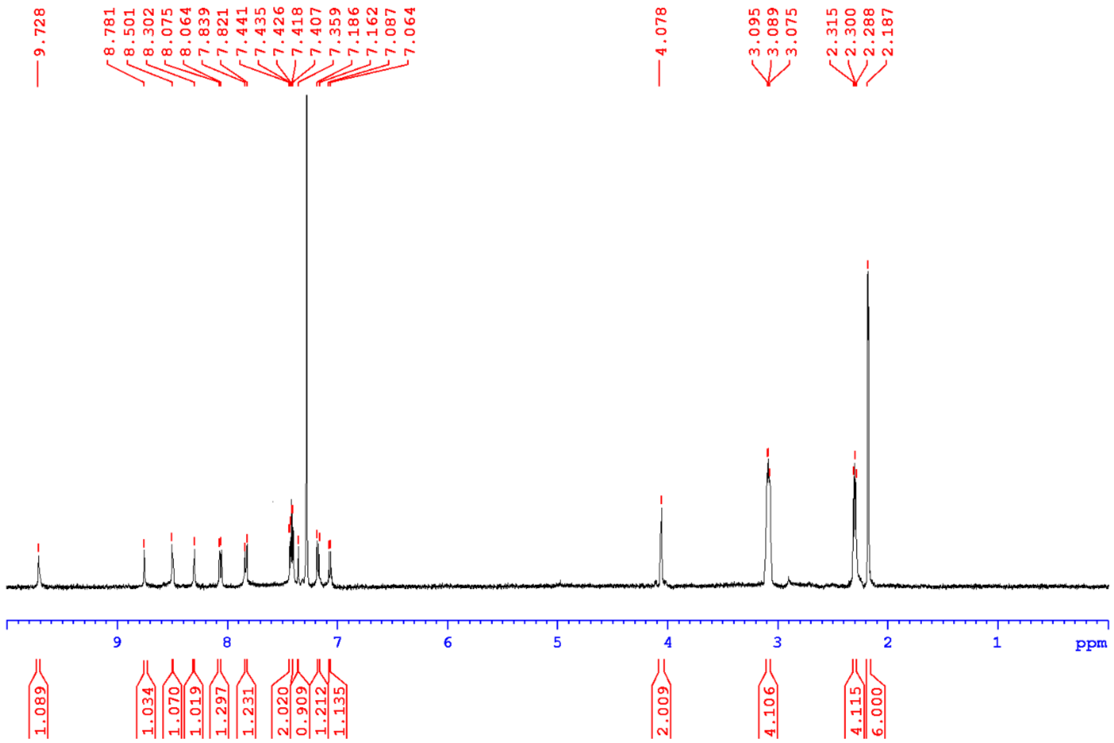

Fig S70. ^{1}HNMR Spectra. N’-[4-(quinoxaline-2-yl)-piperazine-1-yl]methyl-5-chloro-1-H-indole,2,3-dion-3- metformin (4c)

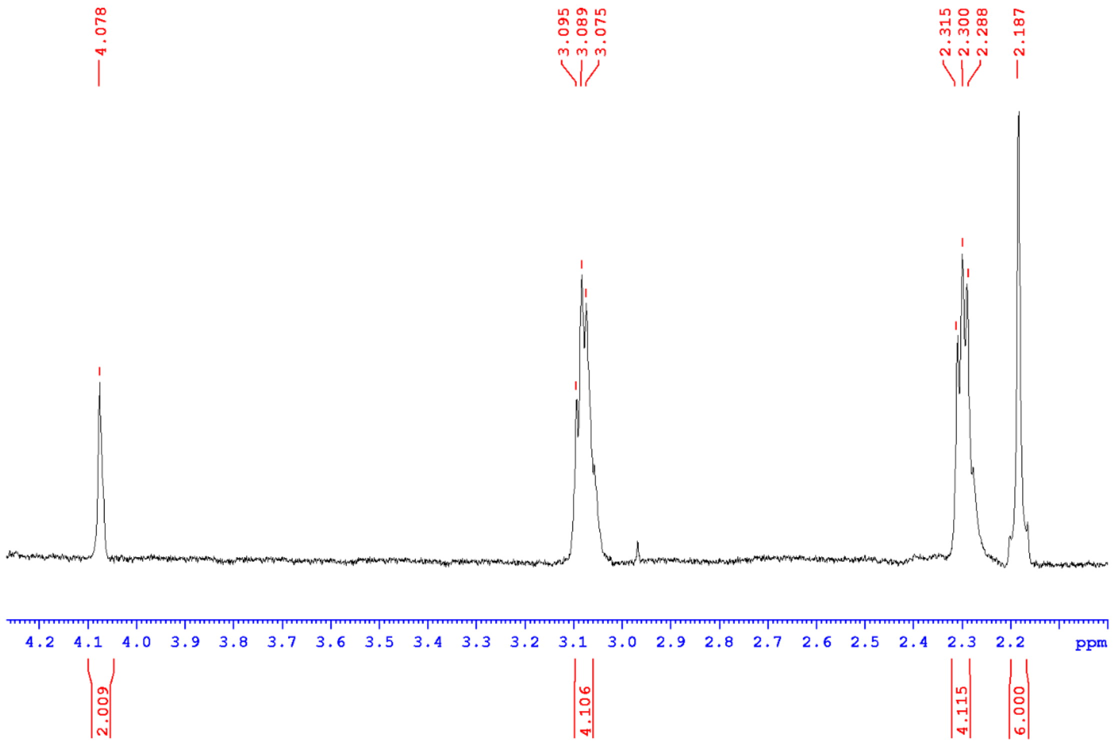

Fig S71. ^{1}HNMR Spectra. Expand- aliphatic region. N'-[4-(quinoxaline-2-yl)-piperazine-1-yl]methyl-5-chloro-1-H-indole,2,3-dion-3- metformin (4c)

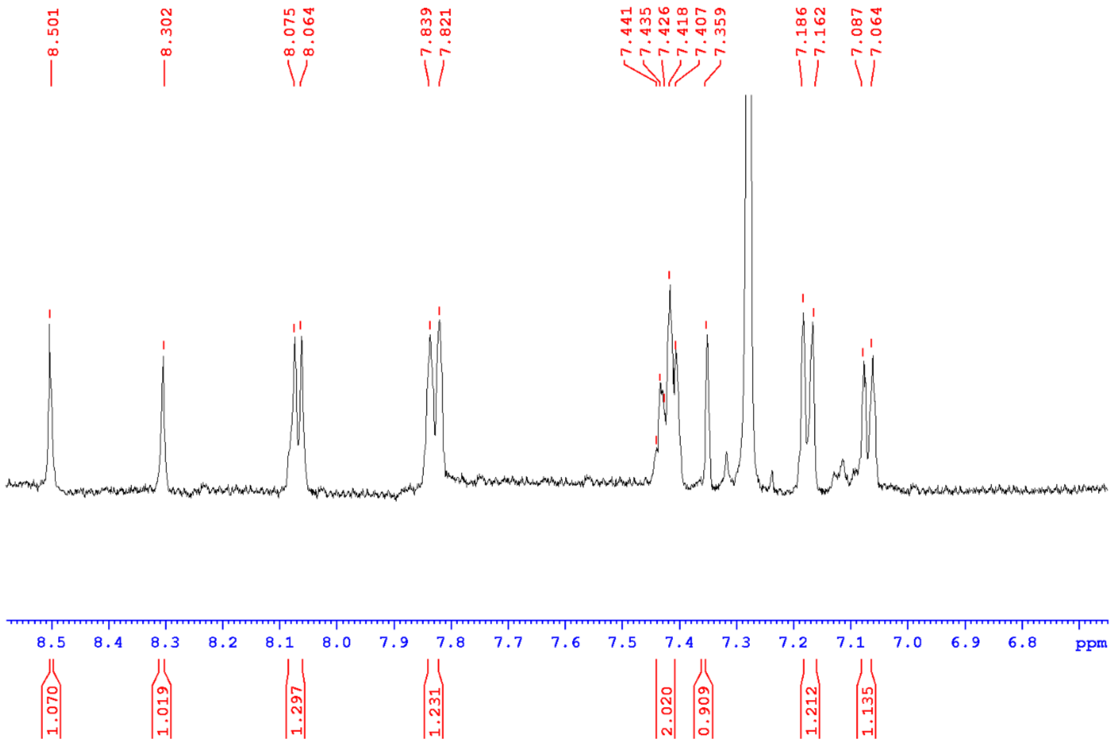

Fig S72. ^{1}HNMR Spectra. Expand- aromatic region. N'-[4-(quinoxaline-2-yl)-piperazine-1-yl]methyl-5-chloro-1-H-indole,2,3-dion-3- metformin (4c)

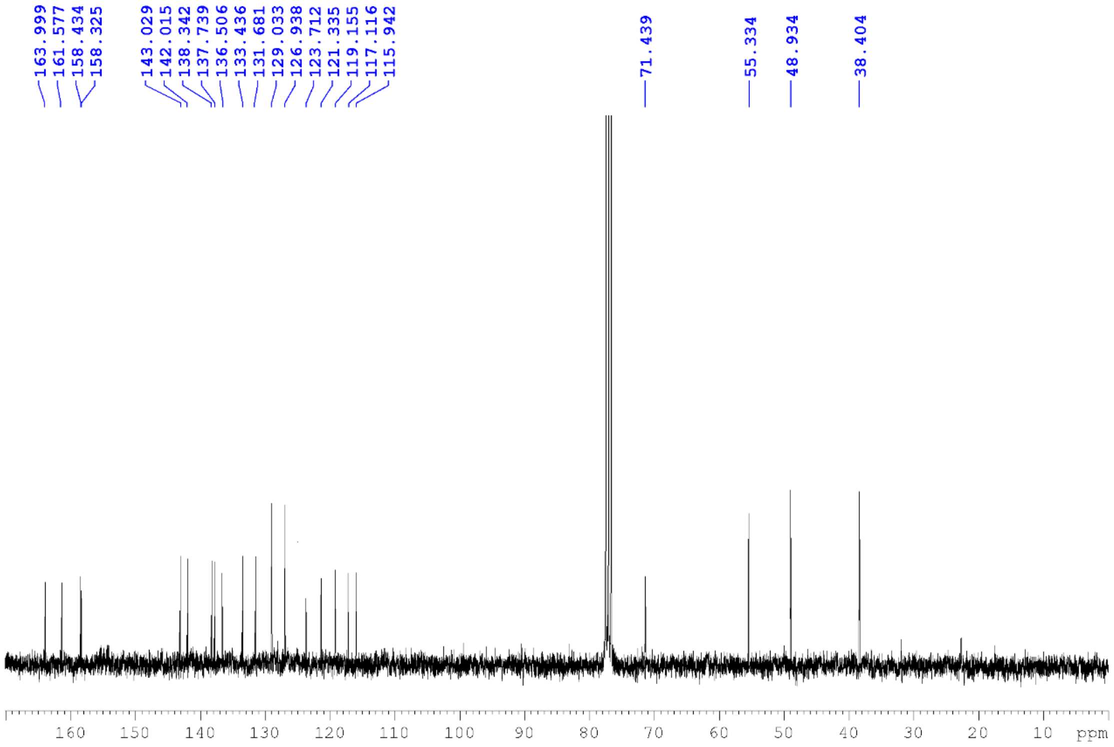

Fig S73. ^{13}CNMR Spectra. N'-[4-(quinoxaline-2-yl)-piperazine-1-yl]methyl-5-chloro-1-H-indole,2,3-dion-3- metformin (4c)

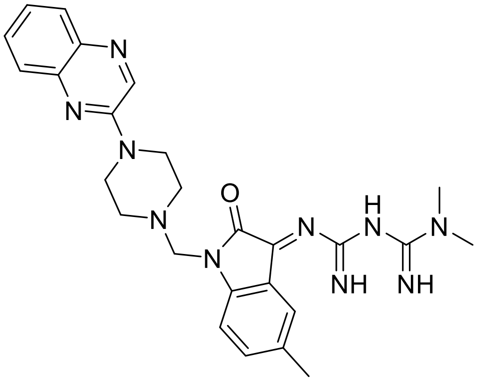

N'-[4-(quinoxaline-2-yl)-piperazine-1-yl]methyl-5-methyl-1-H-indole,2,3-dion-3- metformin (4d)

Yellow crystals; IR (KBr, v_{max}, cm^{-1}): 3480, (N-H), 1749 (N-C=O), 2703 (Ar-CH), 1388 (C=N), 1012 (C–N) cm^{-1}; ^{1}H NMR (600 MHz, $CDCl_3$, DMSO) δ: 2.19 (s, 6H, CH_3, CH_3), 2.35 (s, 3H,

CH_3-Isatin), 2.88-2.90 (m, 4H, b-2H, d-2H), 3.08- 3.10 (m, 4H, a-2H, c-2H), 4.38 (s, 2H, CH_2), 7.09- 7.11 (d, j= 8, 1H, 6-H), 7.24- 7.26 (d, j= 8, 1H, 7-H), 7.37 (s, 1H, 4-H), 7.63- 7.67 (m, 2H, 6'-H, 7'-H), 7.82-7.84 (d, j= 8.8, 1H, 5'-H), 8.16-8.18 (d, j= 8.8, 1H, 8'-H), 8.32 (s, 1H, 3'-H), 8.45 (s, 1H, C=NH), 8.80 (s, 1H, C=NH), 9.69 (s, 1H, NH); ^{13}C NMR (150 MHz, $CDCl_3$, DMSO) δ: 23.82, 38.81 (2C), 47.07 (2C), 55.95 (2C), 72.67, 115.40, 117.17, 119.57, 123.64, 125.11, 126.19, 127.84, 133.79, 134.84, 135.41, 136.80, 139.23, 141.80, 141.49, 154.53, 154.68(C=NH), 162.07(C=NH), 164.18 (C=O); MS: (m/z) M^+ Anal. calcd for $C_{26}H_{30}N_{10}O$: 498.2, Found: 498.2

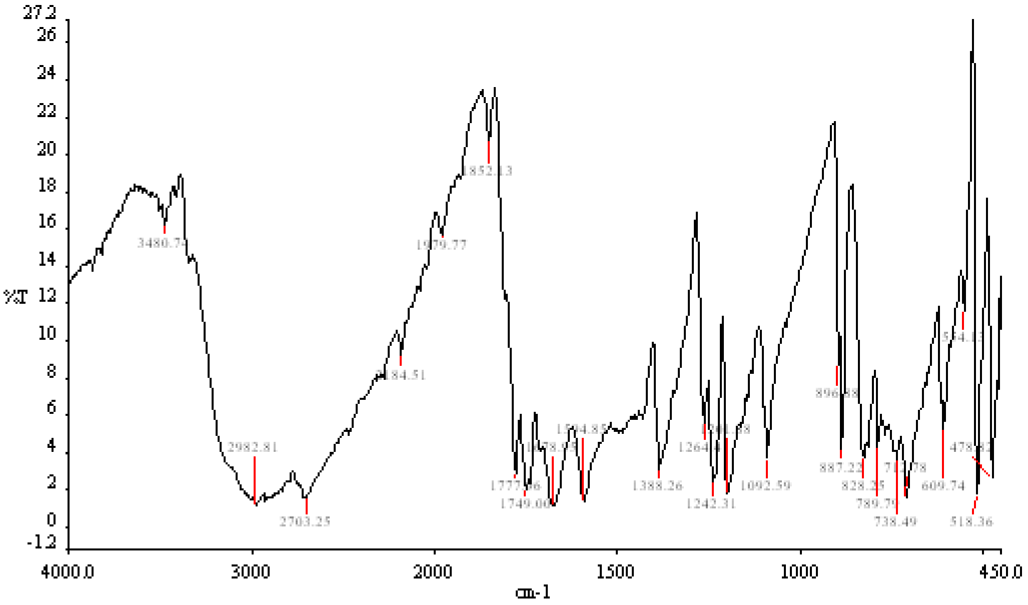

Fig S74. (FT-IR). N'-[4-(quinoxaline-2-yl)-piperazine-1-yl]methyl-5-methyl-1-H-indole,2,3-dion-3- metformin (4d)

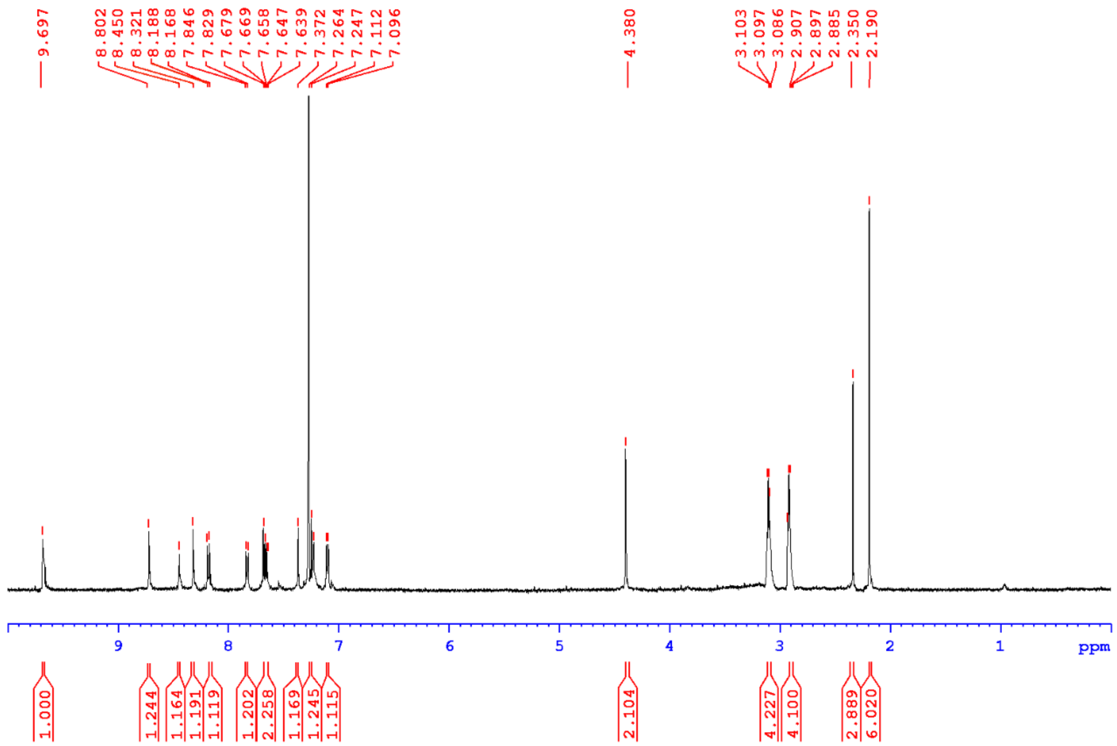

Fig S75. ^{1}HNMR Spectra. N'-[4-(quinoxaline-2-yl)-piperazine-1-yl]methyl-5-methyl-1-H-indole,2,3-dion-3- metformin (4d)

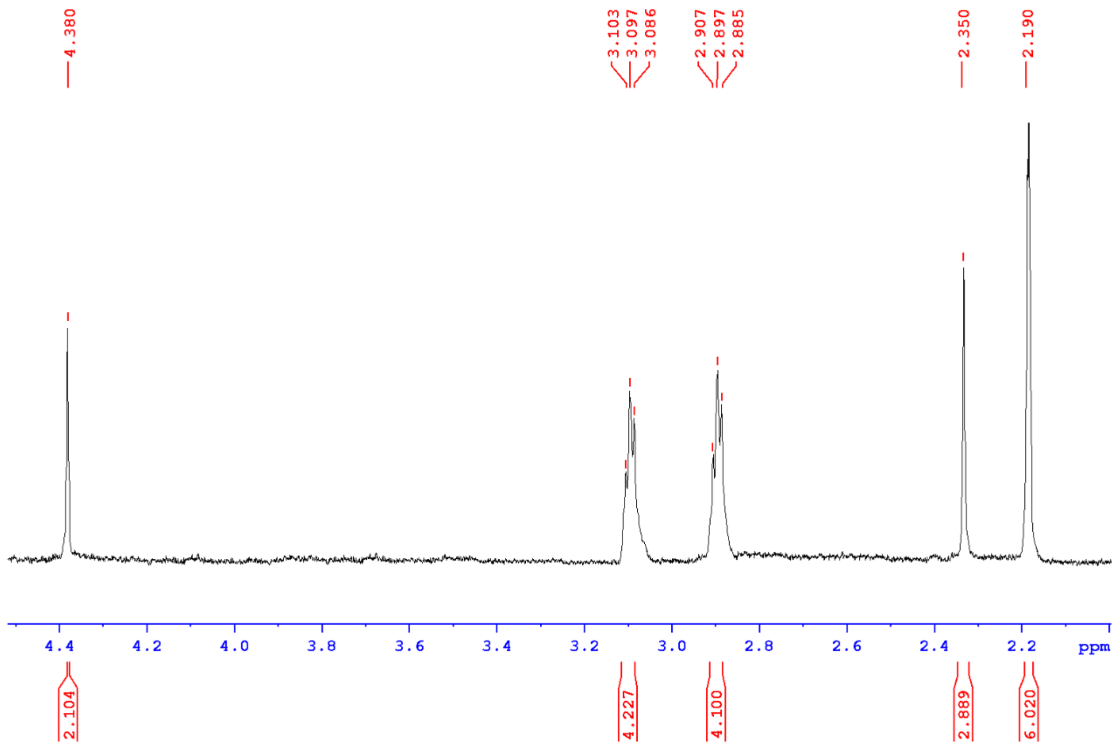

Fig S76. ^{1}HNMR Spectra. Expand- aliphatic region. N'-[4-(quinoxaline-2-yl)-piperazine-1-yl]methyl-5-methyl-1-H-indole,2,3-dion-3- metformin (4d)

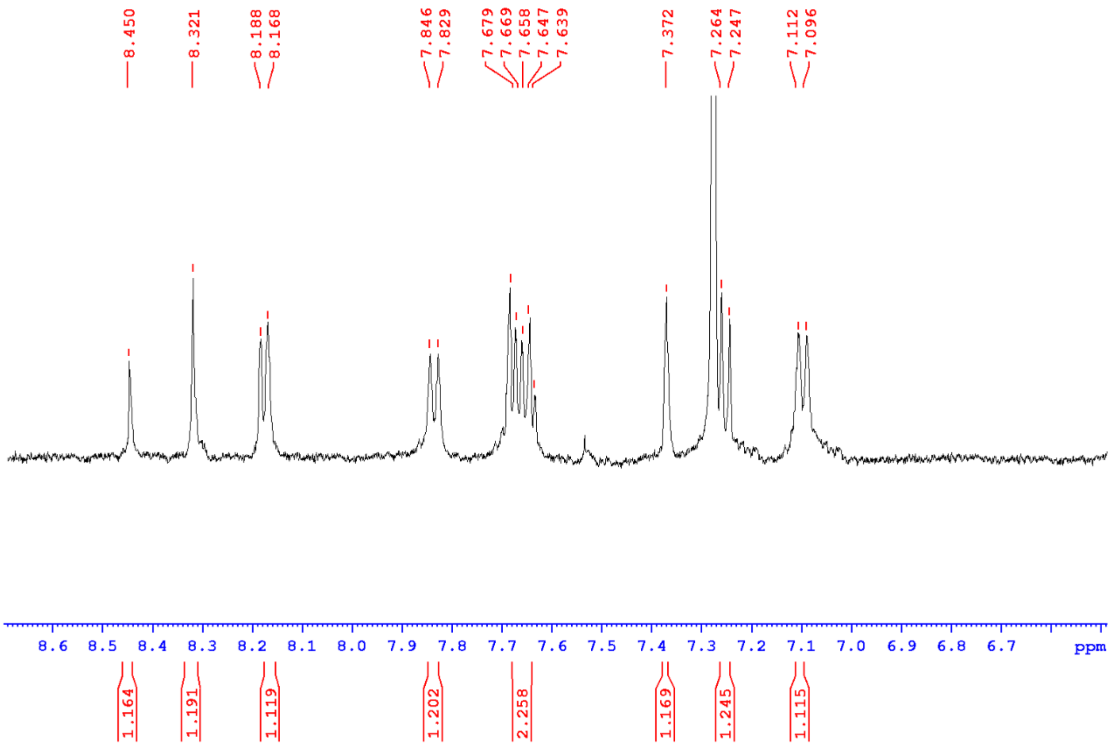

Fig S77. ¹HNMR Spectra. Expand- aromatic region. N'-[4-(quinoxaline-2-yl)-piperazine-1-yl]methyl-5-methyl-1-H-indole,2,3-dion-3- metformin (4d)

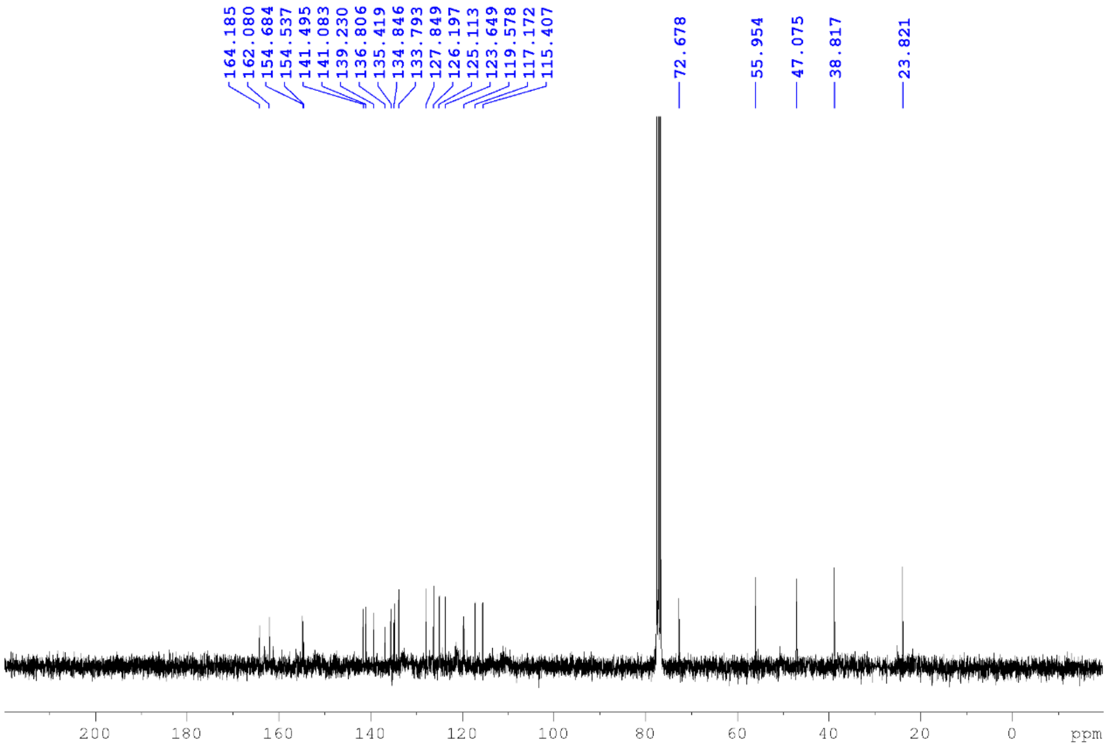

Fig S78. ^{13}CNMR Spectra. N'-[4-(quinoxaline-2-yl)-piperazine-1-yl]methyl-5-methyl-1-H-indole,2,3-dion-3- metformin (4d)

References

1- Esam Z, Akhavan M, Bekhradnia A. One-pot multicomponent synthesis of novel 2-(Piperazin-1-yl) quinoxaline and benzimidazole derivatives, using a novel sulfamic acid functionalized Fe3O4 MNPs as highly effective nanocatalyst. Appl Organomet Chem. 2020; e6005. https://doi.org/10.1002/aoc.6005

2- Omolbanin Shahraki, Farshid Zargari, Najmeh Edraki, Mehdi Khoshneviszadeh, Omidreza Firuzi & Ramin Miri (2018) Molecular dynamics simulation and molecular docking studies of 1,4-Dihydropyridines as P-glycoprotein's allosteric inhibitors, Journal of Biomolecular Structure and Dynamics, 36:1, 112-125,

3- Wang, Zhe and Sun, Huiyong and Yao, Xiaojun and Li, Dan and Xu, Lei and Li, Youyong and Tian, Sheng and Hou, Tingjun, Comprehensive evaluation of ten docking programs on a diverse set of protein–ligand complexes: the prediction accuracy of sampling power and scoring power, Phys. Chem. Chem. Phys., 2016, 18,18, 12964-12975

4- Mol CD, Dougan DR, Schneider TR, Skene RJ, Kraus ML, Scheibe DN, Snell GP, Zou H, Sang BC, Wilson KP. Structural basis for the autoinhibition and STI-571 inhibition of c-Kit tyrosine kinase. Journal of Biological Chemistry. 2004 Jul 23;279(30):31655-63.

5- M. H. Knaggs, F. R. Salsbury, M. H. Edgell, J. S. Fetrow. Insights into correlated motions and long-range interactions in CheY derived from molecular dynamics simulations. Biophys. J., 92 (2007) 2062-79.

6- X. Daura, K. Gademann, B. Jaun, D. Seebach, W. F. van Gunsteren, A. E. Mark. Peptidfaltung: Wenn die Simulation das Experiment erreicht. Angew. Chem., 111 (1999) 249-53.

7- Farshid Zargari, Maryam Lotfi, Omolbanin Shahraki, Zahra Nikfarjam & Jafar Shahraki (2018) Flavonoids as potent allosteric inhibitors of protein tyrosine phosphatase 1B: molecular dynamics simulation and free energy calculation, Journal of Biomolecular Structure and Dynamics, 36:15, 4126-4142.

8- Iftime MM, Cozan V, Airinei A, Varganici C, Ailiesei G, Timpu D, Sava I. Asymmetric azomethine amines with azobenzene moieties–liquid crystalline and optical properties. Liquid Crystals. 2019 Aug 9;46(10):1584-94.

9- Hočevar T, Demšar J. Computation of graphlet orbits for nodes and edges in sparse graphs. Journ. Stat. Soft. 2016 Jul 28;71.

10- Morris GM, Goodsell DS, Halliday RS, Huey R, Hart WE, Belew RK, Olson AJ. Automated docking using a Lamarckian genetic algorithm and an empirical binding free energy function. Journal of computational chemistry. 1998 Nov 15;19(14):1639-62.

11- Aduri R, Psciuk BT, Saro P, Taniga H, Schlegel HB, SantaLucia J. AMBER force field parameters for the naturally occurring modified nucleosides in RNA. Journal of Chemical Theory and Computation. 2007 Jul 10;3(4):1464-75.

12- Vanquelef E, Simon S, Marquant G, Garcia E, Klimerak G, Delepine JC, Cieplak P, Dupradeau FY. RED Server: a web service for deriving RESP and ESP charges and building force field libraries for new molecules and molecular fragments. Nucleic acids research. 2011 Jul 1;39(suppl_2):W511-7.

13- Da Silva AW, Vranken WF. ACPYPE-Antechamber python parser interface. BMC research notes. 2012 Dec 1;5(1):367.

14- D. Van Der Spoel, E. Lindahl, B. Hess, G. Groenhof, A. E. Mark, H. J. Berendsen. GROMACS: fast, flexible, and free. J. Comput. Chem., 26 (2005) 1701-18.
15- K. A. Beauchamp, Y.-S. Lin, R. Das, V. S. Pande. Are protein force fields getting better? A systematic benchmark on 524 diverse NMR measurements. Journal of chemical theory and computation, 8 (2012) 1409-14.
16- J. Berendsen, J. v. Postma, W. F. van Gunsteren, A. DiNola, J. Haak. Molecular dynamics with coupling to an external bath. The Journal of chemical physics, 81 (1984) 3684-90.
17- Humphrey W, Dalke A, Schulten K. VMD: visual molecular dynamics. Journal of molecular graphics. 1996 Feb 1;14(1):33-8.
18- Darden T, York D, Pedersen L. Particle mesh Ewald: An N· log (N) method for Ewald sums in large systems. The Journal of chemical physics. 1993 Jun 15;98(12):10089-92.
19- Hess B, Bekker H, Berendsen HJ, Fraaije JG. LINCS: a linear constraint solver for molecular simulations. Journal of computational chemistry. 1997 Sep;18(12):1463-72.
20- Hernández-Santoyo A, Tenorio-Barajas AY, Altuzar V, Vivanco-Cid H, Mendoza-Barrera C. Protein-protein and protein-ligand docking. Protein engineering-technology and application. 2013 May 29:66.
21- Isca VM, Ferreira RJ, Garcia C, Monteiro CM, Dinic J, Holmstedt S, André V, Pesic M, dos Santos DJ, Candeias NR, Afonso CA. Molecular Docking Studies of Royleanone Diterpenoids from Plectranthus spp. as P-Glycoprotein Inhibitors. ACS Medicinal Chemistry Letters. 2020 Mar 12;11(5):839-45.
22- Palestro PH, Gavernet L, Estiu GL, Bruno Blanch LE. Docking Applied to the Prediction of the Affinity of Compounds to P-Glycoprotein. BioMed research international. 2014 Jan 1;2014.
23- DeLano WL. Pymol: An open-source molecular graphics tool. CCP4 Newsletter on protein crystallography. 2002 Mar;40(1):82-92.
24- James, J. P. (2016). MOPAC2016, Stewart Computational Chemistry
25- Zhang N, Zhao H. Enriching screening libraries with bioactive fragment space. Bioorganic & medicinal chemistry letters. 2016 Aug 1;26(15):3594-7.
